# Multi-platform discovery of haplotype-resolved structural variation in human genomes

**DOI:** 10.1038/s41467-018-08148-z

**Published:** 2019-04-16

**Authors:** Mark J. P. Chaisson, Ashley D. Sanders, Xuefang Zhao, Ankit Malhotra, David Porubsky, Tobias Rausch, Eugene J. Gardner, Oscar L. Rodriguez, Li Guo, Ryan L. Collins, Xian Fan, Jia Wen, Robert E. Handsaker, Susan Fairley, Zev N. Kronenberg, Xiangmeng Kong, Fereydoun Hormozdiari, Dillon Lee, Aaron M. Wenger, Alex R. Hastie, Danny Antaki, Thomas Anantharaman, Peter A. Audano, Harrison Brand, Stuart Cantsilieris, Han Cao, Eliza Cerveira, Chong Chen, Xintong Chen, Chen-Shan Chin, Zechen Chong, Nelson T. Chuang, Christine C. Lambert, Deanna M. Church, Laura Clarke, Andrew Farrell, Joey Flores, Timur Galeev, David U. Gorkin, Madhusudan Gujral, Victor Guryev, William Haynes Heaton, Jonas Korlach, Sushant Kumar, Jee Young Kwon, Ernest T. Lam, Jong Eun Lee, Joyce Lee, Wan-Ping Lee, Sau Peng Lee, Shantao Li, Patrick Marks, Karine Viaud-Martinez, Sascha Meiers, Katherine M. Munson, Fabio C. P. Navarro, Bradley J. Nelson, Conor Nodzak, Amina Noor, Sofia Kyriazopoulou-Panagiotopoulou, Andy W. C. Pang, Yunjiang Qiu, Gabriel Rosanio, Mallory Ryan, Adrian Stütz, Diana C. J. Spierings, Alistair Ward, AnneMarie E. Welch, Ming Xiao, Wei Xu, Chengsheng Zhang, Qihui Zhu, Xiangqun Zheng-Bradley, Ernesto Lowy, Sergei Yakneen, Steven McCarroll, Goo Jun, Li Ding, Chong Lek Koh, Bing Ren, Paul Flicek, Ken Chen, Mark B. Gerstein, Pui-Yan Kwok, Peter M. Lansdorp, Gabor T. Marth, Jonathan Sebat, Xinghua Shi, Ali Bashir, Kai Ye, Scott E. Devine, Michael E. Talkowski, Ryan E. Mills, Tobias Marschall, Jan O. Korbel, Evan E. Eichler, Charles Lee

**Affiliations:** 1 Department of Genome Sciences, University of Washington School of Medicine, Seattle, WA 98195 USA; 2 Quantitative and Computational Biology, University of Southern California, Los Angeles, CA 90089 USA; 3 European Molecular Biology Laboratory, Genome Biology Unit, 69117 Heidelberg, Germany; 4 Department of Computational Medicine and Bioinformatics, University of Michigan, Ann Arbor, MI 48109 USA; 5 Center for Genomic Medicine, Massachusetts General Hospital, Department of Neurology, Harvard Medical School, Boston, MA 02114 USA; 6 The Jackson Laboratory for Genomic Medicine, Farmington, CT 06032 USA; 7 European Research Institute for the Biology of Ageing, University of Groningen, University Medical Centre Groningen, Groningen, AV NL-9713 The Netherlands; 8 Center for Bioinformatics, Saarland University and the Max Planck Institute for Informatics, 66123 Saarbrücken, Germany; 9 Institute for Genome Sciences, University of Maryland School of Medicine, Baltimore, MD 21201 USA; 10 Department of Genetics and Genomic Sciences, Icahn School of Medicine at Mount Sinai, New York, NY 10029 USA; 11 The School of Life Science and Technology of Xi’an Jiaotong University, 710049 Xi’an, China; 12 MOE Key Lab for Intelligent Networks & Networks Security, School of Electronics and Information Engineering, Xi’an Jiaotong University, 710049 Xi’an, China; 13 Ye-Lab For Omics and Omics Informatics, Xi’an Jiaotong University, 710049 Xi’an, China; 14 Program in Bioinformatics and Integrative Genomics, Harvard Medical School, Boston, MA 02115 USA; 15 Department of Bioinformatics and Computational Biology, The University of Texas MD Anderson Cancer Center, Houston, TX 77030 USA; 16 Department of Bioinformatics and Genomics, College of Computing and Informatics, The University of North Carolina at Charlotte, Charlotte, NC 28223 USA; 17 Department of Genetics, Harvard Medical School, Boston, MA 02115 USA; 18 The Stanley Center for Psychiatric Research, Broad Institute of MIT and Harvard, Cambridge, MA 02142 USA; 19 Program in Medical and Population Genetics, Broad Institute of MIT and Harvard, Cambridge, MA 02142 USA; 20 European Molecular Biology Laboratory, European Bioinformatics Institute, Wellcome Genome Campus, Hinxton, Cambridge, CB10 1SD United Kingdom; 21 Yale University Medical School, Computational Biology and Bioinformatics Program, New Haven, CT 06520 USA; 22 Department of Molecular Biophysics and Biochemistry, Yale University, 266 Whitney Avenue, New Haven, CT 06520 USA; 23 Biochemistry and Molecular Medicine, University of California Davis, Davis, CA 95616 USA; 24 UC Davis Genome Center, University of California, Davis, Davis, CA 95616 USA; 25 USTAR Center for Genetic Discovery and Department of Human Genetics, University of Utah School of Medicine, Salt Lake City, UT 84112 USA; 26 Pacific Biosciences, Menlo Park, CA 94025 USA; 27 Bionano Genomics, San Diego, CA 92121 USA; 28 Beyster Center for Genomics of Psychiatric Diseases, Department of Psychiatry University of California San Diego, La Jolla, CA 92093 USA; 29 10X Genomics, Pleasanton, CA 94566 USA; 30 Illumina Clinical Services Laboratory, Illumina, Inc., 5200 Illumina Way, San Diego, CA 92122 USA; 31 Department of Cellular and Molecular Medicine, University of California San Diego, La Jolla, CA 92093 USA; 32 Ludwig Institute for Cancer Research, La Jolla, CA 92093 USA; 33 Department of Graduate Studies – Life Sciences, Ewha Womans University, 52, Ewhayeodae-gil, Seodaemun-gu, Seoul, 03760 South Korea; 34 DNA Link, Seodaemun-gu, Seoul, South Korea; 35 TreeCode Sdn Bhd, Bandar Botanic, 41200 Klang, Malaysia; 36 Bioinformatics and Systems Biology Graduate Program, University of California, San Diego, La Jolla, CA 92093 USA; 37 School of Biomedical Engineering, Drexel University, Philadelphia, PA 19104 USA; 38 Program in Medical and Population Genetics, Broad Institute of MIT and Harvard, Cambridge, MA 02142 USA; 39 Human Genetics Center, School of Public Health, The University of Texas Health Science Center at Houston, Houston, TX 77225 USA; 40 Department of Medicine, McDonnell Genome Institute, Siteman Cancer Center, Washington University School of Medicine, St. Louis, MI 63108 USA; 41 High Impact Research, University of Malaya, 50603 Kuala Lumpur, Malaysia; 42 Department of Computer Science, Yale University, 266 Whitney Avenue, New Haven, CT 06520 USA; 43 Department of Statistics and Data Science, Yale University, 266 Whitney Avenue, New Haven, CT 06520 USA; 44 Institute for Human Genetics, University of California–San Francisco, San Francisco, CA 94143 USA; 45 Terry Fox Laboratory, BC Cancer Agency, Vancouver, BC V5Z 1L3 Canada; 46 Department of Medical Genetics, University of British Columbia, Vancouver, BC V6T 1Z4 Canada; 47 Department of Pediatrics, University of California San Diego, La Jolla, CA 92093 USA; 48 The First Affiliated Hospital of Xi’an Jiaotong University, 710061 Xi’an, China; 49 Center for Mendelian Genomics, Broad Institute of MIT and Harvard, Cambridge, MA 02142 USA; 50 Department of Human Genetics, University of Michigan, Ann Arbor, MI 48109 USA; 51 Howard Hughes Medical Institute, University of Washington, Seattle, WA 98195 USA

## Abstract

The incomplete identification of structural variants (SVs) from whole-genome sequencing data limits studies of human genetic diversity and disease association. Here, we apply a suite of long-read, short-read, strand-specific sequencing technologies, optical mapping, and variant discovery algorithms to comprehensively analyze three trios to define the full spectrum of human genetic variation in a haplotype-resolved manner. We identify 818,054 indel variants (<50 bp) and 27,622 SVs (≥50 bp) per genome. We also discover 156 inversions per genome and 58 of the inversions intersect with the critical regions of recurrent microdeletion and microduplication syndromes. Taken together, our SV callsets represent a three to sevenfold increase in SV detection compared to most standard high-throughput sequencing studies, including those from the 1000 Genomes Project. The methods and the dataset presented serve as a gold standard for the scientific community allowing us to make recommendations for maximizing structural variation sensitivity for future genome sequencing studies.

## Abstract (web summary)

Structural variants (SVs) in human genomes contribute diversity and diseases. Here, the authors use a multi-platform strategy to generate haplotype-resolved SVs for three human parent–child trios.

## Introduction

Structural variants (SVs) contribute greater diversity at the nucleotide level between two human genomes than any other form of genetic variation^1–4^. To date, such variation has been difficult to uniformly identify and characterize from the large number of human genomes that have been sequenced using short-read, high-throughput sequencing technologies. The methods to detect SVs in these datasets are dependent, in part, on indirect inferences (e.g., read-depth and discordant read-pair mapping). The limited number of SVs observed directly using split-read algorithms^5,6^ is constrained by the short length of these sequencing reads. Moreover, while larger copy number variants (CNVs) could be identified using microarray and read-depth algorithms, smaller events (<5 kb) and balanced events, such as inversions, remain poorly ascertained^4,7^.

One fundamental problem for SV detection using short-read sequencing alone is inherent to the predominant data type: paired-end sequences of relatively short fragments that are aligned to a consensus reference. Hence, SV detection algorithms for this data type can thus be effective in unique sequences, but break down within repetitive DNA, which is highly enriched for SVs^8^. Another fundamental problem is that most SV discovery methods do not indicate which haplotype background a given SV resides on. Nevertheless, SVs are threefold more likely to associate with a genome-wide association study signal than single-nucleotide variants (SNVs), and larger SVs (>20 kb) are up to 50-fold more likely to affect the expression of a gene compared to an SNV^9^. Hence, SVs that remain cryptic to current sequencing algorithms likely represent an important source of disease-causing variation in unsolved Mendelian disorders and a component of the missing heritability in complex disorders^10^.

As part of the Human Genome Structural Variation Consortium (HGSVC), we sought to comprehensively determine the complete spectrum of human genetic variation in three family trios. To overcome the barriers to SV detection from conventional algorithms, we integrate a suite of cutting-edge genomic technologies that, when used collectively, allow SVs to be comprehensively assessed in a haplotype-aware manner in diploid genomes. In addition, we also identify the optimal combination of technologies and algorithms that would maximize sensitivity and specificity for SV detection for future genomic studies.

## Results

The goal of this study was to comprehensively discover, sequence-resolve, and phase all non-single-nucleotide variation in a selected number of human genomes. We chose three parent–child trios (mother, father, and child) for comprehensive SV discovery: a Han Chinese (CHS) trio (HG00513, HG00512, and HG00514), a Puerto Rican (PUR) trio (HG00732, HG00731, and HG00733) and a Yoruban (YRI) Nigerian trio (NA19238, NA19239, and NA19240). The Han Chinese and Yoruban Nigerian families were representative of low and high genetic diversity genomes, respectively, while the Puerto Rican family was chosen to represent an example of population admixture. The parents of each trio had been previously sequenced as part of the 1000 Genomes Project Phase 3 (1KG-P3)^11^ and the children from each trio have been selected for the development of new human reference genomes^7^. As a result, extensive genomic resources, such as SNV and SV callsets, single-nucleotide polymorphism (SNP) microarray data, sequence data and fosmid/bacterial artificial chromosome (BAC) libraries, have been developed to establish these trios as gold standards for SV assessment. We focused primarily on the three children for SV discovery using parental material to assess transmission and confirm phase.

We developed a multi-scale mapping and sequencing strategy using a variety of technologies to detect sequence variation of different types and sizes. To maximize sensitivity, we sequenced each child’s genome to a combined coverage of 223-fold (physical coverage of 582-fold) (Supplementary Data 1), using various short- and long-read technologies (Table 1). We discovered SVs using Illumina (IL) short-read whole-genome sequencing (WGS), 3.5 kb and 7.5 kb jumping libraries, long-read sequencing using PacBio® (PB) (Menlo Park, CA) and optical mapping with Bionano Genomics (BNG) (La Jolla, CA). We also applied a series of genomic technologies capable of obtaining long-range phasing and haplotype structure: 10X Chromium (CHRO) (Pleasanton, CA), Illumina synthetic long reads (IL-SLR a.k.a. Moleculo), Hi-C^12^, and single-cell/single-strand genome sequencing (Strand-seq)^13^ technologies (Table 1; Supplementary Data 1; Supplementary Methods 1.1).

**Table 1:** Summary of sequencing statistics

	Avg. seq. coverage	Avg. frag. length	Physical coverage
Pacific Biosciences	39.6 (child) 20.03 (parent)	8165 (child) 9619 (parent)	39.6
Oxford Nanopore	18.9 (HG00733)	11,993	18.9
Illumina short insert	74.5	694	171
Illumina liWGS	3	3475	159
Illumina 7 kb JMP	1.1	6973.2	39.2
10X Chromium	82.4	90,098	53.9
Bionano Genomics	N/A	2.81E + 05	116.7
Tru-Seq SLR	3.47	4900	3.47
Strand-seq	N/A	N/A	5.87
Hi-C	19.49	1.03E + 07	N/A
Total	223.56		607.08

Physical coverage is given for Illumina short insert, liWGS, 7 kb JMP. 10X Chromium physical coverage is estimated read cloud coverage

For Hi-C, fragment length is the distance between two read ends for intra-chromosome read pairs

### Chromosomal-level phasing and assembly of genomes

**Fig. 1:**
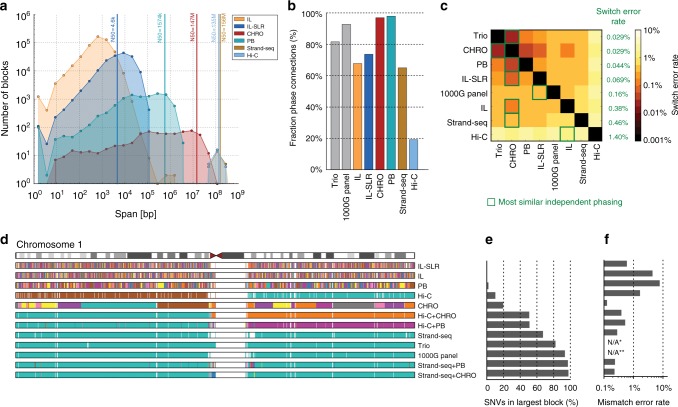
Characteristics of SNV-based haplotypes obtained from different data sources. **a** Distribution of phased block lengths for the YRI child NA19240. Note that Strand-seq haplotypes span whole chromosomes and therefore one block per chromosome is shown. Vertical bars highlight N50 haplotype length: the minimum length haplotype block at which at least half of the phased bases are contained. For Illumina (IL) paired-end data, phased blocks cover <50% of the genome and hence the N50 cannot be computed. **b** Fraction of phase connection, i.e., pairs of consecutive heterozygous variants provided by each technology (averaged over all proband samples). **c** Pairwise comparisons of different phasings; colors encode switch error rates (averaged over all proband samples). For each row, a green box indicates the phasing of an independent technology with best agreement, with corresponding switch error rates given in green. **d** Each phased block is shown in a different color. The largest block is shown in cyan, i.e., all cyan regions belong to one block, even though interspaced by white areas (genomic regions where no variants are phased) or disconnected small blocks (different colors). **e** Fraction of heterozygous SNVs in the largest block shown in **d**. **f** Mismatch error rate of largest block compared to trio-based phasing, averaged over all chromosomes of all proband genomes (i.e., the empirical probability that any two heterozygous variants on a chromosome are phased correctly with respect to each other, in contrast to the switch error rate, which relays the probability that any two adjacent heterozygous variants are phased correctly). (*) Not available because trio phasing is used as reference for comparisons. (**) Not shown as population-based phasing does not output block boundaries; refer to Supplementary Material for an illustration of errors in population-based phasing

Assembly-based SV discoveries are usually represented as a single haplotype, rather than differentiating the two haplotypes of a diploid cell. This leads to reduced sensitivity for SV detection^14^. We therefore aimed to resolve both haplotypes for the three children in this study by partitioning reads by haplotype and thereby detecting SVs in a haploid-specific manner. We applied WhatsHap^15,16^ to IL paired-end, IL-SLR, and PB reads; StrandPhaseR^17,18^ to Strand-seq data, and LongRanger^19^ to CHRO data and compared them to more traditional trio-based^15^ and population-based^20^ phasing methods. As expected, the observed phased block lengths (Fig. 1a) and marker densities (Fig. 1b) differed substantially among the platforms but the amount of phasing inconsistencies, as measured by switch error rates (Fig. 1c), was found to be very low (from 0.029% for CHRO to 1.4% for Hi-C). Since no single technology alone achieved the density, accuracy, and chromosome-spanning haplotyping necessary to comprehensively identify and assemble SVs throughout the entire human genome^17,21,22^ we systematically evaluated the performance of all possible combinations of technologies. When combining a dense, yet local, technology (such as PB or CHRO) with a chromosome-scale, yet sparse, technology (such as Hi-C or Strand-seq), we obtained dense and global haplotype blocks (Fig. 1d, e). To verify the correctness of chromosome-spanning haplotypes, we computed the mismatch error rates between the largest block delivered by each combination of technologies and the trio-based phasing (Fig. 1f). The combination of Strand-seq and CHRO data showed the lowest mismatch error rate (0.23%), while phasing 96.5% of all heterozygous SNVs as part of the largest, chromosome-spanning haplotype block (Supplementary Data 2). We note that the switch error and mismatch rates are not constant for a given technology and can be influenced by factors such as sequencing coverage, data processing, and choice of restriction enzyme in the case of Hi-C.

Once chromosomal-level phasing was obtained for each child’s genome, we partitioned the PB reads according to haplotype. On average, 68% of reads could be haplotype-partitioned in each child (Supplementary Data 3). We then developed two complementary algorithms to assemble the haplotype-partitioned reads: an extension to the SMRT-SV method^14^ (Phased-SV, Methods: Phased-SV), which produced a separate assembly for each haplotype, and an extension to an assembly algorithm^23^ (MS-PAC), which combined separate haplotype-specific assemblies with de novo assemblies in autozygous regions (Methods: MS-PAC). The assemblies covered, on average, 92.3% of the euchromatic genome (Supplementary Data 4) and produced contig N50 lengths ranging between 1.29 and 6.94 Mb (Supplementary Data 5). We then generated a high-quality consensus sequence for both assembled haplotypes^24^ from which indels and SVs could be systematically discovered by mapping the contigs to the human reference.

In addition to providing a physical framework for phasing of all genetic variants, the parent–child trio data also allowed us to detect and refine meiotic breakpoints. Using Strand-seq data, meiotic breakpoints could be determined to a median resolution of less than 25 kb (Supplementary Methods 2.1, 2.2), which was further refined to ~1.5 kb (Supplementary Data 6) by the application of trio-aware phasing from PB reads^25^. As expected, we observed an excess of maternal meiotic recombination events (Supplementary Methods 2.3)^26–29^. Further analysis of fine-mapped meiotic breakpoints support previously published results^30^ of significant enrichment for L2 elements (*p* = 0.003). Similar enrichment was found for Alu retrotransposons (*p* = 0.003), especially the AluS class (*p* = 0.001) given the mere presence of these elements within the breakpoints (Supplementary Methods 6.3). In addition, we identified an enrichment of a 15-mer motif at the breakpoints similar to previous studies^30^.

### Indel discovery (1–49 bp)

**Fig. 2:**
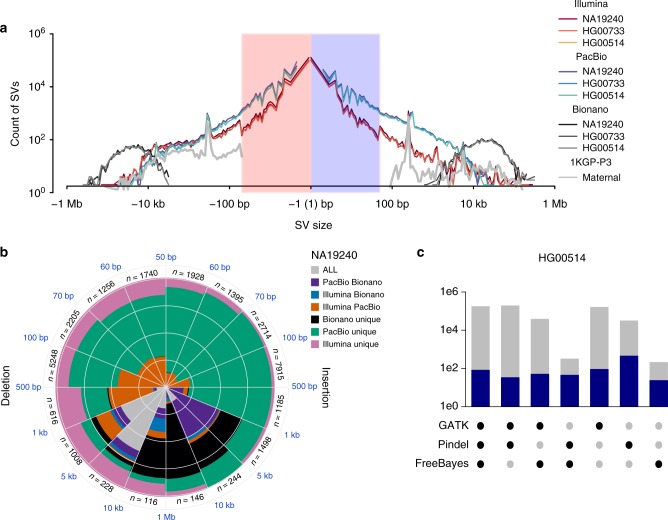
Comparison and integration of indel and SV callsets on HG00733, HG00514, and NA12940. **a** Length distribution of deletions and insertions identified by PB (blue), IL (red) and BNG (brown), respectively, together with averaged length distribution of SVs discovered in the maternal genomes by the 1KG-P3 report (silver). **b** Number of SVs discovered by one or multiple genome platforms in the YRI child NA19240. **c** Overlap of IL indel discovery algorithms, with total number of indels found by each combination of IL algorithms (gray) and those that overlapped with a PB indel (blue) in the CHS child HG00514

We generated a multi-platform indel callset by merging the IL- and PB-based callsets. Indels were detected in the IL WGS reads using GATK^31^, FreeBayes^32^ and Pindel yielding, on average, 698,907 indel variants per child (Supplementary Methods 3.1). The Phased-SV assembly alignments were used to detect indels >1 bp from the PB data yielding 345,281 indels per genome. The IL- and PB-based indel callsets showed similar size-spectrum distributions (Fig. 2a) and were merged to yield, on average, 818,054 indels per individual. The unified indel callset showed the predictable 2 bp periodicity (Fig. 2a; Supplementary Data 7) owing to the hypermutability of dinucleotide short tandem repeats (STRs)^33^. The PB reads alone miss substantial numbers of IL-based indel calls (Fig. 2c) and also lack the ability to reliably detect 1 bp indels, which are not included in the unified indel callset. However, more PB indels were discovered for variants >15 bp (12% and 23% additional for insertions and deletions, respectively) (Supplementary Data 7). We were able to confirm 89% (529/594) of the homozygous sites (45% of all sites) that overlap with ~7 Mb of BACs from the children sequenced and assembled using high-coverage (>×400) PB reads.

### SV discovery (≥50 bp)

We obtained a unified SV callset for each child from high-coverage IL WGS data, PB reads, and BNG assembly maps. To detect SVs in the IL data, we independently applied 13 SV detection algorithms (Supplementary Data 8), which included methods to capture paired-end, split-read, and read-depth information (Methods: Illumina-based SV detection, Supplementary Methods 3.3). Unlike the previous 1KG-P3 study, we sought to maximize discovery and did not strictly control for a given false discovery rate (FDR), opting to filter calls using orthogonal data in later steps. These calls were integrated into a unified Illumina-SV (IL-SV) callset (Methods: Unified Illumina SV Callset) resulting in an average of 10,884 SVs per child (comprising of 6965 deletions, 2654 insertions, 814 duplications and 451 other SVs) and 20,395 non-redundant IL-SVs across all three children (Fig. 2b). Approximately half (48.7%) of these IL-SV calls were annotated as high-confidence calls from a single algorithm, emphasizing the value of integrating multiple approaches from short-read sequencing data.

We generated a second set of SVs for each trio using the haplotype-resolved Phased-SV and MS-PAC assemblies generated from the PB long-read sequencing data (Methods: Unified PacBio SV callset). Each assembly was mapped to GRCh38, and SVs were classified as insertions, deletions, and inversions. After applying a read-based consistency check (Methods: Unified PacBio SV callset) to remove assembly and alignment artifacts, the SVs from each assembly were merged into a per-individual unified callset (PB-SV). Excluding inversions, the integrated PB-SV callset consisted of an average of 24,825 PB-SVs per child (9488 deletions and 15,337 insertions) for a total of 48,635 non-redundant PB-SVs across the three children (comprising 18,674 deletions and 29,961 insertions). The increase in sensitivity (threefold) from the PB-SV callset relative to the IL-SV callset was predominantly derived from improved detection of repeat-associated SV classes, particularly of intermediate-sized SVs (50 bp to 2 kb), and improved sequence resolution of insertions across the SV size spectrum. We note that the total SV count is dependent on the particular algorithm and gap penalties used because many of the SV calls (59% insertion, 43% deletion) map to tandem repeats where degenerate representative alignments are possible. For example, application of the double-affine gap penalty method NGM-LR^34^ reduces the number of calls by 8.8% after similar call filtration and haplotype merging. More complex evolutionary models are necessary to determine the most biologically appropriate alignment parameters.

We validated the PB-SV callset genome-wide using three different approaches. First, we searched for evidence of each child’s SVs in the long-read sequencing data of one of the two parents. We determined that 93% of the homozygous SVs and 96% of the heterozygous SVs showed read support from one of the two parents consistent with SV transmission. Second, we genotyped the insertions and deletions against IL WGS data and found that, on average, of 91.7% (21,888) of Phased-SV calls could be genotyped. Finally, we applied an orthogonal long-read technology, using Oxford Nanopore (ONT) to generate 19-fold sequencing coverage on the same PUR child genome with average read lengths of 12 kb. We also looked for support from individual ONT reads to validate the PB-SV calls. Requiring at least three ONT reads to support a PB-SV event, we achieved a 91% validation rate for SVs outside of tandem repeats and an 83% validation rate for SVs within tandem repeats (Supplementary Methods 3.2).

A substantial fraction of human genetic variation occurs in regions of segmental duplication^35^, which are often missing from de novo assemblies^36^. We compared the variation detected in regions of segmental duplication through read-depth to the segmental duplications resolved in the Phased-SV and MS-PAC de novo assemblies. The haplotype-specific de novo assemblies overlapped 24.9% (43.6 Mb/175.4 Mb) of known human segmental duplications. The dCGH and Genome STRiP methods detect variation through changes in read-depth and are sensitive to copy number changes in highly duplicated regions. We determined that 57% and 15% of the copy number variable bases within segmental duplications detected by dCGH and Genome STRiP, respectively, were not in contigs resolved by de novo assembly (Supplementary Methods 4.1). We also estimated that, on average, ~45 genes per child had at least one exon affected by a copy number change that was not detected in the de novo assemblies, highlighting the importance of continued read-depth-based CNV detection even when PB long-read-based de novo assemblies are generated.

### Characterization of inversions

**Fig. 3:**
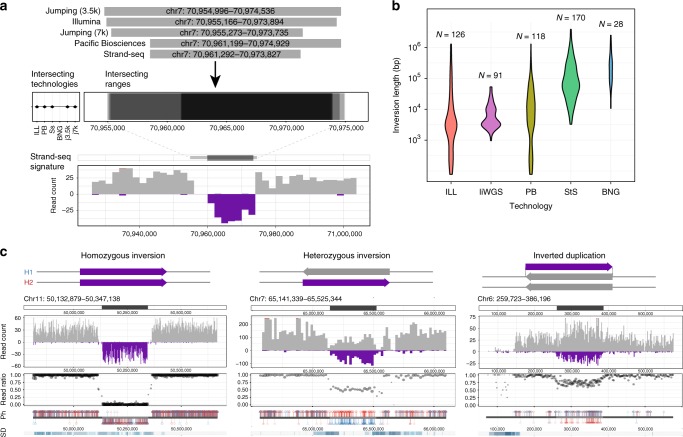
Characterization of simple and complex inversions. **a** Integration of inversions across platforms based on reciprocal overlap. Shown is an example of five orthogonal platforms intersecting at a homozygous inversion, with breakpoint ranges and supporting Strand-seq signature illustrated in bottom panels. **b** Size distribution of inversions included in the unified inversion list, subdivided by technology, with the total inversions (N) contributed by each listed. **c** Classification of Strand-seq inversions based on orthogonal phase support. Illustrative examples of simple (homozygous and heterozygous) and complex (inverted duplication) events are shown. Strand-seq inversions were identified based on read directionality (read count; reference reads in gray, inverted reads in purple), the relative ratio of reference to inverted reads within the locus (read ratio), and the haplotype structure of the inversion, with phased read data considered in terms of directionality (Ph; H1 alleles in red, H2 alleles in blue; alleles from reference reads are displayed above the ideogram and alleles from inverted reads are displayed below). ILL Illumina. liWGS long-insert whole-genome sequencing libraries. PB Pacific Biosciences. StS Strand-seq. BNG Bionano Genomics. SD segmental duplication. Ph phase data

Inversion variation has long been the focus of cytogenetic studies using karyotyping, and more recently, these large, rare inversions have been characterized at sequence resolution^37,38^. However, submicroscopic and polymorphic inversions are ill-defined by human genome sequencing, in part, because larger events tend to be flanked by virtually identical duplicated sequences that can exceed a million base pairs in length that cannot be bridged by short-read sequencing technology^2^. Moreover, the copy-neutral nature of simple inversions precludes detection by read-depth analysis. To generate a map of inversions across different length scales, we called inversions with five complementary techniques, including IL WGS, long-insert whole-genome sequencing (liWGS), PB, BNG optical mapping, and Strand-seq (Methods: Inversion Detection Using Strand-seq). For Strand-seq, we developed a computational algorithm integrating inversion discovery with trio-aware phasing data to bolster accuracy and only retained those calls that displayed haplotype support (Methods: Classifying Strand-seq Inversions Using Orthogonal Phase Data). A careful comparison of inversion calls revealed that Strand-seq was the only platform that made highly reliable calls on its own, while for the other technologies acceptable accuracy was achieved only for calls supported by at least two platforms (Fig. 3a; Methods: Integrating Inversion Calls across Orthogonal Platforms). The unified, high-confidence inversion callset comprised 308 inversions across the nine individuals, corresponding to an average of 156 inversions (~23 Mb) per genome. Of these 308, 74% were either primarily identified by Strand-seq (*n* = 170) or received additional Strand-seq genotype support (*n* = 59). By comparison, 126 inversions in the unified callset were detected by IL WGS, 118 in PB, 91 in liWGS, and 28 in the BNG data.

The inversion size spectrum differed markedly among platforms (Fig. 3b). IL WGS, PB, and liWGS excelled in mapping relatively small inversions (<50 kb), wherever breakpoint junctions could be traversed by DNA sequence reads. Indeed, the smallest inversions (<2 kb) were only detected by IL WGS and PB. In contrast, larger inversions (>50 kb) were nearly exclusively detected by Strand-seq. The Strand-seq technique offers the advantage of inversion detection solely by identifying DNA sequence strand switches internal to the inverted sequence, readily identifying inversions flanked by large segmental duplications that can be neither assembled nor traversed using standard DNA sequencing technologies^39^. Inversions called by Strand-seq show a median size of 70 kb (up to 3.9 Mb in length), in sharp contrast to IL-detected events, whose median size is 3 kb (down to 263 bp in length) (Supplementary Data 9).

Within the unified inversion callset, 73.7% (227/308) represent copy-neutral (i.e., simple) events, whereas 79 are more complex inversions containing embedded copy number variation (most in the form of inverted duplications). Consistent with previous observations that inversions map within segmental duplications, 50.7% of the inversions have both breakpoints mapping within segmental duplications (115/227)—an eightfold increase when compared to unique regions of the genome. Furthermore, inversions within segmental duplications are ~20-fold larger, with a median length of 72.2 kb compared to a median length of 3.4 kb for inversions with breakpoints outside of segmental duplications. On average, each individual genome harbors 121 simple and 35 copy-variable inversions, approximately 2/3 (66.8%) are heterozygous and 1/3 (32.5%) are homozygous. Chromosomes 16 (5.2%), 7 (3.4%), X (3.3%), and 8 (3.0%) show the highest frequency of inversions, consistent with prior expectation^4,7,39^. The inverted duplications typically exhibit highly variable copy number states, ranging between 0 and 10 (mean = 4) copies (Supplementary Data 10), indicating a large source of genetic variability between individuals. For instance, a 260 kb complex inversion mapping to chromosome 9 (at ~40.8–41.1 Mb) contains between 4 and 6 copies in each genome. Another notable example is an inverted duplication at the *DUSP22* locus (Fig. 3c), for which a copy was known to be missing from the human reference^40^; we show it to be in the reverse orientation. Additionally, 40 inversions were found to be homozygous in all nine individuals and likely reflect minor alleles or remaining assembly errors in the human reference (Supplementary Data 11; Supplementary Methods 5).

Inversion polymorphisms at several loci (such as 3q29, 7q11.23, 8p23, 15q13.3, 15q24, 17q12, and 17q21.31) were previously reported to predispose parental carriers to children with microdeletion and microduplication syndromes associated with developmental disorders^41–43^. The substantially increased number of inversions generated in our study prompted us to investigate whether this association holds for other microdeletion and microduplication syndromes. Twenty-one events from our unified inversion callset displayed >80% overlap with one of the 255 critical regions^4,41^ associated with genomic disorders (Supplementary Data 12). An additional 37 inversions were partially overlapping one of the 255 critical regions. Interestingly, in 12 cases we found novel inversions at both boundaries of the respective critical regions, including the 16p13.1 and 16p11.2-12 critical regions on Chromosome 16p (Supplementary Figure 1). A 1.9 Mb inversion at chromosome 3q29, for example, was previously shown to predispose to pathogenic SVs^42^, and we identified a smaller 300 kb inversion intersecting the proximal breakpoint of this critical region. We hypothesize that the inversion polymorphisms we have identified will alter the orientation of low copy repeat sequences (Supplementary Figure 1, right panel), and as such may differentially predispose individual loci to undergo pathogenic deletion or duplication via non-allelic homologous recombination.

### Mobile element insertions

Previous SV studies have been unable to resolve the sequences of large mobile elements in the human genome limiting our ability to assess differences in mutagenic potential between individual genomes. However, since PB long reads were routinely larger than 10 kb in length, we used the PB-SV callset to investigate not only the location but the sequence content of full-length L1 (FL-L1) elements. We detected an average of 190 FL-L1 elements with two intact open reading frames in the three children (Supplementary Figure 2; Supplementary Methods 6.3). Only 56 of these copies are shared across the three genomes (Supplementary Data 13). This diversity in mobile element profiles likely influences L1 mutagenic potential. For example, while all three of the genomes are homozygous for one of the most active retrotransposon source L1 elements associated with human cancers (chr22:28663283^30,43^) and another L1 is highly active (i.e., hot) in the germline and cancers, each genome also harbors two to six unique hot L1 source elements. One of the unique hot L1 copies in the PUR individual is the *LRE3* element, which is the most active L1 source element in humans^44,45^. Twenty-eight FL-L1 copies with low-to-moderate levels of activity are also differentially present in the genomes of the three individuals. The cumulative differences in L1 mutagenesis that emerge from these diverse FL-L1 profiles suggest that, at a population level, such diversity may translate into differential risk levels for L1-mediated diseases, such as cancers and other disorders^43,46^.

### Genotyping novel SVs in population cohorts

One of the advantages of having a more comprehensive set of sequence-resolved SVs is the ability to accurately genotype them in different human populations. We genotyped SV calls from the base-pair-resolved PB-SV callset in a limited set of 27 high-coverage genomes using a sensitive, but computationally intensive method, SMRT-SV Genotyper^14^. An average of 91.7% (21,888) of Phased-SV calls could be genotyped with this approach across both insertions and deletions (Supplementary Methods 6.1), with average Mendelian error rates of 14.1% for insertions and 8.7% for deletions.

### Functional impact with respect to gene structure

An important consideration of increased sensitivity afforded by this multi-platform algorithm is its potential functional impact (Supplementary Methods 6.2). Although the number of individuals compared is few, we sought to determine the number of genes that would be modified or disrupted based on the unified callset. On average, each child has 417 genes with indels and 186 genes with SVs predicted to affect protein-coding sequence. The coding frame is preserved for the majority of the genes with exonic indels (78.2%, 326/417) or SVs (30.1% (56/186) of intersecting genes (Supplementary Datas 14, 15). For frameshift events, we considered the intolerance to loss-of-function mutation as measured by pLI^47^, which ranks genes from most tolerant (0) to least tolerant (1) of mutation. The median percentile of frameshift indels was 0.002 and SV was 0.155, indicating most of the loss-of-function variation is of modest effect (Supplementary Data 16). There were only two genes with high pLI (>0.90) that showed deletions concordant with the IL and PB data: *RABL1* (HG00514) and *ACTN1* (NA19240), and one 34 bp deletion leading to an alternate splice-site in *TSC2* in HG00733. We also identified 16 genes (such as *HTT*) that were intolerant to loss-of-function (pLI > 0.9) but carried in-frame indels—a potential source of triplet instability. A total of 674 known canonical genes overlap inversions (Supplementary Data 17), of which 88% have isoforms entirely contained within the inversion, and 6% are intronic. The remaining events are potentially gene disrupting, where three events overlap at least one exon of genes (*AQPEP*, *PTPRF*, and *TSPAN8*). Up to 55 genes have at least one isoform that spans one of the breakpoints of the inversion; however, the majority (95%) of these genes reside in segmental duplications where the exact breakpoint of the inversion cannot be easily resolved.

Variation in untranslated region (UTR) sequences may also affect gene expression leading to phenotypic consequences. We overlaid our SV dataset with UTRs and found that each child carried on average 155 genes with a deletion and 119 genes with an insertion in a UTR. Such genes, however, tended to be more intolerant to variation compared to exonic deletions, with a median pLI of 0.20. For example, there were 23 genes with UTR insertions or deletions with PLI scores >0.9: *ATP11A*, *BANP*, *BRWD3*, *DGKD*, *EIF4A3*, *FAM135B*, *FURIN*, *HCN1*, *IQSEC3*, *MEGF10*, *NIPBL*, *PAXBP1*, *PPP2R5E*, *SHB*, *SLC38A2*, *SNED1*, *SON*, *SREK1*, *TDRD5*, *TMEM165*, *XIAP*, *XPR1*, and *ZNF605*. The mean length of these UTR deletion variants was 176 bp and is similarly reflected in the technology bias for sensitivity; only one event was detected by BNG, nine by IL-SV, and 19 by PB-SV.

We also considered the overlap of SVs with functional noncoding DNA (fnDNA; Supplementary Methods 4.2): specifically with 1.07 M transcription factor binding sites (TFBS) and 2.86 M conserved elements (CEs). Deletion variants overlapped an average of 861 and 1767 TFBS and 3861 CEs in each child. However, small SVs rarely affected fnDNA: the average size of SVs that overlapped TFBS and CEs were 36,886 bp and 15,839 bp, respectively. When considering the IL-SV and PB-SV callsets, the majority of TFBS and CE deletions are detectable in the IL-SV callset (89.1% and 77.4%, respectively). Nevertheless, we estimate that 21 TFBS and 181 CEs would be missed per child by application of short-read sequencing technology alone. The opposite pattern exists for insertion SVs in fnDNA. While a smaller number of insertion SVs map inside TFBS (an average of nine per child, with average SV length of 665 bp) and 154 insertion SVs map inside a CE (average length of 930 bp), they were predominantly detected in the PB-SV callset; 57% of TFBS and 69% of CEs affected by SVs were detected only in the PB-SV callset compared to 7% and 5%, respectively, for IL-SV. Variants with imprecise insertion breakpoints, such as the BNG calls, were not considered. Thus, the application of multiple technologies enables additional resolution of smaller fnDNA SV.

### Platform comparisons and optimal indel and SV detection

The use of orthogonal technologies and various discovery algorithms on the same DNA samples provide an opportunity for a systematic assessment of the performance of individual as well as combinations of algorithms for indel and SV detection. While long-read technology generally outperforms IL-based algorithms for indel detection by ~50% for indels ≥15 bp, it is not reliable for single-based indels even at 40-fold sequence coverage, particularly in homopolymer regions. Benchmarking against the unified-indel dataset, we find that maximum sensitivity for IL indels requires application of three callers, including GATK, FreeBayes and Pindel (the latter of which has a higher false positive rate). Current large-scale studies rely mainly on IL sequencing, and computational resources limit the number of algorithms that can be applied to a genome. We therefore used a pan-SV callset (union of IL-SV, PB-SV, and BNG) to gauge the sensitivity and specificity of individual and combinations of IL-only algorithms. To construct the pan-SV callset, IL-SV insertions/deletions and BNG deletion calls were filtered according to orthogonal support datasets (formed from orthogonal callsets, raw PB reads, unfiltered PB-SV calls, and read-depth information). On average, 83% of IL, 93% of PB, and 95% of BNG deletion calls, and 82% of IL and 96% of PB insertion calls had orthogonal support. The concordant IL-SV and BNG calls were merged with the entire PB-SV callset to form the pan-SV callset. The unified callsets contained an average of 11,106 deletion and 16,386 insertion calls per individual (Table 2). As expected, the PB-SV callset provided the most unique calls, including 47% of deletions and 78% of insertions, which were primarily driven by tandem repeat variation (75%) and mobile element insertions (6.3%).

**Table 2:** Unified technology callset for copy number gain and loss structural variation

	HG00514		HG00733		NA19240	
	Count	Average length	Count	Average length	Count	Average length
*Deletions*						
PacBio	4662	195.7	5074	193.1	5586	205.0
Illumina	1387	792.0	1251	563.1	1760	664.9
Bionano	109	11,7901.6	88	86,440.9	113	115,099.1
PacBio,Illumina	3403	298.0	3482	294.2	4132	308.3
PacBio,Bionano	128	7569.5	128	4523.8	119	4633.7
Illumina,Bionano	50	8516.0	42	9680.3	54	9767.3
All	552	3997.0	542	4076.8	657	3647.4
Total	10,291	1892.6	10,607	1273.7	12,421	1615.9
*Insertions*						
PacBio	11,314	294.2	12,272	302.7	12,080	285.0
Illumina	533	501.4	483	1610.6	663	2163.7
Bionano	473	21,452.6	418	18,346.5	497	16,700.6
PacBio,Illumina	2146	239.5	2236	262.5	2631	260.6
PacBio,Bionano	984	2539.3	1052	2510.8	1035	2501.2
Illumina,Bionano	33	14,733.4	22	5905.0	26	6541.4
All	83	3500.9	83	3808.6	94	3751.8
Total	15,566	1126.3	16,566	955.9	17,026	997.0

**Fig. 4:**
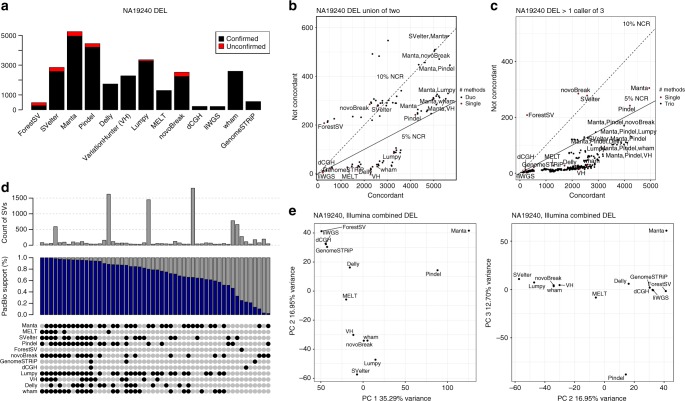
Concordance of IL methods compared against total IL callset and PB callset using orthogonal technologies. Results by algorithm shown for **a** the deletion concordance for individual methods, **b** the union of all pairs of methods, and **c** the requirement that more than one caller agree on any call. Individual callers are shown as red points for comparison. Pairs and triples of combinations are in black points. The solid and dashed lines represent the 5% and 10% non-concordance rates (NCR), respectively. The top five combinations of methods in each plot below the 10% NCR, along with the individual plots, are labeled. **d** Overlap of IL-SV discovery algorithms, with total number of SVs found by each combination of IL algorithms (gray) and those that overlapped with the PB-SV calls (blue) in the YRI child NA19240. **e** PCA of the genotypes of concordant calls of each method: PC 1 versus 2 (left), PC 2 versus 3 (right). VH VariationHunter

Across the entire IL-SV dataset, the deletion concordance to pan-SV was 82.9% (largely unaffected by size), whereas the insertion concordance to the pan-SV callset was 82.0%, decreasing in sensitivity with increased insertion SV length (Supplementary Data 18). The BNG mean concordance rate for deletions was 95.2%. When considering individual methods, the average concordance for deletion calls ranged from 46.4% to 99.3% with a median of 94.7% (Fig. 4a), and for insertion calls ranged from <1% to 97% (Methods: Integration of Illumina, PacBio, and Bionano Callsets). When compared to the pan-SV callset, the concordant calls from individual algorithms detected 1.7%–40.7% of deletion and <1%–7.9% of insertion SVs.

It has been shown previously that sensitivity for true SV calls (generated from IL datasets) can be improved by combining calls from more than one algorithm^10,48–50^. Because it would be computationally burdensome for a large-scale WGS study to run all available algorithms, we used the integrated callset to compare how SV-calling performs under different combinations of algorithms (up to three) on standard IL data (i.e., without liWGS), testing both unions and intersections of callsets. We used the non-concordance rate (NCR) (1—concordance) as a proxy for FDR. Considering the YRI child, NA19240, there were 29 combinations representing unions of two methods with an NCR less than 5% (Methods: Integration of Illumina, PacBio, and Bionano Callsets). For example, the union of the Pindel and VariationHunter callsets produced 4892 calls at an NCR of 4.8%. Greater specificity may be obtained by requiring two of three methods to agree. There were 213 combinations of callsets with an NCR less than 2%, but the maximum sensitivity came from the combination of Manta, VariationHunter, and Lumpy (3182 calls at an NCR of 2.9%) including 13% of deletions from the pan-SV callset (including 65% of mobile element insertions and 4.5% of simple tandem repeats and variable-number tandem repeat deletions) (Fig. 4b, c). The sensitivity of insertion-calling algorithms was driven by methods that detect mobile element insertions, particularly the MELT algorithm. We observed that while no single SV was called by every algorithm tested, there are often sets of algorithms that call similar variants (Fig. 4d, e), and these principal component analyses (PCAs) may provide a conceptual framework for optimizing SV detection and computational burden in future studies.

## Discussion

This study represents the most comprehensive assessment of SVs in human genomes to date. We employ multiple state-of-the-art sequencing technologies and methods to capture the full spectrum of genetic variation down to the single-nucleotide level, in a haplotype-aware manner. Our results indicate that with current methods, using multiple algorithms and data types maximizes SV discovery. The PB, Strand-seq, and CHRO data were combined to generate haplotype-resolved de novo assemblies constructed from phased PB reads. When paired with high-coverage IL sequencing and BNG SVs, we discovered approximately sevenfold more variation than current high-coverage IL-only WGS datasets^4^, which is, on average, 818,054 indels (1–49 bp) and 27,622 SVs, including 156 inversions per person. Consistent with increased genetic diversity among African populations^11^, we observed 20.7% more deletion and 9.4% more insertion variants in the Yoruban child than the Han Chinese child.

The long-read sequence data provided us with an unprecedented view of genetic variation in the human genome. Using average 5–10 kb reads at an average of 40-fold sequence coverage per child, we have now been able to span areas of the genome that were previously opaque and discover 2.48-fold more SVs than the maximum sensitivity achieved by integrating multiple algorithms for SV detection in short reads. Our analysis suggests that the majority (~83%) of insertions are being missed by routine short-read-calling algorithms. Specifically, the largest gain stems from tandem repeat and retro-transposon insertions in the 50 bp to 2 kb size range. Inversions represent another problematic class of human genetic variation. In 1KG-P3, 786 inversions were identified across 2504 genomes representing 3.3 Mb of sequence^4^, and in the current study, we identified 308 inversions from just three family trios, totaling 36.4 Mb of sequence. This increase in sensitivity depended on the complementary nature of the five different technologies (Fig. 3b). In the shorter size range, inversion discovery largely depended on a combination of IL and PB datasets, whereas for the larger events, Strand-seq was required. As a result, we were able to identify 181 inversions that were missed as part of 1KG-P3. Most of these are large (>50 kb) constituting an average of 156 inversions and representing 22.9 Mb of inverted DNA per diploid genome, which corresponds to an ~480-fold increase in inverted bases per individual when compared to the 1000 Genomes Project^4^. Our results indicate that for maximum sensitivity and specificity related to SV discovery it is essential to employ more than one detection algorithm and more than one orthogonal technology. This allowed us to locate new inversions to the boundaries of critical regions implicated in microdeletion and microduplication syndromes. Inversion polymorphisms have already been linked to disease, including Koolen-de Vries, Williams-Beuren, and 17q21 and 15q13.3 microdeletion syndromes^42,51,52^. Here we nominate additional inversions that may similarly predispose critical regions to undergoing deletion or duplication.

It is not practical for large-scale studies to detect variation by employing the menagerie of sequencing methods and algorithms used in this study. Instead, these data serve as a guide for the trade-off between the cost of sequencing and desired sensitivity for SV detection. For example, we demonstrated entire chromosomal phasing using the Strand-seq and CHRO libraries; however, the Strand-seq method is not yet as widely implemented in sequencing facilities as Hi-C, which when combined with CHRO libraries provides chromosome-arm level phasing and is likely sufficient for many applications. Similarly, with the high-coverage IL sequencing and the many algorithms used here, it was possible to detect up to ~52% of the total number of deletion SVs and ~18% of insertion SVs. Moreover, we performed a series of down-sampling experiments to determine the equivalence of our analyses to datasets routinely used for large-scale studies (IL 30X coverage) and execution by non-expert users (i.e., by using default parameters) for SV detection (Supplementary Methods 3.3). These analyses revealed just an 11% reduction in calls attributable to the lower 30X coverage, but a 23% reduction in calls using default parameters from the six algorithms with greatest contribution to our final callset. Collectively, these analyses suggest that if large-scale studies such as TOPMed (https://www.nhlbi.nih.gov) or CCDG (https://www.genome.gov) were to rely on an individual algorithm, we estimate the sensitivity to detect deletion SVs outside duplicated portions of the genome would be at most 40% with an FDR of ~7.6% (using Manta). Insertion sensitivity would fare far worse with an estimate of ~7% and an FDR of 4%, but only for mobile element insertions using MELT. While the majority of the variants associated with coding regions missed by IL-based analysis appear to be neutral in effect, there is a threefold increase of SVs detected in coding sequences for specific genes (albeit genes more tolerant to mutation) when including the PB-SV callset. Importantly, the addition of the PB-SV callset increases sensitivity for genetic variation, which could have a more subtle effect on gene expression changes, including a twofold increase in variation in UTR sequences and a ~20% increase of SVs detected in TFBS and CEs.

Our analyses indicate that the contribution of SVs to human disease has not been comprehensively quantified based upon studies that have relied upon short-read sequencing. Until the cost and throughput of long-read sequencing support larger-scale studies, we propose that future disease studies consider a triaged application of multiple technologies to comprehensively identify SVs. Families that have been sequenced using IL-based WGS should be analyzed using intersections of multiple SV-calling algorithms (e.g., Manta, Pindel, and Lumpy for deletion detection, and Manta and MELT for insertion detection) to gain a ~3% increase in sensitivity over individual methods while decreasing FDR from 7% to 3%. Because a disproportionate number of bases affected by variation occurs in segmental duplications and PB-based assembly does not resolve the segmental duplication regions entirely, there is a need to apply other algorithms, such as read-depth methods (e.g., dCGH or Genome STRiP), to detect changes in copy number in highly duplicated regions of the genome. The sequence structure of such variation is still not resolved and novel methods will need to be developed to sequence-resolve CNVs^53^. Of note, there is a pressing need to reduce the FDR of SV calling to below the current standard of 5% because forward validation of all potentially disease-relevant events will be impractical at this threshold. We also predict that a move forward to full-spectrum SV detection using an integrated algorithm could improve diagnostic yields in genetic testing. Moreover, the proper application of SV detection for patient care requires a deeper understanding of germline SVs from more individuals across diverse global populations.

## Methods

### PacBio-based SV detection

Existing methods for detecting SV lack sensitivity on diploid genomes^14^. To address this, we developed a strategy for variant calling where dense chromosome-scale phased SNVs are used to partition sequence reads by haplotype, and SVs are called by assembling each haplotype separately and detecting SVs as differences between the haplotype assemblies and the reference. Whole-chromosome phasing was generated using a combination of Strand-Seq and CHRO, phased by WhatsHap^16^. SNV density is sufficient to provide good contiguity. The mean distance between phased SNV sites was 1360, 1497, and 1040 bp for the CHS, PUR, and YRI children, respectively. We aimed to generate assemblies for each haplotype despite having lower sequence coverage per haplotype than previous human and great ape studies^54,55^. To do so, we generated de novo assemblies using two related assembly approaches: local de novo method Phased-SV and an unguided de novo method MS-PAC, and used concordance between assemblies to validate integrity.

### Haplotype-specific de novo assembly

Phased-SV: For the local approach, we assembled reads into 60 kb regions spaced at 20 kb intervals across the genome (148,923 regions). Reads were mapped using BLASR^56^ to GRCh38. Regions were labeled as haplotype-resolved if at least 20 heterozygous SNVs were present in the region (CHS = 90,631, PUR = 83,758, YRI = 113,972 regions) (Supplementary Figure 3). Otherwise regions of the genome were considered autozygous. In haplotype-resolved regions, reads were partitioned by haplotype from each child, and combined with reads of the parents from the corresponding inherited parental haplotype (for a total of 30-fold sequence coverage per haplotype). The autozygous regions were assembled without partitioning reads. The average fraction of partitioned reads was 60.1%, 67.0%, and 70.1% for the CHS, PUR, and YRI children, respectively. The set of local assemblies from each haplotype combined with the assemblies from autozygous regions were then merged into megabase-scale haplotype-resolved contigs. For each haplotype, a directed acyclic graph (DAG) was generated with a vertex for every local assembly from the haplotype as well as local assemblies from the autozygous regions, with edges connecting two nodes if the corresponding local assemblies were from genomic regions separated by at most 100 kb, and with overlap alignments at least 10 kb, with direction of the edge determined by the genomic order of the local assemblies. A contig was generated corresponding to the longest path in each weakly connected component of the DAG.

MS-PAC: For haplotype-partitioned de novo assembly, reads were aligned to GRCh38 using BLASR, as described above. Using whole-chromosome phasing information provided by WhatsHap, we partitioned the reads into three sets: haplotype 1 reads, haplotype 2 reads, and unassigned reads. To infer the most likely haplotype, for each read we examine all base QVs, *q*_*i*_, and incidents on SNVs. For each read we give it a simple haplotype score using the product of *q*_*i*_ for each matching base or 1-*q*_*i*_ for each based which does not match. Scores for haplotype 1 and 2 are compared; reads are assigned to the haplotype with higher score if the difference in scores exceeds a phred-scaled QV of 10. Any remaining reads, including those which do not span any SNVs, are labeled as ambiguous. The average coverage per sample decreased by 39.84% ± 1.52 of the original coverage per haplotype after partitioning.

After partitioning, each haplotype is assembled independently. For each haplotype, candidate assembly intervals were defined as those with greater than ×3 coverage. Prior to assembly each such region (10157, 8908, 12829 in NA19240, HG00733, HG00514 in haplotype 1 and 10065, 8665, 12826 in NA19240, HG00733, HG00514 in haplotype 2) was split into subintervals of 270 kb (with 10 kb overlap between adjacent intervals). For each haplotype-specific region, ambiguous reads overlapping the intervals were also recruited. The combined read set was then assembled using a step-wise approach. First, assembly was performed with Canu^57^ with parameters: contigFilter = “2 1000 1.0 1.0 2”; corMinCoverage = 0; errorRate = 0.035. Next, for regions that did not assemble, a more permissive assembly was performed using minimap and miniasm^58^ with error-corrected reads generated in the prior step by Canu. The resulting process sometimes generated multiple contigs that did not span the whole interval. In the regions with no contigs, reads were extracted and locally reassembled by extracting subinterval reads with 10 kb flanks. The resulting assemblies were quivered and trimmed for regions with Phred QV >30 using cutadapt^59^, to yield the initial set of assembled haplotigs.

For each haplotype, contigs were mapped back to the reference using BLASR and the *-alignContigs* option. Any overlapping contigs were merged by greedily extending the upstream contig to its last aligned base, i, before adding bases from the downstream contig beginning at i + 1. After creating the stitched haplotigs, a final step was performed to merge de novo assembled sequence from Falcon (https://github.com/PacificBiosciences/FALCON), described previously. Here, Falcon assembly substrings were only integrated in “gap” intervals if they anchored to both the flanking left and right haplotigs of an examined gap interval. The N50 before adding in the Falcon assembly was 277 kb, 243 kb, and 169 kb for haplotype 1 for HG00733, HG00514, and NA19240, respectively. For haplotype 2, the N50 is 277 kb, 242 kb, 171 kb; after addition of the de novo assembly the N50 contig was for haplotype 1 was 6 Mb, 3.2 Mb, 1.5 Mb and for haplotype 2 the 6.3 Mb, 3.1 Mb, and 1.4 Mb. The merged assemblies were then aligned back to the references with BLASR using the parameters (*-alignContigs -noSplitSubreads -bestn 1 -clipping soft*).

### Assembly coverage

The haplotypes from each assembly were aligned using BLASR *-alignContigs -minMapQV 30* (www.github.com/mchaisso/blasr), and intersected with chromosomal regions not labeled as acrocentric bands within the UCSC cyctoband tables http://genome.ucsc.edu/cgi-bin/hgTables?clade=mammal&org=Human&db=hg38&hgta_group=map&hgta_track=cytoBandIdeo&hgta_table=0&hgta_regionType=genome&hgta_outputType=bed. The coverage is reported in Supplementary Data 19. Assembly quality was measured by mapping BAC sequences (27 in total) to both haplotypes of each assembly and counting the difference to the optimally aligned haplotype. The result is given in Supplementary Data 20.

### Quality control for PacBio callset

SVs called by the two methods were first filtered by PacBio read alignment count (PBRC) and comparison with the Bionano Genomics (BNG) SV callset. To determine PBRC, SVs were organized into clusters where all variants within a cluster had boundaries within a specific window (length = 250 bp). The original Phased-SV callsets formed 17,141 clusters per haplotype (HG00514 = 15,450, HG00733 = 17,474, and NA19240 = 18,499) from the original set of 25,113 (HG00514 = 23,325, HG00733 = 25,754, and NA19240 = 26,260 pre-filtered calls. Support for MS-PAC calls was determined separately for each haplotype and the “remaining de novo” calls prior to merging. The haplotype callsets had between 25,741 and 28,032 original calls organized into 34,644–46,391 clusters, and the de novo calls had considerably more, between 43,271 and 60,079 original calls organized into clusters. For each SV cluster, we define the start position of the reference interval of the cluster as 1 kb upstream of the first variant. We define the end position as 1 kb downstream of the endpoint of the last SV in the cluster (if the SV was a deletion), or 1 kb downstream of the starting point of the last SV in the cluster (if this SV was an insertion). A target database of two sequences was generated for each cluster: the reference sequence extracted from the corresponding interval to the SV, and an alternative sequence containing modifications to the reference interval sequence defined by the SVs in the cluster; e.g., if the cluster contained a 100 bp deletion, this sequence would be removed from the alt-reference. All reads overlapping the first SV in the cluster were mapped to the two-sequence database, and reads were assigned to the target with according to best alignment score. We filtered out reads with alignments <1.5 kb.

To determine the minimal number of reads aligning to the alt-reference required to validate a call, we performed a simulation. The level of support for the alternate allele was measured for 22,166 variants detected on haplotype 0 of NA19240 randomly shuffled to different euchromatic regions of the genome (Supplementary Figure 4). We found that >4 reads supporting the alternate allele results in an estimated FDR of 0.21%. The average number of SVs per haplotype that had PBRC >4 for were HG00514 = 17,708, HG00733 = 19,304, NA19240 = 20,118, and the MS-PAC haplotype assembles were HG00514 = 15,439, HG00733 = 15,035, NA19240 = 17,046, with the de novo assemblies (not haplotype specific) ranging between 7044 and 10,938 calls (Supplementary Data 21).

As a final step to recover SVs with low sequence coverage, we compared the initial SMRT SV callsets to variants discovered through BNG.

BNG calls were intersected with PB calls generated by MS-PAC and Phased-SV. BNG-SV calls do not yield precise breakpoints, but instead provide reference intervals and query intervals for which the observed nick-site distance is significantly different from expectation. We calculated the BNG event size, and positive values for x indication insertions while negative values indicate deletions.

We then compared these intervals to all SV events (passing and non-passing) predicted from MS-PAC and Phased-SV. For all BP events overlapping a BNG event we define L_PB_ as the difference between the reference and alt allele. We can then score the similarity between each of the events to the BNG event as *f*_BN_ = |L_BN_–L_PB_|/L_BN_, selected the corresponding event which minimized *f*_BN_. Reporting for HG00514, HG00733, and NA19240 in order, a total of 282, 293, and 385, deletion events, and 389, 421, and 429 insertion events for Phased-SV were identified as having a best match and 276, 267, and 349 deletion events and 348, 337, and 467 insertion events for MS-PAC (Supplementary Data 22; Supplementary Figure 5) shows the concordance between the estimated PB and BNG event sizes. Note, we also examined concordance of the L_BN_ with sum of PB events (in each callset) overlapping the interval. However, this led to lower concordance then selecting the single best event.

### Unified PacBio SV callset

We then generated a unified callset combining the Phased-SV and MS-PAC SVs. We initially observed low reciprocal overlap between the two callsets. 9751/21,733 (HG00514), 10,171/23,210 (HG00733), and 12,072/25,309 (NA19240) quality-filtered MS-PAC calls had 50% overlap between calls of the respective callsets from Phased-SV. However, most calls from MS-PAC have a call in Phased-SV that was located in close proximity: 12,851/14,763 (87%), 13,640/15,363 (89%), and 16,336/19,092 (86%) have a call at most 1 kb away in Phased-SV *p* = <1e^−5^, permutation test.

We based our merging strategy on the observation that the two approaches tended to make calls that were within close proximity and of similar length between the two methods, or calls that were far apart. Additionally, we found that the standard of using the 50% reciprocal overlap to merge calls that are in close proximity was insufficient. When the two methods had similar calls at the same locus but with different breakpoints, the reciprocal overlap method would leave many calls unmerged, inflating the number of calls. The total number of MS-PAC calls per haplotype are listed along with the number of calls unique to the MS-PAC callset using a simple 50% reciprocal overlap metric compared to the number of calls unique to MS-PAC when restricted to at least 10 kb away from a Phased-SV call in Supplementary Data 23.

To first show the general agreement between the two methods, we compared the net gain of bases of SVs in 29,609 tiled 100 kb windows across the euchromatic genome. We considered SV callsets from Phased-SV and MS-PAC (haplotype and de novo merged) that have passed read-backed filtering. For each window and callset, the sizes of the SVs within the window were summed, with deletion calls as negative and insertions as positive (Supplementary Figure 6). The correlation between methods across bins where at least one method has an SV call ranges between 0.27 and 0.46 across the genomes (Supplementary Data 24), which is a lower correlation than what we would have expected. However, limiting to cases where both methods make a call and the difference between calls is at most 10 kb, the correlation is between 0.89 and 0.94, indicating that when both methods produce calls in a region the net SV is similar.

Next, for every call in the Phased-SV haplotype-specific callsets, we calculated the closest MS-PAC call, without regard to the call size. Similar to the bin-based analysis, calls were either in close proximity (<1000 bases), or were far apart (>5000 bases) (Supplementary Figure 7).

The differences between calls from MS-PAC and Phased-SV were cataloged by haplotype, separating out calls by not-tandem repeat and tandem repeat sequences. For not-tandem repeat sequences, 18% of MS-PAC calls did not have a nearby call in the same haplotype in Phased-SV; however, only 4.7% of MS-PAC calls were missing from either haplotype in Phased-SV, indicating that roughly 30% of the assembly differences were due to haplotype switching. The remaining calls (4.7%) represent either errors in the MS-PAC assembly, or assembly dropout from Phased-SV. Of these calls, 13% were not backed by raw read support (0.6% of the entire MS-PAC callset) indicating likely mis-assemblies that are filtered by quality checking. Regions missing from both haplotypes of the Phased-SV assemblies accounted for 25% of the MS-PAC calls (1.2% of entire MS-PAC callset). The remaining 3.5% of calls unique to the MS-PAC callset have PB read-backed support, and are covered by a Phased-SV assembly. Manual inspection of some of these calls indicates the most likely source of error is a missing haplotype or calls for which the read-backed filter incorrectly removed a call. The full summary of steps to merge is given in Supplementary Data 25.

The total number of SVs detected are determined by the parameters of merging SVs from both haplotypes in addition to both assembly methods. We provide greater detail on the merging of calls by haplotype and present an approach to merging SVs between haplotypes where calls inside tandem repeats are merged separately. Because our approach to detect SVs in diploid genomes is to assemble the two haplotypes separately and combine calls into a diploid genome, calls that overlap with a certain threshold are considered homozygous, and when there is less than this threshold heterozygous and unique to each haplotype. We examined the effect of the merge parameter for calls in tandem and non-tandem repeat regions for both Phased-SV and MS-PAC (Supplementary Figure 8). For calls outside of tandem repeats there is a less than 10% difference in total number of calls when the reciprocal overlap threshold ranges from 10% to 90%, indicating that the breakpoints of SV calls detected in separate haplotypes outside of tandem repeat regions are consistent. The results are consistent between Phased-SV and MS-PAC. Thus, we elected to use a loose overlap threshold (0.10) for determining if a call is homozygous for calls outside tandem repeats. However, there is a 31–35% difference for the number of calls detected in calls within tandem repeats when ranging the reciprocal overlap between 0.1 and 0.9 indicating that either the breakpoints are not well-defined, or that the SVs within tandem repeats are more diverse.

Large tandem repeat loci that are highly divergent from the reference are likely to have many different alignments with similar alignment scores but different SV representation, often with multiple SV calls within the same tandem repeat locus. To investigate the clustering of SVs within tandem repeat loci, all tandem and STR annotations from hg38 were merged (with an additional 20 base pairs on either side to account for under-annotation of tandem repeats) ~1 M tandem repeat loci. The distribution of number of calls per tandem repeat locus is shown for all callsets from Phased-SV and MS-PAC in Supplementary Figure 9 and summarized in Supplementary Data 26. The majority of tandem repeat loci have less than three SVs at each locus. For all tandem repeat loci with six or more SVs detected within the locus in either haplotype, we removed SVs from both haplotypes and assembly methods in each cluster, and store the alternative haplotype sequences. This covered on average of 285 loci and 1812 SVs from each haplotype (Supplementary Data 26). After removing SV calls from large clusters of SVs, SVs were merged between haplotypes using a 50% reciprocal overlap.

To decide between which representation of a Phased-SV or MS-PAC call to use when both overlapped and were validated by a BNG call, the optimal overlap with BNG was used (Supplementary Data 22). Each variant from Phased-SV and MS-PAC that overlapped a BNG call was assigned a fractional value *f*_BN_ = |L_BN_–L_PB_|/L_BN_ where L_PB_ is the length of the variant from the Phased-SV or MS-PAC, and L_BN_ is the length of the BNG variant. Variants with *f* *<* 0.1 were considered validated, regardless of whether or not the PBRC was >3. For NA19240, this selected 35 SVs (average 9496 bp) from Phased-SV, and 534 (average 4168 bp) from MS-PAC. The procedure recovered ~218 SVs per sample that would have been filtered by low PBRC. These were typically large SVs (mean size, 5.35 kb). Next, because the validation rate of the Phased-SV calls was greater, we included all remaining calls from Phased-SV that had PBRC >3. For NA19240 this included 10,064 deletions (mean 507 bp) and 15,244 insertions (mean 507 bp). We then added all calls from MS-PAC that were at least 10 kb away from a call in Phased-SV; for NA19240 this added 514 deletions (mean 341 bp) and 872 insertions (mean 366 bp). The categories of SV types are given in Supplementary Data 27.

### Illumina-based SV detection

A total of 15 algorithms, i.e., Delly, dCGH, ForestSV, Genome STRiP, HOLMES, Lumpy, Manta, MELT, novoBreak, Pindel, retroCNV, SVelter, Tardis, VariantHunter, and WhamG (Supplementary Methods 3.3; Supplementary Data 28) were applied to the parent–child trios to discover SVs from IL short-read sequences. Number of SVs reported by individual algorithm varies from 1000 to 23,000 (Supplementary Data 32), indicating large variance in sensitivity and FDR across methods.

To derive a consensus SV set, an experimental-based approach was developed where the breakpoint accuracy of individual algorithm was first estimated through comparisons against high-quality PB-SVs that have single base breakpoint resolution, then clustered by overlapping their confidence interval to derive consensus breakpoints with minimized confident intervals (Supplementary Figure 10).

### Breakpoint accuracy estimation

The breakpoint accuracy of each short-read-based algorithm was estimated by comparing against high confident PB-SVs by 50% reciprocal overlap (RO), i.e., for each SV predicted by short-read technology, if there is a PB-SV that partially/fully covers the same genomic region, where the overlap between the two calls exceeds half of both calls, a ‘match’ will be assigned and the distance between the PB breakpoints and the short-read ones will be recorded. The 10% and 90% quantile of each distribution will be calculated and assigned as the confidence interval (CI) of breakpoint accuracy for each algorithm. The distance between IL and PB breakpoints for 15 methods are shown in Supplementary Figure 11.

### Breakpoint clustering

Breakpoints of SVs from different algorithms were firstly clustered into candidate groups if their CIs overlap with each other. In each group, if a minimized common CI can be defined by taking the right most of the left breakpoints and the left most of the right breakpoint, the consensus breakpoint will be assigned as the one that is most frequently proposed by different algorithm. However, there are other situations where not all CIs overlap with each other. In this situation, a two-step approach will be adopted, where

1. All the CIs will firstly be stacked up and the number of CIs each breakpoint goes through is counted, with each peak and their right neighbor assigned as a CI.
2. Then the breakpoints within this cluster will be assigned to their nearest CIs to form the sub breakpoint clusters. And consensus breakpoints will be assigned in the same way as described above.

### Quality control for Illumina callset

An integrated set of 44,505 unique SVs was collected from the pipeline described above, out of which 421 fell within either telomere or centromere of a chromosome and were labeled to be removed from further genotyping. There are also 594 SVs of over 1 Mb in size that were labeled and removed in the primary quality control (QC) step. In the rest of the cohort, most of the integrated SVs were supported by only one algorithm (singleton, *n* = 22,903, perc = 65%), over half (*n* = 11,778) of which were found to overlap with another merged SV with support from multiple algorithms (clusters). Singletons that overlap with clusters are assumed to be well represented by the clusters, so that they are labeled as ‘redundancy’. The remaining SVs are labeled as ‘PASS’ and sent for downstream analysis, such as genotyping across trios, breakpoint accuracy assessment through GRAPHITE (https://github.com/dillonl/graphite) and VaPoR^60^.

### Unified Illumina SV callset

The integration pipeline described above resulted in a non-redundant set of IL-specific SVs consisting of 27948 unique SVs, including 17799 deletions, 2211 duplications, 3310 insertions, 3698 mobile element insertions and 930 other SVs, with 56% detected by more than one approach, and 49% with orthogonal support from other technologies (Supplementary Data 30).

We next assessed our integrated set by comparison with the high-quality PB-SV set and observed 49% events with a high degree (50%) of reciprocal overlap. We further identified ~1000 SVs per proband which appeared to be IL-specific. An in-depth investigation of these events showed signatures of structural rearrangements in the PB sequence data, suggesting their omission was due to methodological rather than technological limitations (Supplementary Figure 12, Supplementary Data 31). We also investigated the sequence context around the breakpoints of each set and observed stark differences within repetitive regions between the technologies (Supplementary Figure 13).

IL genotyping: In order to assess the allele frequency of the SVs discovered in the three trios we used the Simons Genome Diversity Project (SGPD) samples. A panel of 260 PCR-free genomes at moderate coverage (Supplementary Figure 14) represents 127 distinct populations from seven superpopulations^61,62^. The geographic distribution of the individual samples can be seen in Supplementary Figure 15. The coverage of these genomes increases our power to genotype SVs discovered by PB- and IL-based SV callers.

For base pair-resolved insertions and deletions, where both alleles are known, we applied SMRT-genotyper^13^. For the IL-only SV calls we applied SVTyper and Genome STRiP. The original SGDP pair-end data were lifted from hg19 to GRch38 using bwa-kit “run-bwamem.”

### Genomic coverage of Illumina and PacBio

Physical coverage of IL reads with mapping quality ≥15 (generous) and raw coverage of PB reads with mapping quality ≥30 (conservative) were calculated in 100 bp bins from each child’s genome. To mitigate effects of deletions obscuring which regions are not covered by a technology, coverage was combined from each child for the two technologies. Filtering heterochromatic regions, chromosome Y, and any bin with five reads or fewer covering it, we found 749 kb (0.028% of the euchromatic genome) base pairs covered by PB and not IL, and 2.94 Mb (0.1% of the euchromatic genome) of sequence conversely covered by IL and not PB. These platform-unique sequences are enriched for segmental duplications; 82.0% of the PB-only regions were segmental duplication, and 83.9% of the IL-only regions were segmental duplication. Thus, we expect the majority of the calls to be accessible to both platforms.

### Filtering integrated Illumina callsets

We then searched for concordance between calls from the integrated IL, integrated PB, and BNG callsets. We began by developing filtered SV callsets. The PB calls were previously filtered by raw read support. To develop a filtered IL callset, each call was compared to a superset of variation from the PB integrated callset including: the set of unfiltered SV calls from the haplotype local assemblies in Phased-SV, SVs detected through individual PB reads, the coverage depth of the PB alignments, and the BNG-SV calls. For all comparisons except for the BNG and read-depth comparison, the following steps were performed to detect the concordance between a query SV and a dataset of SVs. First, all target SVs from the dataset within 1 kb of the breakpoints of the query SV are collected. Each SV from this set is compared against the query by taking the ratio of the shorter of the query and target SV to the length of the larger of the query and target SV, and the concordance is defined as the maximum of all of these ratios. A similar test was used for comparison to BNG calls; however, the SV position interval was used in place of breakpoint boundaries. Each IL call was considered concordant if at least one of these conditions held true:

1. The maximal concordance of a PB or BNG target SV is at least 0.5.
2. At least three reads have at least 0.7 concordance with the query SV.
3. The call is a deletion and the PB read depth is <25 over the region of the call.
4. There are at least three PB reads that show a shift in the dotplot that is concordant with the IL SV call. This mitigates alignment artifacts from (2).

The greater stringency for raw read overlap is to reduce false-positive concordance caused by spurious short indels. The number of integrated IL-SV calls that had a concordant SV was HG00514: 76.6% (5083/6637) (del) 81.1% (2798/3450) (ins), HG00733 77.0% 5025/6530 (del) 82.3% 2825/3431 (ins), and NA19240 78.6% (6068/7723) (del), and 82.6% (3416/4137). The number and fraction of integrated IL calls for each child is shown below.

We compared the validation rate by the number of methods supporting each call. For calls supported by a single IL algorithm, between 45.1 and 48.3% of deletion calls were supported by additional technologies, and 80.0–82.1% of insertion singleton calls were supported.

We looked for evidence of the integrated IL calls that were not found in the integrated PB callset within the original PB reads or the Phased-SV local assembly unfiltered calls.

### Filtering Bionano Genomics callset

To generate a filtered callset of the BNG deletion calls, each call was compared with the SVs detected from local assemblies in Phased-SV, and read depth, SVs detected in raw PB reads, and the PB read depth. Because a genomic interval and estimated variant length are given for each BNG variant, the read-depth is calculated by scanning the genomic interval and taking the average coverage within a sliding window of the length of the SV. The candidate coverage is taken as the minimum coverage. A call is considered validated if the minimal average coverage is less than 30 (compared to the ×40 average sequencing depth of each sample). An example of a validated homozygous variant, validated heterozygous variant, and region in which a deletion variant was predicted but no suitable coverage interval was found is shown below. The corresponding variant discovered in the PB integrated set is shown in red.

In total this finds concordance rates as follows: HG00514: 94.1% (871/926), HG00733: 94.1% (829/881), NA19240: 97.8% (984/1006). Because it was not possible to find concordance with insertion calls and read-depth, all insertion calls less than 15 Mb were considered concordant.

The number of BNG calls overlapping segmental duplications are:

HG00514: *N* = 268/1656, total bp = 9608546/26129625, 6.47X increase

HG00733: *N* = 276/1698, total bp = 9723721/26693605, 6.41X increase

NA19240: *N* = 283/1740 total bp = 11830199/30477301, 6.8X increase.

Increase is computed using the fraction of the genome annotated as segmental duplication of 0.056.

### Integration of Illumina, PacBio, and Bionano callsets

The filtered integrated IL and BNG calls were integrated with the integrated PB-SV callset. The IL and BNG callsets were queried against the integrated PB callset in a similar manner as the query against the validation sets, and the IL set was queried against the BNG callset for concordant matches. The variants for which a concordant match was found were recorded as shared between pairs of datasets. To construct an integrated dataset, we merged (1) calls unique to each filtered dataset, (2) calls shared between pairs of datasets, and (3) calls shared across all three datasets. For cases 2 and 3, the PB variant was used when available (e.g., shared IL/PB, and shared BNG/PB, or shared across all three), and the IL variant when shared between BNG and IL.

The gain in sensitivity for PB-SV only calls was largely in SVs less than 2 kb (Supplementary Data 32).

### Specificity of integrated Illumina dataset

The calls by individual algorithms were evaluated for concordance with the PB and BNG integrated datasets, which may be viewed as an estimate of the accuracy of each method. Because there are calls missed by the union of the integrated PB and BNG callsets, this is a lower bound on the estimate. The average concordance for deletion calls ranged between 41% and 98% with a median of 93%, and for insertion calls ranged between 4.6% and 97.3% (Supplementary Data 33). The insertion statistic is affected by comparing the coordinates of the duplication calls, which are defined by the interval of the source sequence, to the insertion calls in the integrated PB callset and the BNG calls, which are defined at the insertion sites. This has an effect of reducing the concordance of methods that produce duplication calls through discordant read depth measurement, such as dCGH and Genome STRiP.

We then sought to assess the concordance of callsets formed from the union or intersection of multiple methods. Because of the computational burden of running many algorithms, we considered combinations of up to four algorithms, and tested three separate conditions: accept all calls from the union of two algorithm callsets, all calls from the union of three callsets, and calls from at least two of three algorithms. The first two conditions represent scenarios where one is targeting maximal sensitivity, while the last targets specificity. The union of methods naturally increases the FDR while increasing the sensitivity, whereas the intersection of methods decreases FDR requiring a majority (e.g., two of three methods) rather than total consensus can increase sensitivity, both with the expected trade-off between increased sensitivity and FDR. The non-concordance rate (NCR): 1- concordance, is used as a proxy for FDR. Considering NA19240, by requiring two of three methods to agree on a call, to it is possible to form a deletion callset of 4191 calls with an NCR of 4.6% using the Manta, Pindel, and Lumpy methods. This is an increase of 5–55% over any of the methods individually while keeping the estimated FDR below 5%.

### Inversion detection using Strand-seq

In order to explore larger copy-neutral as well as more complex SVs that are typically excluded in SV studies (often due to confounding genome architectural features and methodological limitations), we incorporated strand-resolved sequencing data generated using Strand-seq. Strand-seq is a single-cell sequencing technology that involves sequencing reads from individual DNA strands following bromodeoxyuridine (BrdU) incorporation into dividing cells^13,63^. In contrast to conventional Massively Parallel Sequencing, Strand-seq maintains DNA strand directionality, which is used to directly separate sequence data derived from each chromosomal homolog. These data preserve the long-range structural information of individual homologs, which can be used to scaffold phasing data to build haplotypes (as described above) and allows copy-neutral SVs to be directly visualized^18,39^. In contrast to other sequencing techniques, this technology identifies inversions based on the directionality of reads contained within the inverted locus, rather than from reads spanning inversion breakpoints. This allows even those variants embedded within large, highly identical low copy repeats (that are inaccessible by regular short or long DNA read sequencing approaches^1^ to be located). Accordingly, Strand-seq has recently emerged as a new method to discover inversions across an extensive length scale from a few kilobases up to several Megabases in size^18,39^.

To map inversions in the family trios, we generated altogether 1064 Strand-seq libraries, yielding a cumulative sequencing depth ranging from 3.6 to 7.7× per genome, and covering up to 80% of all mappable bases (Supplementary Figure 16A). To remove potential inter-cell inconsistencies and increase coverage for variant detection, composite files were generated for each individual, as previously described^18,39^ (Supplementary Figure 16B, 16C). A composite file represents merged sequence data from multiple Strand-seq libraries with strand directionality preserved. Based on alignment to the reference genome, each read in the composite file was designated as being in the same orientation as the reference (‘reference’), or the opposite orientation to the reference assembly (‘non-reference’). This allows regions of inverted orientation to be identified and genotyped based on the proportion of non-reference reads at the locus, and a read ratio was calculated for as the number of reads in the non-reference orientation divided by total number of reads (Supplementary Figure 16D). Regions with higher read ratios contain a greater proportion of reads supporting an inverted allele, and thus this value can be used as proxy for the level of support for an inversion.

Using a sliding window approach (window size = 250 reads) local read ratios (i.e., proportion of non-reference reads) were calculated for each window in the composite file and putative SVs were located as chromosomal regions that exhibited a segmental change in strand orientation and contained >15% non-reference reads (Supplementary Figure 17A). This located up to 354 genomic loci per individual that contained a significant portion of reads in a non-reference orientation, suggestive of an inversion (Supplementary Data 34). We found a cluster of these regions had a read ratio >0.80 (which supports a homozygous inversion); however, there was a continuous distribution of regions with a ratio <0.75 (Supplementary Figure 17B), which suggested that a subset of loci identified in the composite files were non-diploid. Each locus was genotyped as being either homozygous reference, heterozygous inverted, or homozygous inverted using a Fisher exact test that allowed for a 2% level of background in the homozygous states and required a minimum of 50 reads to genotype (Supplementary Figure 17C). This resulted in 187–208 (74.2%) heterozygous and 63–77 (25.8%) homozygous loci per individual (Supplementary Data 35). The inversions showed a continuous size distribution that ranged between 448 bp and 3.7 Mb in length, with a mean of 208 kb (Supplementary Figure 17D). This *discovery set* of Strand-seq inversions together represent simple inversions, complex variants and reference assembly errors. For example, we identified 35 inversions that were homozygous in all nine individuals that likely represent reference assembly misorients, and were thus removed from the downstream analyses (Supplementary Data 36).

### Classifying Strand-seq inversions using orthogonal phase data

The Strand-seq discovery set of inversions identified from the composite files were further classified by integrating orthogonal phasing data. We used a trio-aware PB-phased vcf file that was assembled using the WhatsHap^15,16^ independent of Strand-seq haplotype data. We tagged all possible reads in the single-cell libraries by identifying heterozygous SNVs captured in a Strand-seq sequencing read and assigned the fragment to either haplotype 1 (H1) or haplotype 2 (H2) based on agreement with the PB phasing file (Supplementary Data 37). Haplotagged composite files were then regenerated using the phased Strand-seq data and the haplotype structure at each locus was assessed in a strand-aware fashion—meaning we considered the ratio of phased H1:H2 reads in the reference orientation independent of the ratio of phased H1:H2 reads in the ‘non-reference’ orientation. This allowed us to assign a haplotype ratio for each strand of the locus, calculated as the proportion of reads with an H1 phase divided by the total number of phased reads. By combining read ratios with haplotype ratios, we defined distinct signatures to distinguish simple homozygous, heterozygous and complex heterozygous (e.g., inverted duplications) inversions identified in the Strand-seq discovery set.

We identified simple homozygous inversions (i.e., having a non-reference orientation for both homologs, and without an accompanying copy number change) as loci that were genotyped as homozygous and showed a haplotype ratio ~0.5 (>0.25 and <0.75) on the non-reference strand, where a minimum of ten phased reads were required (Supplementary Figure 17). Across all individuals we found 147 loci with this signature, of which 55 were unique. These simple homozygous inversions ranged in size from 2.2 kb to 3.9 Mb (median length = 142 kb). The median read ratio was 0.95 (range: 0.79–1.0), and the median haplotype ratio for the non-reference reads was 0.49, with an interquartile range of 0.10.

We then identified simple heterozygous inversions (i.e., loci showing non-reference orientation for a single homolog and without a copy number change) as loci genotyped as heterozygous and represented by different haplotypes on the reference and non-reference strands (Supplementary Figure 17). These loci had a haplotype ratio >0.75 on the reference strand coupled with a haplotype ratio <0.25 on the non-reference strand, or vice versa, where a minimum of ten-phased reads were required on each. This located 221 simple heterozygous inversions in all individuals, representing 102 unique loci. The lengths ranged between 2.9 kb and 3.9 Mb, and a median of 136 kb. The median read ratio for these events was 0.48 (range: 0.21–0.73) and the median haplotype ratio was 0.23 for the non-reference reads and 0.79 for the reference reads. The haplotype information was subsequently used to directly phase these inversions, where a non-reference haplotype ratio >0.75 marked an inversion on H1 (“1/0”), and a ratio <0.25 marked an inversion on H2 (“0/1”). This located 106 heterozygous inversions to H1, and 115 to H2.

Next, complex heterozygous inversions were identified as loci showing non-reference orientation and represented by phase data suggestive of a copy number change. These were located as events with a heterozygous genotype where a single haplotype was found on the non-reference strand (i.e., haplotype ratio <0.25 or >0.75) and both haplotypes were found on the reference strand (showing a haplotype ratio of ~0.5). This located 207 complex inversions, of which 97 were unique. The read ratio (median = 0.31; range = 0.18–0.79) of these inversions was slightly lower than the simple heterozygous, suggestive of a copy number increase on the reference strand (Supplementary Figure 18). They ranged in length from 2.1 kb to 1.3 Mb, with a median size of 72.5 kb. Taken together, this analysis located 129 simple and 98 complex inversions in the Strand-seq that have orthogonal support from PB phase data.

### Integrating inversion calls across orthogonal platforms

Although Strand-seq is uniquely positioned to locate large (kilobase-scale) inversions, even if embedded within highly repetitive segmental duplications, due to the sparsity of single-cell sequence data, the ability to locate smaller variants is limited using this method alone. For instance, the interquartile range of the Strand-seq inversion discovery set was 17.5–189.2 kb in length. To locate inversions of a complete size spectrum, we additionally generated inversion callsets from sequence data derived from PB, IL, BNG, and liWGS (corresponding to jumping libraries of 3.5 kb and 7 kb insert lengths) technologies (Supplementary Figure 19).

To unify all inversion predictions into a single integrated callset, we first performed an intersection test that measured the level of reciprocal overlap between the different technologies. For all intersecting inversions, we calculated the level of overlap by dividing the number of intersecting base pairs by the total predicted inversion length. We then filtered pairs that showed >50% reciprocal overlap to identify inversions supported by two independent technologies (Supplementary Figure 20).

The results of this test yielded a total of 1296 supported inversions (137 non-redundant), with 37–96 per individual (Supplementary Data 38). Approximately half (45.9%) were recovered for each parent because PB only produced variant calls for the probands. Of the total supported inversions, 533 came from IL, 335 from liWGS libraries, 172 from PB, 147 from Strand-seq and 106 from BNG callsets (Supplementary Figure 21A). While BNG contributed the fewest number of inversions to the total, 39.1% of the initial callset was supported, whereas only 13.6% of the initial IL callset (that contributed the most) was supported (Supplementary Figure 21B). Notably, although a cutoff of 50% reciprocal overlap was used, the majority of passing events showed near complete agreement (first quartile of percent overlap was 89.5%). The majority (64.9%) of which were represented by the minimum of two technologies, with only 5.5% represented by all (Supplementary Figure 21C). We found IL and PB showed the most agreement at a size scale <2 kb, the jumping libraries overlapped with IL within the size range 2–4 kb, and with PB and/or Strand-seq between 4 and 25 kb. The highest agreement between technologies was seen between 5 and 50 kb size lengths, with the larger events (>50 kb) dominated by Strand-seq and BNG calls (Supplementary Figure 20; Supplementary Figure 21D). Indeed, because the interquartile range of intersecting inversions was 1.9–18.7 kb (median 3.5 kb), the majority of inversions predicted by Strand-seq were poorly represented by this test (Supplementary Figure 21B). This points to the distinct strategy used by the method to locate inversions that cannot be captured by orthogonal technologies.

To additionally recover inversions not captured in the intersection test, we next performed a genotyping test using the Strand-seq composite files. We performed a Fisher exact test on all inversion callsets to test whether support was seen in the Strand-seq composite files, where a minimum of 25 reads were required to genotype the locus. From the discovery callsets we found a striking number of predicted inversions with a ‘reference’ genotype in the Strand-seq files, where between 36.6 and 76.1% of genotyped loci (i.e., containing >25 reads in the composite file) were unsupported (Supplementary Data 39). Nevertheless, this test was able to add orthogonal support to a total of 175 non-redundant predicted inversions, 84 of which were not present in the interception test results. Moreover, when we repeated this genotyping test using only inversions passing the interception test, we found the number of inversions supported by the composite files increased up to 36.4% (Supplementary Data 40). For example, 89 (36.6%) of the inversions predicted in the PB callset were not supported by Strand-seq in the initial test, and this dropped to a single (0.6%) unsupported event when the filtered set was genotyped. This analysis illustrates that both the Strand-seq re-genotyping and interception analyses are useful validation tools for inversion discovery.

From these analyses a final unified inversion callset was generated that represented variants supported by at least two orthogonal technologies. To obtain this, we compiled all supported inversions from the (i) interception test, (ii) Strand-seq genotyping test, and (iii) Strand-seq inversions supported by PB phase data (Supplementary Figure 22). The three supported inversion lists were merged into a non-redundant inversion set for each individual. Any pericentric events (e.g., large >6 Mb inversion predicted by IL and BNG), or those with a tandem repeat fraction >90% were removed, and breakpoint ranges were determined for the remaining inversions. For this, the outer breakpoint of the inverted region (InvR) was defined as the outermost start and end positions of each non-redundant event found between all three lists. For all events containing >1 inversion prediction within the locus (e.g., those located in the interception test), the inner breakpoints (innerBP_start and innerBP_end) were defined as the consensus region represented by at least half of the overlapping predictions. If the inversion was listed in only one of the support lists (e.g., those discovered by Strand-seq and supported with phase data) the innerBPs were set to match the InvR. A consensus genotype was then assigned to the inversion by taking the majority genotype call found for all discovery sets. If no majority call was possible (e.g., an equal number of heterozygous and homozygous genotypes were listed for all technologies) the genotype was listed as ‘ambiguous’. Finally, we classified the unified list into simple (Supplementary Data 41 and Supplementary Data 42) and complex (Supplementary Data 43 and Supplementary Data 44) inversions, by performing a copy number analysis. Here, we called the copy number state using Genome STRiP on the high-depth IL data to assign a copy number value for each inversion.

### Project analyses members

Ankit Malhotra, David Porubsky, Tobias Rausch, Eugene J. Gardner, Oscar L. Rodriguez, Li Guo, Ryan L. Collins, Xian Fan, Jia Wen, Robert E. Handsaker, Susan Fairley, Zev N. Kronenberg, Xiangmeng Kong, Fereydoun Hormozdiari, Dillon Lee, Aaron M. Wenger, Alex R. Hastie, Danny Antaki.

### Principal investigators

Steven McCarroll, Goo Jun, Li Ding, Chong Lek Koh, Bing Ren, Paul Flicek, Ken Chen, Mark B. Gerstein, Pui-Yan Kwok, Peter M. Lansdorp, Gabor T. Marth, Jonathan Sebat, Xinghua Shi, Ali Bashir, Kai Ye, Scott E. Devine, Michael Talkowski, Ryan E. Mills, Tobias Marschall, Jan O. Korbel, Evan E. Eichler, Charles Lee.

### Reporting summary

Further information on experimental design is available in the Nature Research Reporting Summary linked to this article.

## Supplementary information

Supplementary Information

Description of Additional Supplementary Files

Supplementary Data 1-71

Reporting Summary

## Data Availability

Underlying sequencing read data from the various platforms can be accessed via the International Genome Sample Resource (IGSR)^64^ at http://www.internationalgenome.org/data-portal/data-collection/structural-variation. Indel variant calls will be made available with dbSNP build B151. SV calls are made available under dbVar accession nstd152. All other relevant data are available upon request.

## Code availability

Phasedsv: https://github.com/mchaisso/phasedsv;

MsPac: https://bitbucket.org/oscarlr/mspac;

HGSV_SV_integration_pipe: https://github.com/xuefzhao/HGSV_SV_integration_pipe;

InversionAnalysis_HGSVC: https://github.com/drashley/InversionAnalysis_HGSVC.
