# Supplementary material for: Multi-platform discovery of haplotype-resolved structural variation in human genomes
Source: Nat Commun. 2019 Apr 16;10:1784. doi: 10.1038/s41467-018-08148-z (PMC6467913; doi:10.1038/s41467-018-08148-z)
Supplement: Supplementary file 1 — Supplementary Information [file 41467_2018_8148_MOESM1_ESM.pdf]

SUPPLEMENTARY INFORMATION

# Multi-platform discovery of haplotype-resolved structural variation in human genomes

Mark J.P. Chaisson[1,2]*, Ashley D. Sanders[3]*, Xuefang Zhao[4,5]*, Ankit Malhotra[6], David Porubsky[7,8], Tobias Rausch[3], Eugene J. Gardner[9], Oscar L. Rodriguez[10], Li Guo[11,12,13], Ryan L. Collins[5,14], Xian Fan[15], Jia Wen[16], Robert E. Handsaker[17,18,19], Susan Fairley[20], Zev N. Kronenberg[1], Xiangmeng Kong[21,22], Fereydoun Hormozdiari[23,24], Dillon Lee[25], Aaron M. Wenger[26], Alex R. Hastie[27], Danny Antaki[28], Thomas Anantharaman[27], Peter A. Audano[1], Harrison Brand[5], Stuart Cantsilieris[1], Han Cao[27], Eliza Cerveira[6], Chong Chen[15], Xintong Chen[9], Chen-Shan Chin[26], Zechen Chong[15], Nelson T. Chuang[9], Christine C. Lambert[26], Deanna M. Church[29], Laura Clarke[20], Andrew Farrell[25], Joey Flores[30], Timur Galeev[21,22], David U. Gorkin[31,32], Madhusudan Gujral[28], Victor Guryev[7], William Haynes Heaton[29], Jonas Korlach[26], Sushant Kumar[21,22], Jee Young Kwon[6,33], Ernest T. Lam[27], Jong Eun Lee[34], Joyce Lee[27], Wan-Ping Lee[6], Sau Peng Lee[35], Shantao Li[21,22], Patrick Marks[29], Karine Viaud-Martinez[30], Sascha Meiers[3], Katherine M. Munson[1], Fabio C. P. Navarro[21,22], Bradley J. Nelson[1], Conor Nodzak[16], Amina Noor[28], Sofia Kyriazopoulou-Panagiotopoulou[29], Andy W. C. Pang[27], Yunjiang Qiu[32,36], Gabriel Rosanio[28], Mallory Ryan[6], Adrian Stütz[3], Diana C.J. Spierings[7], Alistair Ward[25], AnneMarie E. Welch[1], Ming Xiao[37], Wei Xu[29], Chengsheng Zhang[6], Qihui Zhu[6], Xiangqun Zheng-Bradley[20], Ernesto Lowy[20], Sergei Yakneen[3], Steven McCarroll[17,18,38], Goo Jun[39], Li Ding[40], Chong Lek Koh[41], Bing Ren[31,32], Paul Flicek[20]§, Ken Chen[15]§, Mark B. Gerstein[21,22,42,43]§, Pui-Yan Kwok[44]§, Peter M. Lansdorp[7,45,46]§, Gabor T. Marth[25]§, Jonathan Sebat[28,31,47]§, Xinghua Shi[16]§, Ali Bashir[10]§, Kai Ye[12,13,48]§, Scott E. Devine[9]§, Michael E. Talkowski[5,19,49]§, Ryan E. Mills[4,50]§, Tobias Marschall[8]§, Jan O. Korbel[3,20]‡§, Evan E. Eichler[1,51]‡§, Charles Lee[6,33]‡§

* These authors contributed equally to this work.

§ These authors are co-senior.

‡ Correspondence to jan.korbel@embl.de, eee@gs.washington.edu, or charles.lee@jax.org

## Table of Contents


Supplementary Methods ... 5
1. Sample collection and data generation ... 5
1.1 Data generation and preprocessing ... 5
1.2 Data availability ... 12
2. Chromosomal haplotype resolution and integration ... 12
2.1 Mapping of meiotic recombination events ... 12
2.2 Strand-seq phasing ... 13
2.3 Mapping of meiotic recombination events using Strand-seq ... 13
3. Full-spectrum variation detection ... 14
3.1 Short insertion and deletion (<50bp) ... 14
3.2 Haplotype-resolved SV characterization and integration ... 15
3.3 Variation detected by short-read sequencing ... 19
3.4 Down - sample analysis of the HGSV Illumina callset ... 24
4. Integration of Illumina, PacBio, and Bionano callsets ... 25
4.1 Comparison of read depth and de novo assembly methods ... 25
4.2 Genes affecting SVs ... 25
5. Resolving Inversion Sequence Errors in the Human Reference Genome ... 26
6. Biological context ... 27
6.1 Population genetics of SVs ... 27
6.2 Functional analysis and annotations ... 27
6.3 MEI discovery and analysis with PacBio data ... 31
Description of Supplementary Figures ... 33
Supplementary Figure 1 ... 35
Supplementary Figure 2 ... 36
Supplementary Figure 3 ... 37
Supplementary Figure 4 ... 38
Supplementary Figure 5 ... 39
Supplementary Figure 6 ... 40
Supplementary Figure 7 ... 41
Supplementary Figure 8 ... 42
Supplementary Figure 9 ... 43
Supplementary Figure 10 ... 44
Supplementary Figure 11 ... 45
Supplementary Figure 12 ... 45
Supplementary Figure 13 ... 46

Supplementary Figure 14 ..... 47
Supplementary Figure 15 ..... 48
Supplementary Figure 16 ..... 49
Supplementary Figure 17 ..... 50
Supplementary Figure 18 ..... 51
Supplementary Figure 19 ..... 52
Supplementary Figure 20 ..... 53
Supplementary Figure 21 ..... 54
Supplementary Figure 22 ..... 55
Supplementary Figure 23 ..... 56
Supplementary Figure 24 ..... 57
Supplementary Figure 25 ..... 58
Supplementary Figure 26 ..... 59
Supplementary Figure 27 ..... 60
Supplementary Figure 28 ..... 61
Supplementary Figure 29 ..... 62
Supplementary Figure 30 ..... 63
Supplementary Figure 31 ..... 65
Supplementary Figure 32 ..... 66
Supplementary Figure 33 ..... 67
Supplementary Figure 34 ..... 68
Supplementary Figure 35 ..... 69
Supplementary Figure 36 ..... 70
Supplementary Figure 37 ..... 71
Supplementary Figure 38 ..... 72
Supplementary Figure 39 ..... 73
Supplementary Figure 40 ..... 74
Supplementary Figure 41 ..... 75
Supplementary Figure 42 ..... 76
Supplementary Figure 43 ..... 78
Supplementary Figure 44 ..... 80
Supplementary Figure 45 ..... 81
Supplementary Figure 46 ..... 82
Supplementary Figure 47 ..... 83
Supplementary Figure 48 ..... 84
Supplementary Figure 49 ..... 85
Supplementary Figure 50 ..... 86
Supplementary Figure 51 ..... 87

Supplementary Figure 52 .......... 88
Supplementary Figure 53 .......... 89
Supplementary References .......... 90

# Supplementary Methods

## 1. Sample collection and data generation

### 1.1 Data generation and preprocessing

#### Cell lines

Transformed lymphoblast cell lines from three parent-child trios (**Supplementary Figure 23**) belonging to the 1000 Genomes Project were obtained from the Coriell Cell Repository as part of the NHGRI catalog (https://catalog.coriell.org/1/NHGRI ).

#### PCR-free deep Illumina-sequencing

**Library preparation and sequencing (Contributors: Sau Peng Lee, Ching Lek Koh, Korlach, Munson, Eichler, Lee, JE and Lee, C):** DNA was extracted and its' OD260/280 ratio confirmed to be between 1.8 – 2.0. The quality of the DNA was further evaluated by using a PicoGreen® dsDNA Assay (Invitrogen). DNA libraries were prepared according to the Illumina TruSeq DNA PCR-Free Library prep protocol. For each DNA library preparation, 2 ug of high molecular weight genomic DNA was randomly sheared using the Covaris S2 system to 550 bp fragments. The fragments were blunt ended, phosphorylated, and a single 'A' nucleotide was added to the 3' ends of the fragments in preparation for ligation to an adapter that has a single-base 'T' overhang. Adapter ligation at both ends of the genomic DNA fragment conferred different sequences at the 5' and 3' ends of each strand in the genomic fragment. The quality of the DNA libraries was verified by capillary electrophoresis (Bioanalyzer, Agilent), clustered on the Illumina cBOT station and paired-end sequenced for 125 cycles on the HiSeq 2500 sequencer according to the Illumina cluster and sequencing protocols.

**Sequence data processing (Contributors: Fairley, Clarke, Zheng, Lowy and Flicek):** In addition to data coordination and distribution, the International Genome Sample Resource (IGSR)[1] provided alignment of Illumina whole-genome sequence data (PCR-free high coverage[SF1] ) from the nine individual genomes. Data was aligned to the GRCh38 assembly in an alt-aware manner using bwa-mem[2]. Details of the pipeline are in this file: http://ftp.1000genomes.ebi.ac.uk/vol1/ftp/data_collections/hgsv_sv_discovery/README.Illumina_wgs.GRCh38.alignment. The raw data and alignments are listed in the files Illumina_wgs.sequence.index and Illumina_wgs.GRCh38.alignment.index in the same directory.

Further statistics from the alignments can be found in the .bas files that sit alongside the alignment files and **Supplementary Data 45**.

#### 3.5 Kb Long-insert whole genome sequencing

**Library preparation and sequencing (Contributors: Talkowski, Collins, Brand, Stone, Glessner):** We generated long-insert whole-genome sequencing (liWGS) libraries for all nine individuals from the three HGSVC trios with a protocol that has been previously described[3]. In brief, 5.0 µg of genomic DNA from lymphoblastoid cell lines for each individual was sheared with a Covaris E220 sonicator and size selected to a target fragment size of 3,500bp (targeted range: 2,500-5,000 bp). These ~3.5 Kb fragments were circularized around a biotinylated adapter oligo and digested with EcoP15I restriction enzyme, followed by streptavidin bead-based capture of the biotinylated circularization junction and preparation of fragments for Illumina TruSeq sequencing with paired-end 25bp reads per Illumina's standard protocols. Sequencing was performed on an Illumina HiSeq2500 at The Broad Institute to a mean depth of 191.9 million read-pairs per library.

**Sequence data processing (Contributors: Talkowski, Collins, Brand, Stone, Glessner):** Quality of raw sequencing reads was evaluated using FastQC[4] prior to alignment, then libraries were aligned against the

GRCh37 and hg38 primary assemblies with BWA-backtrack v0.7.10-r789[5]. Duplicates were marked with SAMBLASTER v0.1.1[6] and all subsequent alignment processing, including sorting and indexing, was performed with sambamba v0.4.6[7]. Alignment quality was assessed with the Picard suite v1.115 (https://broadinstitute.github.io/picard/), Samtools v1.0 [8], and BamTools v2.2.2[9]. Library production generated an average insert of 3,475bp, and a mean physical coverage of 158.8X per library. Insert size distributions and alignment statistics are provided in **Supplementary Figure 24** and **Supplementary Data 46**.

## 7.5 Kb Mate-pair sequencing

**Library preparation and sequencing (Contributors: Stuetz):** Long-range (or 'Mate-pair') DNA library preparation was carried out using the Nextera Mate Pair Sample Preparation Kit (Illumina). In brief, 5μg of high molecular weight genomic DNA were fragmented by the Tagmentation reaction in 400ul, followed by the strand displacement and AMPure XP (Agencourt) cleanup reaction. Samples were size selected to 6.5-8.5 Kb with a gel step following the Gel-Plus path of the protocol. 350-500ng of size-selected DNA were circularized in 400ul for 16h at 30° C. The library was then constructed after an exonuclease digestion step to get rid of remaining linear DNA, fragmentation to 300-700bp with a Covaris S2 instrument (LGC Genomics), binding to streptavidin beads and Illumina TruSeq adapter ligation. Final library was obtained after PCR for 1min @ 98°C, followed by 11 cycles of 30sec @ 98°C, 30sec @ 60°C, 1min @ 72°C and a final 5min @ 72°C step and another gel size selection step. Deep sequencing was carried out with the Illumina HiSeq2000 (2x101bp) instrument using v3 chemistry to reach an average physical coverage of 30x.

**Data preprocessing of the 7.5 Kb library (Contributors: Meiers, Rausch):** The Illumina Nextera Mate Pair protocol was used to generate a 7.5 Kb insert library for all 9 samples. Nextera Mate Pair data cannot be trimmed with standard adapter trimming tools because of the circularization-based library preparation but specialized tools such as nxTrim and NextClip exist. In this project we used NextClip with default parameters. All remaining read pairs were aligned using the EBI's GRCh38 alignment pipeline detailed in this README (ftp://ftp.1000genomes.ebi.ac.uk/vol1/ftp/data_collections/hgsv_sv_discovery/README.Illumina_wgs.GRCh38.alignment). Briefly, reads were aligned using bwa mem, de-duplicated using BioBamBam and converted to CRAM format using Cramtools. The alignment index is available at ftp://ftp.1000genomes.ebi.ac.uk/vol1/ftp/data_collections/hgsv_sv_discovery/.

## PacBio SMRT Sequencing

**Library preparation and sequencing (Contributors: Korlach, Munson):** For the Puerto Rican trio (HG00731, HG00732, HG00733), high molecular weight DNA was prepared from cultured cells using the Gentra PureGene kit and a modified protocol (http://www.pacb.com/documentation/unsupported-protocol-gentra-puregene-qiagen-dna-isolation/). DNA integrity was confirmed by visual inspection of 1% agarose gel and the appearance of a single HMW band. SMRTbell libraries were constructed using the SMRTbell Template Prep Kit 1.0, according to the protocol described in: "Procedure & Checklist – 20 Kb Template Preparation Using BluePippin Size-Selection System" (Pacific Biosciences, Menlo Park CA). The genomic DNA was mechanically sheared using the Megaruptor system (Diagenode, Denville NJ) to yield an average shear size distribution of 30-35 Kb for the Han Chinese & Puerto Rican trio samples, and gTubes (Covaris, Woburn MA) for the Yoruban trio samples (20-25 Kb average shear size distribution). The libraries were then subjected to a size-selection step using the BluePippin system (SageScience, Beverly MA) to remove shorter DNA inserts, with size cutoffs of 17 Kb (Han Chinese & Puerto Rican) and 10 Kb (Yoruban), respectively. Library quality and quantity were assessed using the Pippin Pulse field inversion gel electrophoresis system (SageScience), as well as the Qubit dsDNA Broad Range Assay kit and Qubit Fluorometer (Thermo Fisher). An additional library was constructed for HG00733 at the University of

Washington: Isolated DNA was diluted to 50 ng/uL and sheared using Megaruptor (Diagenode) at 35 Kb (PacBio) or 40 Kb (UW) settings and SMRTbell libraries were generated as described above with a BluePippin size selection of 15 Kb.

All SMRT sequencing was performed on the Pacific Biosciences RS II using on-plate concentrations of 100-150 pM (PacBio libraries) or 250 pM (UW library). Data were generated using the P6-C4 sequencing chemistry, with magnetic bead loading and 240 or 360 minutes' movie times. Sequencing was performed at 4 centers: Jackson Laboratory (YRI trio), University of Washington and Ontario Institute for Cancer Research (PUR trio) and University of Malaya (CHS trio). Children were targeted with a sequencing depth of ~40-fold coverage and parents for ~20-fold sequence coverage with the goal of providing 30-fold sequence coverage per haplotype (**Supplementary Data 47**; **Supplementary Figure 25**). Average mapped sequence read lengths varied between samples: HAN: 9,583 bp PUR: 9475bp, and YRI: 5,528 bp.

### Oxford Nanopore Sequencing

**Library Preparation and Sequencing (Contributors: Dai, Harrington, Juul):** The integrity of the gDNA sample of the Puerto Rican daughter (HG00733) was checked on a 0.8% agarose gel. A high molecular weight band was observed in addition to a visible smear ranging from as low as 1 Kb (data not shown). In order to optimize both yield and read length, 6 libraries were prepared using different combinations of shearing, size-selection and Oxford Nanopore Technologies sequencing kit. DNA was sheared by g-tube (Covaris, Woburn MA) at 3500 rpm for 1 minute. Size selection was carried out on a BluePippin system using the BLF7510 0.75% Agarose Gel cassette (SageScience, Beverly MA) using a cutoff of 10 Kb. All sequencing was performed on Oxford Nanopore Technologies' GridION X5 using FLO-MIN106 (R9.4) flowcells for 48 hours. Basecalling was carried out in realtime on the GridION X5 using the guppy basecaller (v.0.3.0). Reads were post-processed to remove those shorter than 2Kb and with a mean quality score of less than 7.

### Bionano Optical mapping

**Bionano DNA labeling (Contributors: Hastie , Lee):** High-molecular-weight DNA from fresh cell lines was labelled following the IrysPrep Reagent Kit protocol. For 2 hours at 37 °C, DNA was digested with nicking endonuclease Nb.BssSI (New England BioLabs). Nicked DNA was then incubated for 1 hour at 72 °C with fluorescently labelled dUTP and Taq Polymerase (New England BioLabs). Taq ligase (New England BioLabs) was used in the presence of dNTPs for ligation of nicks. DNA was counterstained with YOYO-1 (Thermo Fisher Scientific). All samples were also labeled with Nt.BspQI using optiDNA labeling kit. Cells are mixed with liquefied LMP agarose and deposited on a device for forming a thin layer of agarose/cell mixture. After solidification, the cells are lysed and proteins digested by protease K and lysis buffer. Following washes, DNA is nicked with Nt.BspQI at 37°C for 2 hours, labeled at 55°C for one hour using fluorescent nucleotide analogs and taq polymerase, DNA is then ligated with taq ligase, the thin layer is washed between steps. The agarose is dissolved and the DNA is stained before data collection.

**Bionano data collection:** DNA samples nicked with each nick endonucleases were loaded into IrysChips (Bionano Genomics) and run on the Irys (Bionano Genomics) system. Data were collected until approximately 100-fold coverage of long molecules (> 150 Kb) was achieved for both Nt.BspQI and Nb.BssSI samples.

**Bionano data preprocessing:** The IrysView (Bionano Genomics) software was used to detect linearized DNA using the YOYO-1 counterstain, and to detect the labelled nick sites on the DNA. The length of each molecule and the position of each label is output in bnx files. Molecules were filtered above 150 Kb. Sets of single-molecules, equivalent to about 100 x haploid coverage, for each sample, was then used to construct a *de novo* genome assembly.

## Strand-seq Methods

**Library preparation and sequencing (Contributors: Sanders, A.):** EBV-transformed lymphoblastic cell lines were cultured in BrdU (40uM final concentration) for 36 hours and single cells sorted into 96-well plates based on Hoechst-quenching[10]. Library construction was performed on a Bravo liquid handler (Agilent technologies), to automate MNase digestion of nuclear DNA, ligation of indexed Illumina adapters, and Hoechst/UV treatment to remove BrdU strands and prepare samples for 17 rounds of PCR amplification, as described[10].. Libraries from a single 96-well plate were pooled for post-PCR gel size selection, which enriched for the mononucleosomal fragment (~150bp + 120bp adapters) and dinucleosomal fragment (~320bp + 120bp adapters). For sequencing, 96 samples were from the mononucleosomal fragment was run on a single lane of an Illumina HiSeq (rapid-run mode), using a 76 bp paired-end protocol. 192 samples (two plates) of the dinucleosomal fragment were pooled and sequenced using a 151bp paired-end protocol.

**Sequence data processing (Contributors: Porubsky, D. Sanders, A.):** The Strand-seq raw sequencing data were demultiplexed based on the library-specific barcodes and converted to FASTQ files using Illumina standard software (bcl2fastq, version 1.8.4). Reads were aligned to GRCh38 human reference genome assembly, which includes decoy and HLA sequences. The FASTQ files were mapped to the reference genome using bwa aligner (version 0.7.12-r1039) according to the HGSVC guidelines for Illumina sequencing. Following alignment, reads were sorted using SAMtools[8] (version 1.2) and duplicate reads were marked using biobambam (version 0.0.191)[11]. Based on common library-specific barcodes, the separate BAM files for the mono- and di-nucleosome fraction of each cell were merged into a single BAM using SAMtools (version 1.2). Directional read distribution of each Strand-seq libraries was assessed using BAIT[12] to preselect Strand-seq libraries based on read density, level of background reads and level of variability[13]. BAM files passing our quality criteria served as an input for inversion calling and haplotyping pipeline.

## 10X Genomics

**Library preparation and Sequencing (Contributors: Jabara C.):** Cell lines were cultured and DNA extracted using the Qiagen MagAttract kit with modifications to enhance retention of long DNA molecules:

https://assets.contentful.com/an68im79xiti/ICEjig84zQWoWiKaws8QY/d0872d726cc797579e4a8273e640b35d/20160607_SamplePrepDemonstratedProtocol_-_DNAExtractionfromBlood_RevB.pdf

1.25 ng of high molecular weight DNA was loaded onto the 10x Chromium Controller using Chromium Genome v1 reagents following the recommended protocol:

https://assets.contentful.com/an68im79xiti/4z5JA3C67KOyCE2ucacCM6/d05ce5fa3dc4282f3da5ae7296f2645b/CG00022_GenomeReagentKitUserGuide_RevC.pdf

The initial part of the library construction takes place within droplets containing gel beads functionalized with barcodes that mark the droplet of origin (called GEMs). The library construction incorporates a barcode that is adjacent to read one. All molecules within a GEM get tagged with the same barcode, but because of the limiting dilution of the genome (roughly 300 haploid genome equivalents) the chance that two molecules from the same region of the genome are partitioned in the same GEM is very small. Thus, the barcodes can be used to statistically associate short reads with their source long molecule. The resulting library was sequenced on an Illumina X Ten sequencer to produce 2X150 paired-end sequences. The resulting data type is called 'Linked-Reads' (Zheng et al, 2016).

**Sequence data processing:** Sequence data was analyzed using the Long Ranger v.2.1 analysis pipeline. Briefly, reads are aligned using Lariat (https://github.com/10XGenomics/lariat), a wrapper around BWA that uses molecule information to adjust alignment locations and MAPQ, using the RFA methods [14] . This allows

for more reads to be confidently mapped. In these cases, map quality scores are adjusted so that downstream analysis can take advantage of these reads.

Single nucleotide variants (SNVs) and small indels are called using Freebayes (v0.9.21-7, default parameters (-0)).

Each read covering a heterozygous variant is re-aligned to a ~100bp segment of the reference sequence, with and without the alt allele applied, to determine whether the read gives clear support for one allele over the other. Mapping each read to a molecule via the GEM-specific barcode produces the yield of the set of alleles observed on each molecule. We model the likelihood of the per-molecule allele observations given a phasing configuration. The model follows [15] with additional terms to account for the small probability that a barcode carries two molecules from opposite haplotypes, and for the chance that an input variant is non-heterozygous. We search for the maximum likelihood phasing configuration by find near-optimal local configurations using beam-search over blocks of ~40 variants. Blocks are greedily joined to form a global solution, which we iteratively refine. The confidence of each phasing decision is the likelihood-ratio between optimal and next-best solutions. The phasing procedure solution implicitly produces a posterior distribution over haplotypes for each input molecule. Molecules covering >1 het are typically phased with very high confidence. We write this haplotype information to an auxiliary BAM tag on each read of a confidently phased molecule, which is used in downstream haplotype aware SV calling.

Input variants determined to be non-heterozygous are switched to HOM REF or HOM ALT.Variant calls are then emitted as a VCF file.

### TruSeq Synthetic Long Reads (TSLR)

**(Contributors : Sebat, J., Gabriel Rosanio, Danny Antaki, Masdhu Gujral, Joey Flores , Karine Viaud Martinez )**

The Illumina TruSeq SLR platform developed is based on the isolation of single molecules followed by amplification, barcoding and conventional Illumina sequencing (http://bit.ly/2kFgQDc). This method generates “Synthetic long reads” which consist of contigs that are assembled from the short read sequences derived from of a single molecule. The Illumina TruSeq SLR method is based on the same principle (http://bit.ly/2dmkEHK). A key advantage of this approach is the high sequencing accuracy afforded by the Illumina platform.

**gDNA purification**. Lymphocytes from individuals in a Yoruban (NA19238, NA19239, NA19240), a Han Chinese (HG00512, HG00513, HG00514), and a Puerto Rican (HG00731, HG00732, HG00733) trio were used to acquire genomic DNA. The DNA was purified from each individual separately using the DNeasy Blood & Tissue Kit as previously reported (Qiagen). Concentrations for each individual were determined using Qubit BR and confirmed by agarose gel electrophoresis.

**Sample prep.** Each individual’s gDNA was prepared for whole genome sequencing using the TruSeq synthetic long read prep kit according to manufacturer’s protocols with the exception the template concentration for each Long Range PCR reaction was 0.9fg/µl rather than the prescribed 0.6fg/µl. A total of 25 384-well plates were prepared for each sample.

**Sequencing**. Moleculo sequencing of prepared libraries were performed by Illumina, Inc. Paired-end sequencing was performed (add coverage) using an Illumina HiSeq 2000 (**Supplementary Data 48**).

**Data Processing.** Sequenced paired-end samples were separated by barcode identification into 384 separate bins using TruSeq Long-Read Assembly App (Illumina). Assembly of separated paired-end sequences with ≥Q30 into synthetic long read sequences was performed with the TruSeq Long-Read

Assembly App. Full documentation on this software can be found at http://support.Illumina.com/help/BS_App_LongReads_help/ TruSeq_Long_Reads_Assembly_App.htm

**Data Repository Information.** Resultant sequencing data (all separated paired-end sequences, synthetic long read assemblies, long read scaffold information, and a summary report) are available from the European Nucleotide Archive (https://www.ebi.ac.uk/ena/submit/sra/#home)

The pooled data from all preparations for these individuals showed common long read signatures: a drop off of assemblies at the arbitrary 1499bp/1500bp boundary; a decrease in longer assemblies; a peak around the expected ~10 Kb selection length. The average assembled long read for NA19238, NA19239, and NA19240 are 3939bp, 4075bp, and 3834bp respectively. The N50 for each individual is 6877bp, 7291bp, and 6723bp respectively.

**Alignment**. Synthetic long reads greater than 1499bp in length were aligned to GRCh38 using the LAST aligner. LAST was selected over others, including BWA and BLAST, because of its speed and ability to align reads to multiple locations in the genome to optimize the alignment without any a priori information.

Simulated long reads at a depth of 7.5X from GRCh38 were also aligned to GRCh38. This alignment was of high quality as expected. Only 7 breakpoints were detected indicating a low rate of misalignment by LAST.

SV calling was performed by split read analysis of the multiple alignment files generated by LAST. Deletion, Tandem Duplication, Insertion and Inversion signatures were parsed out of the alignments. The final raw call set included all SVs that were 50 bp or greater in length for which at least 2 reads supported the identical call.

**Sequence data processing**: Sequenced paired-end samples were separated by barcode identification into 384 separate bins using TruSeq Long-Read Assembly App (Illumina). Assembly of separated paired-end sequences with ≥Q30 into synthetic long read sequences was performed with the TruSeq Long-Read Assembly App. Full documentation on this software can be found at:

http://support.Illumina.com/help/BS_App_LongReads_help/ TruSeq_Long_Reads_Assembly_App.htm

Resultant sequencing data (all separated paired-end sequences, synthetic long read assemblies, long read scaffold information, and a summary report) are available from the European Nucleotide Archive (https://www.ebi.ac.uk/ena/submit/sra/#home)

### Hi-C data generation

**Library preparation and Sequencing**: Hi-C was performed according to established methods[16,17], and using the restriction enzyme HindIII (NEB R3104) for digestion of chromatin prior to proximity ligation. Lymphoblastoid cell lines were obtained from Coriell Cell Repositories. Two independent biological replicates were performed for each cell line, using approximately 20 million cells per replicate. Cells for each replicate were cultured independently for at least 2 passages. Hi-C libraries were sequenced on an Illumina HiSeq (2000, 2500, or 4000), generating roughly 250 million reads per replicate.

**Hi-C data processing and phasing** (**Contributors: Yunjiang Qiu, David U Gorkin, and Bing Ren**): Hi-C reads were aligned to the GRCh38 reference genome using BWA-MEM with default parameters[2] . Hi-C reads are paired-ended, but we align each read end to the reference genome independently because standard paired-end mapping algorithms are not designed to handle the large distances that separate Hi-C read pairs. After mapping, we performed several filtering steps:

1) Hi-C reads may span a ligation junction, in which case two parts of the read may map to two different regions of the reference. BWA-MEM handles these "chimeric" reads by outputting two different alignments – one for each part of the read. For Hi-C data we are interested specifically in the alignment of the 5' portion of the read (i.e. 5' to the ligation junction), because the region 3' to the ligation junction will be captured by the other read in the pair. Thus, we filtered the BWA-MEM output to keep only the 5' alignment when split alignments were reported. For similar reasons, if the 5' portion of a read did not align, that read was discarded.

2) Low quality alignments were removed (MAPQ < 10).

3) Read ends were re-paired, and any only pairs in which both read ends passed all filters were kept for downstream analysis.

4) PCR duplicates were removed with Picard. Phasing was then performed using the Haploseq pipeline as previously reported [18].

Briefly, aligned read pairs were realigned and recalibrated using GATK[19]. The badmate parameter was disabled to keep long range read pairs. Hapcut was then used to perform phasing[20]. Haploseq modifies Hapcut by calculating the probability that read pairs come from different chromosome homologs based on insert size, and then adjusting the base quality alignment scores to account for this probability. In almost all cases Hapcut generated one haplotype block per chromosome, spanning both arms of the chromosome. One exception is chromosome X in HG00513, for which Hapcut reported separate haplotype blocks for each arm of the chromosome, reflecting an inability to reliably phase the two arms relative to each other. For chromosome 1 and chromosome 9 in all individuals, each chromosome arm was phased separately. These two chromosomes have exceptionally large centromeric repeat arrays, and thus Haploseq cannot reliably phase the chromosome arms relative to each other at the sequencing depth obtained in this study.

## Transcriptome Sequencing

**Library preparation and Sequencing (Contributors: Talkowski, M):** Total RNA was extracted from EBV-transformed lymphoblastoid cell lines (LCLs) using TRIzol® (15596026, Thermo Fisher Scientific Waltham, MA, USA) from cell pallets according to manufacturer's instructions. In brief, cell pellets (containing between 1-5e6 cells) were homogenized in TRIzol® reagent, followed by chloroform addition and phase separation. RNA was precipitated from aqueous phase using isopropanol followed by washing the RNA pellet with 75% ethanol. RNA pellet was suspended in RNase-free water and stored at -80□C. All nine strand-specific RNAseq libraries were prepared using the Illumina TruSeq kit (Illumina, San Diego, CA, USA) according to manufacturer's instructions, as described[21,22]. One microliter of diluted (1:100) External RNA Controls Consortium (ERCC) Spike-in Mix (4456740, ThermoFisher) containing 92 synthetic RNA standards of known concentrations and sequence was added to each RNA-sequencing library alternating between mix1 and mix2 for each well in batch to estimate the detectable expression abundance thresholds. PolyA bead capture was used to enrich for mRNA, followed by stranded reverse transcription and chemical shearing to make appropriate stranded cDNA inserts for library. Libraries were completed by addition of sample specific barcodes and adapters for Illumina sequencing followed by 10 cycles of PCR amplification. Final concentration and size distribution of libraries were evaluated by Agilent 2200 TapeStation (Agilent, Santa Clara, CA, USA) and/or qPCR, using Library Quantification Kit (KK4854, Kapa Biosystems, Wilmington, MA, USA), and multiplexed by pooling equimolar amounts of each library prior to sequencing. Libraries were multiplexed, pooled and sequenced on multiple lanes of an Illumina HiSeq2500, generating an average of 67.5 million paired-end reads of 51 bp per sample.

**Sequence data processing:** Further quality control of sequence reads was assessed by fastQC[4] (v.0.10.1). Subsequently, sequence reads were aligned to human reference genome Ensembl GRCh38 (v.81) using GSNAP[23] (v. 06-23-2015) with options –N 1 –B 3 --quality-unk-mismatch=1. Further quality

control of alignments was assessed by a custom script utilizing Picard Tools (http://picard.sourceforge.net) RNASeQC [24], RSeQC [25] and SamTools [8] Gene level counts were tabulated using BedTools's multibamcov algorithm [26] (v. 2.17.0) on unique alignments for each library relying on Ensembl gene annotation [27] (GRCh38 v.81). Analysis of ERCC spike-ins as described in Blumenthal et al.[21] estimated the expression threshold for detection to be at least three mapped reads.

## 1.2 Data availability

**Data coordination and access (Contributors: Fairley, Clarke, Zheng, Lowy and Flicek):** The International Genome Sample Resource (IGSR) is working with the HGSVC to assist with data coordination, analysis and distribution. The data collected by the HGSVC, including raw data and Illumina alignments, is available via an FTP site (ftp://ftp.1000genomes.ebi.ac.uk/vol1/ftp/data_collections/hgsv_sv_discovery/), can be browsed through the data portal (http://www.internationalgenome.org/data-portal/data-collection/structural-variation) (**Supplementary Figure 26**) and used under the Ft. Lauderdale principles for data reuse and publication https://github.com/igsr/1000Genomes_data_indexes/blob/master/data_collections/hgsv_sv_discovery/README_hgsvc_datareuse_statement.md.

**Data sets used by the HGSVC:** As noted, data from the HGSVC can be found at this URL: ftp://ftp.1000genomes.ebi.ac.uk/vol1/ftp/data_collections/hgsv_sv_discovery/. Raw data (and Illumina alignments) are listed in the .index files in this directory, with the exception of the Bionano data, which is at this location: ftp://ftp.1000genomes.ebi.ac.uk/vol1/ftp/data_collections/hgsv_sv_discovery/working/20160307_bionano_optical_maps_90x/data/. In addition, reference files used more widely in IGSR, such as the reference genome, are also available from the wider IGSR FTP site. A summary table can be found in **Supplementary Data 49**.

# 2. Chromosomal haplotype resolution and integration

We first aimed to build dense and accurate chromosome-length haplotypes on single-nucleotide variants (SNVs). These haplotypes can then act as a backbone to add further variation to and facilitate a comprehensive comparison across platforms. To establish basic properties of the haplotype information delivered by different platforms, we first created haplotypes from each technology individually. To this end, we used high-coverage Illumina paired-end data and ran FreeBayes (v1.0.2) and the GATK HaplotypeCaller (v3.5-0-g36282e4). We filtered the calls (FreeBayes: QUAL>=30, GATK: QUAL>=200) and retained those bi-allelic SNV calls where the three genotypes for all samples from a family agree between the two callers.

## 2.1 Mapping of meiotic recombination events

**(Contributors: David Porubsky)**

Global chromosome-length haplotypes assembled using Strand-seq can be used to map meiotic recombination events within a single family trio. We mapped all meiotic recombination events in every family trio solely based on Strand-seq (**Supplementary Figure 27**; **Supplementary Data 50**). As expected we observed higher number of meiotic recombination events occurring on the maternal homologues[28–31]. This difference was the most prominent in Yoruban trio and the least for the Puerto Rican trio (**Supplementary Figure 27**). Using Strand-seq data only we achieved high resolution maps of meiotic recombination with median resolution less than 25 Kb. We set to further refine these maps using PacBio reads and WhatsHap. The majority (~70%) of mapped meiotic breakpoints overlapped with breakpoint estimates obtained from PacBio reads and allowed us to further refine the mapped meiotic breakpoints (**Supplementary Figure 28**; **Supplementary Data 50**) Notably, with this approach we managed to map

meiotic recombination breakpoints with unprecedented resolution (median ~1.5 Kb). In addition, all but one PacBio refined breakpoints were mapped at maximal possible resolution between two heterozygous SNVs. The majority of Strand-seq mapped meiotic recombinations that were not refined by PacBio could be in theory refined further using residual SNVs present in such meiotic recombinations breakpoints.

We have further explored meiotic recombination breakpoints refined by PacBio reads (in total 162 breakpoints from all trios) to search for enrichment of previously reported elements, such as Alu. L2, THE1A and THE1B at sites specific for human recombination hot spots[39]. In line with the previous research[39], we have found significant enrichment of Alu (especially AluS) and L2 elements around our meiotic breakpoints (**Supplementary Figure 29**). However, enrichment for Alu and AluS elements was significant only when mere occurrence of these elements within a meiotic breakpoint was considered. This might be an artefact of our randomization process given the widespread distribution of Alu elements across the genome. (**Supplementary Figure 29, a-c right column**).

## 2.2 Strand-seq phasing

To assemble genome-wide haplotypes exclusively from Strand-seq data we have used R package called StrandPhaseR. StrandPhaseR takes as an input aligned BAM (binary alignment map) files from single cell libraries that were pre-selected based on the following quality criteria. BAM files were filtered for duplicate reads, alternative alignments and low mapping quality reads (mapq < 10). Haplotype informative WC (Watson-Crick) regions were localized in every single cell as was done previously[13]. To phase such WC regions across all single cells we used our latest phasing pipeline called StrandPhaseR[32]. List of single nucleotide heterozygous position suitable for phasing were obtained from 10x Genomics variant calls. Variable positions covered with bases of quality less than 20 were filtered out. For each individual, final haplotypes were exported as a single VCF file, separately for each chromosome.

## 2.3 Mapping of meiotic recombination events using Strand-seq

To map meiotic recombination event in each family trio we have first assemble genome-wide haplotypes independently for each individual in a trio using solely Strand-seq data[13]. Next we performed a pairwise comparison of each child's homologue to both, maternal and paternal homologues. This identified the heterozygous positions that distinguished the child from each parent and such positions were used to assign the parental identity to each child’s homologue. Every comparison was encoded as a vector of zeros and ones based on the parental homologue to which child's homologue correspond. (zero – parental homologue 1, one – parental homologue 2). Then a circular binary segmentation algorithm (R package fastseg, minSeg set to 100)[33] was applied on such binary vectors. Segments smaller than 5 Mb and segments overlapping with homozygous inversions were filtered out. Meiotic breakpoints were localized as the end position of one segment and start position of the following segment. All localized meiotic breakpoints were further assessed and confirmed by eye.

Meiotic recombination breakpoints were independently predicted using pedMEC algorithm implemented in the WhatsHap[34]. PacBio reads from the whole trio along with human meiotic recombination rates were used to estimate most likely point of meiotic recombination. Little number of overlapping breakpoints were merged together leaving us with a few hundreds of predicted breakpoints. To exclude false positives, we considered meiotic breakpoints mapped using Strand-seq as a gold-standard. Therefore, only PacBio predicted breakpoints that overlapped with or were in 50 Kb distance from Strand-seq meiotic breakpoint were considered for further analysis. For such ranges we reported refined breakpoint position using PacBio reads. Lastly we searched for meiotic breakpoints with a number of residual SNVs within the breakpoint. Then in turn we have established a theoretical resolution of such breakpoint as a maximal distance between subsequent residual SNVs within such breakpoint.

# 3. Full-spectrum variation detection

## 3.1 Short insertion and deletion (<50bp)

**(Contributed by: Ye, Guo)**

Current methods for indel discovery using short-read sequence data are thought to underestimate by as much as 40% the true number of events [35,36]. This effect is especially pronounced for events greater than 10 bp in length. In order to provide a more comprehensive map of human genetic variation from SNVs to large complex SVs we therefore analyzed the three trios for the presence of indels define here as insertions and deletions ranging from 1bp to 49 bp.

### Merging of Illumina callsets

Indels from three Illumina callsets: Pindel[37], GATK[38] and FreeBayes[39] were merged. The merged region is between 1 and 49 bp. To create a deletion and insertion merged set, calls in each callset were separated into deletions and insertions. For CHS, Pindel, GATK and FreeBayes detect 551417, 305509, 160381 deletions and 452390, 275998, 148707 insertions respectively. For PUR, Pindel, GATK and FreeBayes detect 562056, 312359, 169348 deletions and 459026, 282492, 154248 insertions respectively. For YRI, Pindel, GATK and FreeBayes detect 564226, 393464, 218569 deletions and 456768, 354097, 187615 insertions respectively. A merged deletion and a merged insertion set were created by merging Pindel, GATK and FreeBayes tabix indexed set (tabix version 0.2.6) using vcf-merge function of vcftools [40], version 0.1.13) with default options. The merged deletion and insertion Illumina set contain 1,166,979 and 1,077,222 calls respectively. Size distribution of deletions and insertions in individual set and merged set is shown in **Supplementary Figure 30A** (Deletion) and **Supplementary Figure 30B** (Insertion).

### PacBio indels

**(Contributed by: Chaisson, M)**

To avoid artifacts of merging indels from multiple assemblies, we called indels from the Phased-SV haplotigs only. Indels were detected in the regions of haplotigs that were realigned during SV calling, and from the haplotig to reference alignments otherwise. We ignored all single base indels and homopolymer indels less than 6 bases. Indels were maximally left-aligned using vt normalize. A final set of pacbio calls was generated by removing any call less than 10 bases if there are no Illumina reads with a similar sized (50%) indel within 50 bases.

### Platform comparisons

**(Contributed by: Ye, Guo)**

We next compared the merged Illumina callset with indel calls generated the unified PacBio callset and unified Phased-SV/MsPAC (PS/MP) call set (below). PacBio Phased-SV calls from HG00514 were used to compare with the merged Illumina callset up to 1 Kb. Similarly, PS/MP calls were also divided into deletions (449,146) and insertions (384,358). The size distribution of PS/MP PacBio and Illumina merged set was shown in **Supplementary Figure 30A** (Deletion) and B (Insertion). The contribution of three Illumina and the PacBio callsets to the Illumina-PacBio integrated set was shown in Venn diagrams (**Supplementary Figure 30C**) created by overlapping the four callsets using vcf-compare [40]. Additionally, an overlap between Illumina merged set and UW-MSSM PacBio callset was conducted. BED files were created from VCF files of the three callsets by padding SV lengths to breakpoints and then compared using bedtools intersect (version 2.26.0), with a 50% reciprocal overlap. The results were shown in **Supplementary Figure 30D** On the size frequency plots of deletions and insertions, 2n peaks are clearly visible, probably due to the higher mutation rate in microsatellite repeats. The numbers of calls for indels smaller than 30bp are comparable between Illumina merged set and UW-MSSM PacBio callset while the sensitivity of the

latter remains after Illumina merged set lost detection power, especially for insertions. The ratio of number of calls detected by the Illumina based methods relative to the number of calls by the PacBio based methods is given in **Supplementary Figure 31**.

### Repeat analysis

The BED files of deletion and insertion calls in Illumina merged set, Phased-SV set, and HySA were overlapped with different repeatMask bed files using bedtools intersect (version 2.26.0), looking for deletions and insertions fully residing in a repeat (-f 1.0). The repeat overlapped calls , converted to frequencies, were summarized **Supplementary Figure 30F,** where each color bar represents a fraction of deletion/insertion calls overlapped with each repeat class. "Genome" represents the background fractions of each repeat class in human genome. Simple repeats are enriched for short indels and all three sets (Phased-SV callset, HySa and Illumina merged set) have alleviated proportion of simple repeats, even though the pacbio related sets have significantly higher percentages.

In order to not inflate indel counts due to different alignment parameters, several steps were taken during merging the PacBio and Illumina calls. The PacBio and Illumina indel callsets were merged by selecting all Illumina calls, then any pacbio call not within 100bps of an Illumina call. Any PacBio calls within 100bps of an Illumina call are assigned the Illumina position, but retain the PacBio genotype. All remaining PacBio calls were added.

## 3.2 Haplotype-resolved SV characterization and integration

### Comparison of alignment methods in tandem repeat loci

The majority of SV clusters (95%) are within tandem repeat loci (**Supplementary Data 51**). The presence of multiple SVs at a tandem repeat locus may be due to either alignment fragmentation (false positive), or different domains of the tandem repeat expanding and contracting independently. The latter is known to happen and is related to the purity of the tandem repeat monomer.

To investigate the source of the clusters of SVs at tandem repeat loci, the loci were visualized by generating the dot plot of the haplotype sequence versus the reference. The BLASR alignments were plotted on top of each dot plot. For comparison, we additionally included the NGM-LR alignments for the same loci. Roughly 4000 dot plots were generated per haplotype. Because these images are useful for understanding the diversity of these tandem repeat sequences, we are distributing them through the 1000 genomes file server at:

http://ftp.1000genomes.ebi.ac.uk/vol1/ftp/data_collections/hgsv_sv_discovery/working/20180515_Dotplots_TandemRepeats/

Because the SVs detected in a tandem repeat region is dependent on the gap parameters used in the alignment, this analysis also enabled the comparison of the two different alignment methods: BLASR and NGM-LR. The BLASR method defines alignments based on sparse dynamic programming and thus favors alignments with exact matches. The NGM-LR method incorporates a double-affine gap parameter that allows larger gaps and has been shown to increase sensitivity particularly with lower coverage sequencing projects. Considering only calls within tandem repeat loci in HG00514 as an example, 54% lof loci have the same number of calls generated by each method (**Supplementary Data 52**). A comparison of the two methods is shown in **Supplementary Figure 32.** There is an excess of loci at which BLASR generates more calls than NGM-LR (36% versus 9.5%). Examples of these loci are given in **Supplementary Figure 33.**

Manually inspecting a set of the BLASR alignments revealed that many instances where there is an SV cluster, the alignment could be shuffled so that one SV could be replaced by an SNV with a mismatch between monomer sequences. However, the most likely mechanism of variation in tandem repeats is

through change in copy number of repeat monomers and is thought to happen orders of magnitude more frequently than SNV variation. While we emphasize that it is a challenging problem to determine an alignment of large tandem repeat sequences that reflects the exact edits that have happened since the two genomes shared a common ancestor, we postulate that the more biologically accurate alignments favor insertions and deletions.

The number of SNVs determined by each alignment method was compared at all SV loci. There is an increase in the number of loci where the NGM-LR method detects more SNVs than BLASR (**Supplementary Figure 34**).

To investigate the relationship between SNV count and SVs, TR loci were partitioned according to which method detected more SNVs than the other, or when the number of SNVs was the same. When the number of SVs was the same, or when BLASR detected more SNVs than NGM-LR, there was generally greater agreement between the two for the number of SVs at the locus, with the exception of when there were a large number of SVs (> 5 SVs) at a locus **(Supplementary Figure 35**). Because of this, we opted to not encode the individual SVs at these loci, and instead represent the entire sequence of the tandem repeat locus as an alternate haplotype.

### Comparison of SV distribution and genomic regions

**Structural variation with respect to segmental duplications.** To assess whether there was an increase of SV around regions of high segmental duplication content, we performed a simple visual comparison of SD content of the merged Phased-SV and MS-PAC callsets with segmental duplications. We calculated the number of segmentally duplicated bases and SV counts per 100 kbp. The largest spatial effect was an increase of SV near telomeres. Other regions of high segmental duplication were not associated with a large increase in SV, as shown in **Supplementary Figure 36.**

### Structural variation by genomic region.

The variation in different types of the genome was counted for telomeric, tandem repeat, and not-repetitive DNA, as well as for general categories of mobile element and coding sequence. The results are summarized in **Supplementary Data 53.**

### Intersection with 1000 Genomes Phase 3 Callsets

**(Contributed by: Audano)**

We compared SVs to a previously published set by Sudmant et. al[41] as part of the 1000 Genomes phase 3 project. SVs were separated into insertions, deletions, and inversions. Copy-number variants (CNVs) other than deletions were removed. HGSVC SV calls were intersected with this set using a 50% reciprocal overlap for each variant type. For this analysis, variants were not filtered based on genomic regions.

### Proximity of Integrated PacBio calls to 1000 Genomes SV

We counted HGSVC variants located distally from variants in the 1000 Genomes call set. Three sets of regions were computed taking the reference bases affected by each 1000 Genomes variant, adding 1 Kb, 5 Kb, and 10 Kb upstream and downstream, and merging the regions. HGSVC SV calls that did not affect these reference bases were counted. This analysis was completed independently on insertions, deletions, and inversions so that only variants of the same type in the HGSVC calls and 1000 Genomes calls were compared. Approximately 80% of variants in this callset are not within 1 Kb of 1000 Genomes variants (81% HG00514/HG00733, 80% NA19240).

### Orthogonal support of PacBio variants

**Illumina genotyping (contributed by Audano):** We used SMRT-SV Genotyper (Huddleston et al., 2017) to map Illumina WGS reads from the child genomes to the SV calls. The SMRT-SV genotyper uses local

assembly contigs aligned to the reference, and this data was available for SVs called by Phased-SV. We genotyped these SVs against 24 population samples (1000 Genomes high-coverage, PCR-free samples) and against Illumina WGS data from the sample itself. We analyzed how well SV calls from a sample genotyped against Illumina data for itself. To restrict this analysis to SVs that are amenable to the genotyping approach, we restricted this analysis to SVs with a no-call rate of 80% or less over the population samples. For those variants, we determined what proportion of them support the SV call .

For all variants with a no-call rate of 80% or less, 92% to 96% of the SV calls were supported by the genotyper. The genotyping approach relies on ALT-aware sequence read alignments to the reference and alternate contigs. Since short-read alignments are known to be unreliable in repetitive and duplicated sequence, we repeated this analysis outside of annotated tandem repeat and segmental duplication regions. In this set, we find support for 96% to 98% of the SV calls.

**Oxford Nanopore validation (contributed by Chaisson).** As a second genome-wide approach, we generated 18.9 fold sequence coverage for one of the samples (HG00733) using Oxford Nanopore Technologies' GridION X5 (ONT)using FLO-MIN106 (R9.4) flowcells for 48 hours. The read length and coverage distributions are shown in **Supplementary Figure 37**.

We opted to perform validation of SV calls by searching for support for individual SV calls within the ONT reads rather than by creating a new SV callset from this data. The ONT reads were mapped using BLASR, merging gaps between matches under 100 bases until merged matches are at least 100 bases in order to reduce alignment based fragmentation of SVs during noisy read alignment. For comparison, the support from PacBio reads was calculated in a similar manner. All support was stratified by insertion and deletion operations, and inside and outside of tandem repeat sequences in the genome (**Supplementary Figure 38**). Because the overall validation rate depends on read-support cutoff parameters, we first show the distributions of support for each class of event and region for both the ONT and PacBio reads below, by length of SV, and the cumulative distribution of support from each sequencing type. Requiring at least three reads to support an event gives a 91% validation rate for SVs outside of tandem repeats, and between 81 and 85% validation rate for SVs within tandem repeats.

An important consideration with ONT data is that base calling is highly dependent on parameters especially within regions of tandem repeats. Depending on parameters, certain sequences can be entirely absent from a nanopore read. For example, we examined a region (chr1:37442000-37447000) where there were two tandem repeats, one (5') which the sample matched the reference, and another (3') tandem repeat where our callset contains an insertion SV. With default basecalling with the ONT reads, most reads had a poorly defined deletion over the 5' tandem repeat, and did not show the insertion in the 3' tandem repeat. When different parameters are used for base calling the ONT reads (dna_r9.4_450bps.cfg -–chunk_size 20000 —runners 3 –x 'auto'), the sequences become consistent with our callset. An example of raw read alignments for different basecalling parameters and PB reads is shown in **Supplementary Figure 39**.

**Agreement of SV with BAC sequencing.**

There were 57 BACs that were sequenced in order to assess the base pair accuracy of our *de novo* assemblies. We looked for SVs that were in the BACs that overlapped with our callset. There were 27 homozygous calls that overlapped with the regions sequenced by BACs, of which 25 had overlapping calls in the BACs (92%). Of the two missed calls, one showed strong heterozygous support, and the other was in a 1500 base tandem repeat, and the BAC supported an SV 616 bases away. Of the heterozygous calls, 22 out of 39 overlap with an SV detected in the BACs (56%) show support, in agreement with the BACs only representing a single haplotype. When restricting to 50% reciprocal overlap, 22 out of the 27 homozygous calls (81%) and 15 of 39 heterozygous calls (38%) match, although our experience merging

datasets shows that a 50% reciprocal is an under-estimate of the agreement between methods, particularly in tandem repeats.

**PacBio Inheritance (Contributed by: Chaisson):** To determine inheritance status of each SV, we counted read support for the unified PacBio callset using the PBRC method with the PacBio parental genomes. A minimum of one read was required to consider a variant present in a parent. We confirmed on average 8,881 of 9,348 (95.0%) homozygous calls as present in both parents, and an average of 15,278/15,890 (96.1%) heterozygous calls as present in one parent. When considering only events that lie outside of tandem repeats, 95.0% of homozygous calls are confirmed in both parents, and 98.3% of heterozygous calls can be confirmed in at least one parent.

In order to make this assessment independent, we regenerated Phased-SV assemblies without parental reads, and then checked for Mendelian concordance with the parental reads. The average assembly contiguity decreased dramatically, highlighting the need to use at least 30X coverage for the local assembly based approach. Additionally, the number of SV calls decreased, indicating that the local assembly approach has decreased coverage over SVs. The mendelian concordance based on this assessment, nevertheless, remained high between 84-88.3%.

**Bionano Genomics Support of Unified PacBio Calls (Contributed by: Chaisson):** We surveyed the concordance between the Bionano Genomics (BNG) SV calls and the unified PacBio callset with less stringent filtering ($f_{BN}$ = 0.25) to allow for a greater number of overlaps. Because a BNG call may overlap multiple PacBio SVs, we selected the PacBio SV with the lowest $f_{BN}$ for an overlap. On average, 69.7% of PacBio SVs ≥ 1 Kb were validated by a BNG SV.

**GRAPHITE Illumina Assessment of PacBio SVs (Contributed by: Marth, Lee, D):** Additional support for variants was detected using the GRAPHITE (https://github.com/dillonl/graphite) and which takes advantage of the alternate reference alleles provided in the integrated pacbio callset. In this approach, a local 'variant graph' in the region of one or more candidate variants is constructed by adding the candidate alleles as branches in a reference graph, initialized with the reference allele as its first branch. The primary sequencing reads are then aligned directly to this graph, using our implementation of the partial order alignment algorithm [42]. This is able to recover previously poorly mapped or unmapped reads from a sample by mapping against a path including the alternate allele carried by that individual. With this approach, one can 'adjudicate' a candidate variant i.e. confirm or reject its presence in a given sample based on the presence/absence of read mappings supporting the candidate alternate allele. Here we applied GRAPHITE to cross-validate candidate variants called from PacBio long-reads with Illumina data. We accomplished this by constructing a local variant graph in each region where a PacBio candidate variant, or multiple overlapping variants, were called, and re-mapping all Illumina reads (including unmapped mates) from the same sample to this graph. PacBio variants were annotated according to the number of Illumina reads they received. Variants for which the number of confirmatory Illumina reads exceeded a threshold (5) were annotated as 'adjudicated'.

## Orthogonal sequencing approaches

**Bionano Genomics Analysis (Contributed by: Pang & Hastie):** Bionano Genomics optical mapping was used as an independent de novo assembly and SV calling approach that leverages extremely long (150 Kb to 2 Mb) single molecules for whole genome *de novo* assembly. *De novo* assemblies can be used to detect insertions, deletions, inversions, repeat expansions, translocations and transpositions. Bionano optical mapping produces assemblies that are sensitive to heterozygosity and are able to produce two alleles across the majority of the genome.

Automated insertion and deletion calling has been used here and has identified approximately 5000 SVs for each enzyme, counting homozygous SVs twice and calling the same SV with each enzyme in most cases. Generating de novo assemblies and SV calls with two enzymes is valuable in improving confidence in SVs called with both enzyme and by covering assembly gaps in one enzyme with the complementary enzyme. In order to reduce the redundancy, SVs that are homozygous are collapsed and SVs from two different enzymes that have the same reference position and have similar sizes are merged.

**Bionano *de novo* assembly**: *De Novo* assembly was performed using Bionano's custom assembler software. *De Novo* assemblies of all individuals in the CHS, PUR and YRI trios were performed with IrysSolve v2.3 software between February and March of 2016. For comprehensiveness, probands were re-assembled with IrysSolve v2.5.1 between October and December of 2016. Pair-wise comparison of all DNA molecules was done to create a layout overlap graph, which was then used to create the initial consensus genome maps. By re-aligning molecules to the genome maps *(P Value $10^{-11}$)* and by using only the best match molecules, a refinement step was done to refine the label positions on the genome maps and to remove chimeric joins. Next, during an extension step, the software aligned molecules to genome maps (*P Value $10^{-11}$*), and extended the maps based on the molecules aligning beyond the ends. Overlapping genome maps were then merged using a *P Value cutoff of $10^{-15}$.* These extension and merge steps were repeated five times before a final refinement was applied to "finish" all genome maps *(P Value $10^{-11}$)*. Two assemblies were constructed, one for each nickase.

Using IrysSolve v2.5.1, during the extension step, the software identifies clusters of molecules that aligned to genome maps with end alignment gaps of size > 30 Kb (i.e. over 30 Kb of one side of the molecules did not align), these molecules were split from the map and re-assembled. In addition, for both IrysSolve v2.3 and IrysSolve v2.5.1, the final refinement step searched for clusters of molecules aligned to genome maps with internal alignment gap of size < 50 Kb, in which case, the genome maps were converted into two haplotype maps. The extend-and-split function is essential to identify large allelic differences and to assemble across loci with segmental duplications, whereas the refinement haplotype function can find smaller differences.

**Bionano structural variation:** SV was called based on the alignment profiles between the *de novo* assembled genome maps against the public human reference assembly GRCh38. We required an alignment cutoff of P-Value of $10^{-12}$ to identify the best aligned locations for any given match group within a genome map. SV calling was done for the Nt.BspQI and Nb.BssSI assemblies independently. Significant discrepancies in the distance or the number of unaligned labels between adjacent aligned labels (*outlier P-Value $3x10^{-3}$*) would indicate the presence of insertion and deletions. Genome maps whose alignments were in opposite orientations would indicate the presence of inversion breakpoints.

Finally, insertions and deletions captured by each of the single-enzyme assemblies (Nt.BspQI and Nb.BssSI) were compared and merged into a final SV call set. Insertions and deletions that were within 10 Kb and with over 80 % reciprocal size similarity were merged together, and the innermost breakpoints were recorded as the merged variant breakpoints. To minimize false positives, we removed calls whose size was less than 500 bp, calls found by single nickase assembly but with a variant confidence score of < 0.5, or calls found by both nickases but with a confidence of < 0.3. No merge was performed for inversion breakpoints.

## 3.3 Variation detected by short-read sequencing

### SV Detection Methodologies on Illumina Genome Sequencing

An ensemble of Illumina structural variant callers (Genome STRiP, GATK, Pindel, MELT, DELLY, LUMPY, WhamG, dCGH, SVelter, Manta, forestSV, Holmes, TARDIS) and PacBio/Illumina hybrid methods (HySA & cloudSV (unpublished)) were analyzed to complement the PacBio only SV calls. Unlike previous efforts

(phase III), the false discovery rate was not strictly controlled for the Illumina callers allowing, for the first time, a broad comparison of SV callers, vs, a highly accurate PacBio callset.

**WHAMG (Contributed by: Kronenberg, Z)**: A sensitive and specific call set were generated for whamG[43]. In both datasets we jointly called the three trios and filtered with a modified version of "filtWhamG.pl", removing events that have low support, low scoring "SVTYPE" classification and events with a high fraction of mate-pairs cross chromosome mapping. All calls across the three trios were merged into a single VCF with "mergeSVcallers" (50% reciprocal overlap and same "SVTYPE"). The merged calls were genotyped with SVTyper[44]. For the specific set we applied "annotate_hq.py" a script that requires no unknown genotypes, at least one heterozygous or homozygous alternative genotype call, a quality score above 100 and a median genotype quality above 100. The filtering script are in the whamG repository and the FTP. The SV counts and types for both the sensitive and specific set are listed in **Supplementary Data 54**. The sensitive set was used for Illumina integration.

**LUMPY (Contributed by: Kronenberg, Z):** Split- and discordant- reads were extracted from the BAM files using a custom program ("filter.c"). Each trio was jointly called with Lumpy [45]. BND/translocation events were filtered for all analyses. The trios were merged and genotyped using the same methodology outlined in the WhamG section, with the exception of the whamG specific filtering procedure. The number and type of SVs obtained are enumerated **Supplementary Data 54**. The sensitive callset set was used for Illumina integration.

**DELLY (Contributed by: Rausch, Korbel):** The germline SV calling workflow of Delly v.0.7.5[46] was used to call large structural variants >500bp and small InDels in the size range of 15bp-500bp with default parameters in the deep coverage Illumina paired-end data. Candidate germline SV sites were called sample by sample and all identified SV sites were concatenated into a single SV site list using delly merge with a reciprocal overlap of 0.5 and a maximum breakpoint offset of 500bp. All candidate SV sites were re-genotyped using delly on all trio samples. The output BCF files were merged into a single BCF file that contained all candidate SVs and their respective genotypes across the 9 trio samples. Uncertain genotypes with phred-scaled genotype quality below 20 were set to missing using VCFaid (https://github.com/tobiasrausch/vcfaid) and sites with an overall genotype missing rate >25% in all 9 samples were dropped. It was also required that at least one sample shows clear evidence for the SV with at least 20% of the reads confirming the alternative allele at a given SV locus.

To ensure high specificity we further filter the remaining SVs using a machine learning approach that uses site and genotype properties collected from svprops (https://github.com/dellytools/svprops). The SV classifier was trained and validated on a subset of likely true and false SVs that was derived from three sources. The first source were SVs that could be re-genotyped by delly in the 1000 Genomes low coverage data and were the IRS method (Sudmant, 2015) could assign a p-value. This set was then stratified into likely true SVs (p-value < 0.5) and likely false SVs (p-value >= 0.5). The second training source were likely false SVs that showed Mendel transmission errors. The third training source was a set of 100 randomly picked SV sites that we manually inspected using IGV (http://software.broadinstitute.org/software/igv/) and then classified as likely true or false SVs. All training sites were split into a training and validation set and machine learning parameters were picked to derive a final SV site list of an estimated FDR of 5% using the validation set. In addition, Delly v0.7.5 was used to discover and genotype a small set of confident complex SVs as described previously [41]. These complex SVs give rise to 2 overlapping paired-end clusters, which are then classified into simple inversions, inversion with an adjacent deletion and proximal inverted & non-inverted duplications using the delly dpe (double paired-end signatures) subcommand. For inversions, we in addition screened the 7 Kb jumping libraries using delly v0.7.5 and also required double paired-end support for the filtered set.

Overall, Delly ascertained 11,823 deletions, 5,315 insertions, 173 tandem duplications, 16 inversions, 15 inversions with an adjacent deletion, 40 proximal non-inverted duplications and 49 proximal inverted duplications in the 9 trio samples. Independent of the machine learning training and validation set we also applied IRS on the 9 trio samples using CytoScan Arrays.This analysis yielded an estimated FDR of the final deletion set of 2.8%.

**dCGH (Contributed by: Nelson, Kronenberg, Eichler):** Illumina WGS sequence data were mapped to a repeat masked version of the human reference genome (GRCh38) using the mrsFAST sequence aligner as previously described [47] and variants were called using the digital comparative genomic hybridization (dCGH) method [48]. Deletion and duplications (>1 Kb) were identified by comparison to a diversity panel of 17 Simons Genome Diversity Panel (SGDP) human genomes (**Supplementary Data 55**).

**Genome STRiP (Contributed by: Handsaker, B)**: Genotyped copy number polymorphisms were called using Genome STRiP [49] version r2.00.1691, which contains support for bwa alt-aware alignments, and using the reference metadata for GRCh38 (12Oct2016 version). Calls were made using both the Genome STRiP deletion pipeline and CNV pipeline and then filtered and merged as described below. Genome STRiP is a population based calling method, designed to run on hundreds or thousands of samples. The small cohort size (9 individuals) necessitated atypically stringent filtering and resulted in decreased sensitivity.

Deletion pipeline: Raw calls were generated using default settings with two exceptions:

- The parity correction threshold was reduced (-P depth.parityCorrectionThreshold: 0.1) to accommodate the small number of related samples.
- The default filtering on inbreeding coefficient in the genotyping step was disabled, to account for the small number of related samples.

Raw calls from the deletion pipeline were filtered using the following criteria:

- All default filters with the exception of INBREEDINGCOEFF (inbreeding coefficient).
- Sites were retained if they had GSCNQUAL >= 1 and GSCLUSTERSEP >= 3.
- Sites overlapping known VDJ recombination regions were excluded.
- Sites were included for the autosome and chromosome X only.

CNV pipeline: Raw calls were generated using default settings with two exceptions:

- The parity correction threshold was reduced (-P depth.parityCorrectionThreshold: 0.25) during discovery.
- The parity correction threshold was reduced (-P depth.parityCorrectionThreshold: 0.1) during subsequent genotyping and merging.

Raw calls from the CNV pipeline were filtered using the following criteria:

- Site call rate of 100%, with at least one sample called non-reference at 95% confidence.
- Multi-allelic sites predicted to have more than 3 observed alleles were excluded.
- Sites where any individual was called at copy number 9 or greater were excluded.
- Sites with deletion alleles that were shorter than 1500bp were excluded.
- Sites with only duplication alleles that were shorter than 4000bp were excluded.

- Sites were included for the autosome and chromosome X only.

Merging: Redundant calls were removed using the Genome STRiP Redundancy annotator if they had no discordant genotypes and at least 50% reciprocal overlap. When redundant calls originated from both the deletion and CNV pipeline, calls from the deletion pipeline (which are expected to have more accurate boundaries) were retained.

**QC**: To assess call quality, we evaluated all potential Mendelian violations (40) with calls in at least one child (**Supplementary Data 56**). This suggested an initial false discovery rate of 1.3%, predominantly from the CNV pipeline calls. To evaluate the impact of the small cohort, we re-genotyped the sites with potential Mendelian violations in a larger multi-ethnic cohort with 30x whole-genome sequencing data. Manual review suggested that the majority of the potential Mendelian violations were either due to genotyping error, often caused by incorrect boundary determination (Genome STRiP genotyping and boundary assessment are both better-powered in larger cohorts), or were due to the presence of a multiallelic CNV, which can cause the appearance of a Mendelian violation if the full allelic spectrum is not observed. One deletion (chr13:105744780-105747104) appears to be a mosaic de novo deletion in the CHS child. Adjusting for these sites suggests a true site-level FDR of 0.4%.

**Holmes (Contributed by: Mike Talkowski, Ryan Collins, Harrison Brand, Matt Stone, Joseph Glessner):**

**SV Discovery:** The computational analysis pipeline for structural variation (SV) discovery from 3.5 Kb long-insert whole genome sequencing (liWGS) data has been previously described[50,51]. The pipeline, Holmes, requires a sufficiently large cohort of liWGS libraries for simultaneous joint-calling of SV breakpoints and CNV intervals, so we supplemented the nine HGSVC liWGS libraries generated as described above with 91 independent individuals for which we have previously generated liWGS libraries with the same protocol and data processing procedures for an unrelated study (Collins et al. 2017). These 91 supplementary libraries were selected based on a balanced ratio of male and female subjects (50 males & 50 females in final analysis cohort, n=100) and on approximate library and sequencing quality (matched with HGSVC trio libraries on median and median absolute deviation of insert size, haploid physical coverage, chimera rate, and pairwise duplication rate) **(Rodriguez, O. and Bashir, A).** We subsequently performed SV analyses against the GRCh37 reference genome from these 100 individuals. All resulting SV calls were lifted over to hg38 coordinate space with the UCSC liftOver tool requiring a minimum of 50% of the original GRCh37 interval to remap to hg38 coordinates[52]. Based on previous validation experiments using PCR, targeted capture, comparison to chromosomal microarrays, and short-insert sequencing, we estimate an overall true positive rate to be approximately 0.894. Validations were not performed on this call set in the HGSVC samples (**Supplementary Data 57**)
**Results:** Using the median insert (~3.5 Kb) and the physical coverage obtained (mean: 163.9X, range: 144.6X-193.8X), we resolved a mean of 530 large SV per subject (1,270 unique SV sites resolved across all nine subjects) at the resolution of liWGS (SV size ≥ ~5 Kb). We also identified a mean of 53 incompletely resolved SV sites (IRS) and 143 low-confidence CNVs per subject; IRS and low-confidence CNVs were excluded from all subsequent analyses. The remaining high-confidence SV included canonical copy-number variants as well as balanced inversions, insertions, and cxSVs; collectively, complex and balanced SVs represented 25.5% (324/1,270) of all high-confidence SV sites identified in these nine subjects. Notably, we observed a median of 73 canonical inversion variants and nine complex inversions per subject at liWGS resolution. Principal component analysis of a genetic relatedness matrix constructed between these nine individuals showed that the first two principal components clearly cluster all three individuals from each trio. Transmission analysis yielded a mean of 97.1% inheritance across the SV call set. Consistent with previous findings, we detected a mean of 11 large complex SVs per subject in this analysis. The SVs shared between families are given in **Supplementary Data 58**.

**VariationHunter (Contributed by: Hormozdiari): Deletions:** We applied an extension of VariationHunter for calling deletions (>50bp) in the three trios simultaneously[53,54]. Previous versions relied primarily on discordant read mappings for calling deletions. The new version of VariationHunter considers discordant reads, read-depth and split reads signatures from Illumina read-pair data. Split reads are identified from soft-clip data which are remapped as two segments during alignment. For read-depth, we applied a likelihood ratio as metric to filter predicted deletions that resulted from low mapping quality. Finally, we used the tools SVtyper for genotyping all predicted SVs. A total of 4,581 deletions passed genotyping with the calculated IRS FDR of less than 5%.

**Forest SV (Contributed by: Sebat, J):** ForestSV[55] was used to call deletions and duplications in each individual with the default parameters. Adjacent SV calls in the same sample were stitched together if the gap between two calls of the same type was less than 10 Kb. We applied this rule recursively ensuring events consisting of multiple calls were merged. Next, calls were collapsed within families if the reciprocal overlap was at least 90% resulting in 68,944 deletions and 84,281 tandem duplications. Genotypes were then estimated for each SV across all samples using a machine learning genotyper, $SV^2$(https://github.com/dantaki/SV2).

**Manta (Contributed by: Sebat, J):** For each trio Manta[56] was applied with default parameters to predict deletions, tandem duplications, inversions, and insertions. Variants were removed if the length was greater than 15 Mb. Calls with at least 80% reciprocal overlap were merged while reporting the position with the smallest length, resulting in 14,840 deletions, 1,766 tandem duplications, 5,152 insertions, and 1,278 inversions with passing genotyping.

**SVelter (Contributed by Mills, R, Zhao, X) :** We applied SVelter [57] individually to the aligned Illumina sequences for all 9 samples to call simple and complex structural variants >100bp in size. These samples were then merged into a single VCF formatted file. SVs are denoted as follows: DEL (deletion), INV (inversion), TANDUP (tandem duplication), DISDUP (dispersed duplication, includes insertion point in INFO field), DEL_DUP (complex deletion with duplication), DEL_INV (complex deletion with inversion), DUP_INV (complex duplication with inversion), DEL_DUP_INV (complex deletion with duplication and inversion), and OTHER (unclassified). Over the three trios, SVelter reports 13573 (DEL), 506 (INV), 9319 (TANDUP), 3077 (DISDUP), 2165 (DEL_DUP), 369 (DEL_INV), 334 (DUP_INV), and 310 (DEL_DUP_INV).

We applied an in-house long-read validation tool, VaPoR[58] to SVelter predictions using aligned PacBio sequence reads for the trio children and were able to find individual read support for 82% of deletions and 60% of other SV types (both simple and complex).

**Novobreak (Contributed by: Chong, Chen):** NovoBreak v1.1.3[59] was used to detect SVs (>100bp) on the high coverage PCR-free Illumina sequencing data of the three trios (nine samples). NovoBreak is a tool initially designed to discover somatic structural variation in cancer genomes. It applied a *k*-mer targeted local assembly method to detect structural variants in single base resolution. To detect germline SVs in the trios and to meet the interfaces of novoBreak pipeline, each sample in the trios was treated as a ‘tumor’ and a mock ‘normal’ sample without mutations and only allowing sequencing errors (error rate = 0.005) was simulated using wgsim v0.2.3 in SAMtools package[8] from the human reference genome. For each sample, an initial call set was generated using the novoBreak pipeline under the default parameters. The initial call sets were further filtered using a customized script based on the alignment information (split reads, discordant read pairs and mapping qualities) and local assembly information around each SV event. For example, a minimum number of 5 reads for local assembly and a minimum quality of 50 (maximum is 60) were required to generate a high-quality call set. In each trio, the unique calls in the child but not in either parent provided an estimation of the false positive rate (FDR). The estimated FDRs for YRI, CHS

and PUR were 8.8%, 10.7% and 10.3%, respectively. Note that these FDRs were potentially over-estimated since the unique calls in the child may include *de novo* SVs.

Deletions, Duplications and Inversions longer than 100bp were reported for each sample. The numbers of deletions range from 1,883 to 2,717; duplicates from 14 to 30; inversions from 58 to 112. As expected, the African trio YRI had the largest number of SVs due to genetic diversity (~1.4 fold more than those found in CHS or PUR). The diversities of CHS and PUR were at the similar level. The same trend held for all three types of SVs. Overall, the deletion size distribution in each trio followed geometric distribution except that in all trios there was a peak around 300bp, resulting from the Alu element insertions.

**retroCNV (Contributed by: Gerstein):** An extended version of the retroCNV pipeline[60] was used to detect presence/absence polymorphism of processed pseudogenes in all nine individuals independently. The retroCNV pipeline uses discordant read pairs to detect clusters of discordant read pairs evidencing the insertions of exonic or 3'UTR from parent genes. PCR-based validation studies have provided estimates for retroCNV FDRs at 0.1 and below. As an extension of previous version of the pipeline, solo 3'UTR retrotranspositions are also reported.

**MELT (Contributed by: Gardner, E, Devine, S): MELT Illumina call set:** MELT identifies MEIs using signatures of discordant read pairs and split reads that are enriched at sites containing non-reference (non-REF) Alu, L1, SVA, and HERV-K MEIs. PCR-based validation studies and simulations have provided estimates for MELT FDRs less than 5%. MELT detects a wide range of MEI-associated features, including target site duplications (TSDs), precise insertion junctions (where possible), 3' transductions, 5' inversions, size, orientation, interior mutations compared to consensus elements, family/subfamily status, and gene features affected (if any). MELT also calls genotypes for both non-REF MEIs and REF MEIs, thus providing a comprehensive set of polymorphic MEIs in a given genome[61]. MELT only identifies SVs that are precisely caused by mobile element insertion mechanisms, and it does not identify SVs that include MEIs are part of larger events. MELT identified a total of 4,271 MEIs in the three trios, including 3,417 Alus, 531 L1s, 306 SVAs, and 17 HERV-Ks. 99.35% of these calls were consistent with Mendelian inheritance in the trios.

**VariationHunter, Tardis (Contributed by: Hormozdiari, F):** We used the annotated *Alu* and L1 locations in the human reference genome (GRCh38) to guide in the prediction of MEIs. We applied an extension of VariationHunter for MEI prediction[62] called Tardis, which in addition of discordant reads also considers split reads of the reads with soft-clipping. In the MEI call set one *de novo* Alu insertion was predicted with confidence in NA12940 (locus: chr10:128034796-128035846) . The de novo Alu insertion was validated as true de novo using PCR validation and PacBio reads.

Location of VH MEI call sets:

http://ftp.1000genomes.ebi.ac.uk/vol1/ftp/data_collections/hgsv_sv_discovery/working/20160930_pre_ashg_calls/20161011_Tardis_MEI_Calls/

### 3.4 Down - sample analysis of the HGSV Illumina callset

**(Contributed by: Zhao, Talkowski)**

**Method**: Illumina libraries were randomly sub-sampled to 10 iterations of 30X WGS depth, comparable to a standard Illumina genome used in population-based WGS studies, using *samtools* with a unique seed set for each iteration. As it was impractical for all developers to run all algorithms on each down-sampled dataset, a subset of the Illumina SV discovery algorithms that contributed to the largest fraction of calls in the full integration set and could be readily applied by a single naïve user with published documentation,

i.e., Delly, Lumpy, Manta, MELT, Wham, and SVelter, were applied to the sub-sampled libraries with default parameters. These call sets were then analyzed using the Illumina integration script.

**Results**: On average 7,520 SVs were detected in each genome with the selected algorithms on 30X sequences, representing a 34% decrease in sensitivity from a combination of a naïve user running a subset of algorithms and reduced coverage of the 30X genomes. We quantified the relative contribution of these factors and found that a 23% reduction was attributable to excluding a subset of algorithms, while the reduced coverage contributed to an 11% reduction in SV sensitivity compared to the 75X genomes. Among all down-sampled datasets, 78% of SVs overlapped with the full integration set (**Supplementary Figure 40**).

## 4. Integration of Illumina, PacBio, and Bionano callsets

### 4.1 Comparison of read depth and de novo assembly methods

Because a substantial fraction of human genetic variation occurs in regions of segmental duplication [63], and segmental duplications are often missing from *de novo* assemblies[64], we compared the variation detected in regions of segmental duplication through read depth to the segmental duplications that were resolved in the MS-PAC *de novo* assemblies. Because both of the Phased-SV and MS-PAC haplotype partitioned assemblies are biased by the reference, we excluded these assemblies from analysis. The assemblies were mapped to GRCh38 using blasr (www.github.com/mchaisso/blasr ) with parameters "-alignContigs -nproc 16 -minMapQV 30". The UCSC genomicSuperDups annotations for GRCh38 were merged into nonredundant regions, annotating the percent identity of each merged region as the highest annotated identity from all corresponding composite duplications. The boundaries of the alignments of contigs were compared to the boundaries of the merged segmental duplications. All duplications that were covered by at least 50 Kb on both the 5' and 3' end were considered resolved (**Supplementary Data 59**).

The dCGH and Genome STRiP methods both detect copy number variation using read depth, and are sensitive to copy number changes in highly duplicated regions. Between 91.4% and 99.5% of copy number variation detected by dCGH in segmental duplications were in duplications not resolved by *de novo* assembly (**Supplementary Data 60**). Similarly, using Genome STRiP, between 71.3 and 74.5% of copy number variation was in regions of segmental duplication not resolved by *de novo* assembly. These regions are gene rich; 49.2-60.7% of the dCGH and 14.3-15.9% Genome STRiP copy number variants not resolved by assembly overlapped exons.

### 4.2 Genes affecting SVs

Between 77 and 92 genes had at least one exon or UTR affected by a deletion SV, and between 316 and 322 exons affected by insertion SVs. Between 37 and 41% of exon-overlapping deletion SVs were detected in the IL-SV dataset, and between 58 and 70% were detected in the PB-SV dataset. The average length of the PB-SV exclusive exonic SV was between 935 and 1,429 bp, whereas the average length of the IL-SV exon overlapping callset was between 13.2 and 24.7 Kb. This discrepancy indicates a loss of sensitivity in the IL-SV callset for smaller-scale deletion events, consistent with non-exonic SVs. The counts of SVs affecting genes by method and region of gene are shown in **Supplementary Figure 41**.

#### Categories of SV only in PB callset.

The categories of SVs only in the PB callset are shown in **Supplementary Figure 42**. The genes affected by PB only SVs are given in **Supplementary Data 61**.

#### SV overlapping noncoding functional elements

**(Chaisson, Wenger)**

In addition to detecting exons that are disrupted by SV, we looked for potentially functional noncoding elements that overlap SV. We defined 2,857,792 conserved noncoding elements (CE) totalling 121.9 Mb from PhastCons Most Conserved Elements for the hg38-based 100-way multiple alignment from UCSC. Elements within 25bp were merged, and elements overlapping coding elements and UTRs were removed. To avoid spurious alignments in hyper variable regions, alignments were required in a net to chimp in addition to mouse, dog, or horse. We also defined 1,070,532 transcription factor binding sites by lifting over from the USSC HMR Conserved Transcription Factor Binding Sites (http://genome.ucsc.edu/cgi-bin/hgTables?hgta_doMainPage=1&hgta_group=regulation&hgta_track=tfbsConsSites&hgta_table=tfbsConsSites) (TFBS). Nearly half the SV that overlap a conserved element (40.2%) are in large (>2 Kb) SVs (average 15Kb). The SV calls that overlapped TFBS were similarly larger than the average call (37 Kb). This is primarily driven by large BNG events; the average SV excluding BNG was 7.3 kbp for CE 8.3 kbp for TFBS, and The calls unique to PB-SV (3% of calls) were on average 2.2 kbps. While the number of TFBS deletion events unique to PB-SV (missed by IL-SV) was small (21 events/sample), the average size was small; on average 12.7 (~75%) sites per genome were less than 2 Kb (**Supplementary Data 62**).

## 5. Resolving Inversion Sequence Errors in the Human Reference Genome

**(Contributors: Cantsilieris, Eichler)**

During the Strand-seq analysis, we observed 51 regions where the majority of individuals predicted a configuration different from the current human reference genome. We filtered for calls mapping to centromeric and satellite DNA sequences (n=21) and observed that a large fraction of the remaining calls (43%) were almost completely contained within segmental duplications and therefore interpretation was difficult. We specifically focused on regions where at least seven of the nine genomes assessed by strand-seq predicted homozygous inversions and where there was evidence of unique intervening sequence. Such regions suggest that the configuration of the reference genome either represents a minor allele or that the reference (GRCh38) is in error. We identified 17 such regions ranging in size from 3.4 Kb up to 2.9 Mb in size. We selected large-insert clones from human hydatidiform mole source (CHORI-17) and sequence and assembled each region using Canu[65] followed by consensus sequence calling using Quiver[66] as previously described[67]. We identified 11 regions that could be spanned by a single BAC clone (**Supplementary Data 63**). High quality sequencing validated 9/11 inversion events with two regions (chr22:21,442,966-21,496,091 and chr17: 43,234,311 - 43,323,702) completed embedded within tandem duplications that could not be resolved at the level of clone based assembly. Of these 9 regions, 6 show evidence of an inverted orientation in all genomes analyzed indicative of misassembly in the GRCh38 reference. For the remaining 3 events, strand seq analysis in one individual supports the existence of the reference orientation suggesting it reflects the minor allele. We next selected 10-20 Kb of flanking sequence surrounding each of the 9 inversion events and identified that they are enriched for common repeat sequences (average repeat content 67%). Notably, 5 of these events are flanked by inverted LINE/L1 repeat sequences (**Supplementary Figure 43**).

Next, we identified 3 regions >1 Mb flanked by large highly identical segmental duplication blocks that showed evidence of inverted orientation >7 genomes. We selected two of these regions for a more detailed analysis by constructing alternate reference haplotypes using CHM1 BAC clones. At chromosome 16p12 we generated a ~1.8 Mb alternate reference haplotype corresponding to chr16:21288212-22746306 in the GRCh38 reference assembly. The inversion maps to a previously identified assembly error in the human reference genome[68] (**Supplementary Figure 43B**). Of the 9 human genomes analyzed here, a single individual is heterozygous for the event suggesting the reference may represent a very rare haplotype structure. At chromosome 2q13 we generated a ~843 Kb alternate reference haplotype spanning two large duplication blocks of ~358 Kb and >99% sequence identity (**Supplementary Figure 43C**). The duplication blocks map in inverted orientation and would likely predispose this region to inversion. Sequence analysis

shows that the CHM1 haplotype maps in direct orientation consistent with the GRCh38 reference assembly.

# 6. Biological context

## 6.1 Population genetics of SVs

### Population genetics of PacBio Integrated callset

**SVTyper genotyping:** The deletion calls from the HAN, PUR, and YRI children were separately genotyped in the Simons Human Genome Diversity Panel (HGDP) (n=238 samples), genotyping 6036, 6218, and 5865 SVs per sample. After removing sites with LD> 0.2 and MAF < 0.10, 3,082, 3,074, and 2,582 sites remained. Similar to the PCAWG analysis, the PCA demonstrated population based clustering, as expected.

We computed $F_{ST}$ across genotypes for each sample, with the populations separated as 35 African, 16 Amerindian, 41 East Asian, 21 Oceanic, 34 South Asian, 20 SIB, 72 West Eurasian. A total of 109, 109, and 112 sites were found to have FST > 0.20 from each sample, respectively, of which 33, 35, and 43 intersected genes. Of these, one event genotyped from HG00733 was found to be exonic (*TUBA3E*, chr2:130197357-130199831, $F_{ST}$=0.20), and one event genotyped in NA19240, PLIN4 (del chr19:4512828-4513027, $F_{ST}$=0.22) were found to overlap exons.

**SMRT-Genotyper summary**: With sequence-resolved structural variants from local assemblies, it is possible to genotype with more confidence. We used a version of the SMRT-SV genotyper adapted to handle larger contigs generated by this study. Since SMRT-SV requires an aligned contig with annotated breakpoints, we were only able to apply the SMRT-SV genotyper to Phased-SV variant calls.

We selected 24 PCR-free population samples from the 1000 Genomes Project (phase 3, **Supplementary Data 64**) and short-read sequences for the 3 child samples in this study. The number of genotyped variants were summarized in **Supplementary Data 65.**

### Removing tandem-repeat variants

Because tandem repeats represent a significant challenge for short-read alignments and inference drawn from them, we have omitted variants that affect tandem-repeat bases. Tandem-Repeat Finder (TRF) annotations were downloaded from UCSC for hg38. TRF annotations within 200 bp were merged into regions, and any variant with at least 10% of its reference bases in a region was excluded from analysis.

### Genotype Accuracy

We consider a variant genotypable if it has a call in at least 20% of the 1000 Genomes samples. If these variant calls are assumed to be correctly genotyped, they can be used to estimate genotyper performance using Illumina sequence data from the same sample. Genotyping accuracy is defined as the proportion of variants where the genotype matches variant call zygosity, and it is calculated omitting variants with a no-call genotype. See **Supplementary Data 66** as the genotyper summary of call proportions by sample and type for non-tandem-repeat variants, and Mendelian inheritance were calculated for all trios and summarized in **Supplementary Data 67**.

## 6.2 Functional analysis and annotations

### Functional enrichment analysis

The SV calls from the integrated PB-SV set were compared with annotations of genes, exons, CDS, intron, transcribed processed pseudogenes, transcribed unprocessed pseudogenes, and transcripts were taken from Gencode v25, along with transcription factor binding site peaks (Gencode + funseq), ultra sensitive, and ultra conserved elements from funseq (**Supplementary Figure 44**). While there was a depletion of

deletion SVs in all categories of genes, there was an increase in insertions within genes including exons and CDS.

## Structural variants engulfing genes

**(Contributed by: Nodzak, Wen, Shi)**

The variants in the pan-technology integrated set harbor regions that completely overlap with protein coding genes. In the IL-SV set, 573 SVs engulfed whole genes including 183 deletions, 182 inversions, and 208 duplications, while in the PB-SV set, 21 deletions were found to completely engulf genes (**Supplementary Data 68**). To assess the gene expression impact of the SVs that engulf genes, group *t*-tests were performed between the RPKM (Reads Per Kilobase Million) normalized expression values of real SV-engulfed genes and genes engulfed by analogous sets of randomly permuted regions using Strand-Specific RNA-seq data. To this end, we devised a method that found protein coding genes that were overlapped 100% by deletions, duplications and large inversions. This step was completed using BEDtools intersect with complete fractional overlap of sample-specific integrated Illumina SVs (filtered on a QUAL value of PASS and non-missing genotypes) on protein coding genes from gencode.v25.gtf, using the -f 1.0 command option (Williams 1989). The process was then repeated for deletions from the PB-SV deletion calls. Next, controlling for the size and chromosome of the SVs that engulf genes, 10,000 permutations were performed to create a set of random genomic regions with BEDtools shuffle. These permuted sets of regions were then used to identify sets of engulfed genes in the same manner described above. Specifically, strand-specific mRNA aligned to GRCh38 read quantification was performed for protein coding genes annotated by the gencode version 25 GTF using featureCounts, part of the Subread package[69]. Coverage normalization was then performed using the RPKM method, which resulted in a final set of expression values of 18,873 genes for the nine samples[70]. With each set of RPKM values for genes overlapped by a real variant call in a given sample, a group t-test was performed against the RPKM values for each of the lists of genes overlapped by the permuted regions. Finally, multi-test correction was applied using the fdrtool package in R [71]. The q-values were then -log2 transformed and the average was plotted for each sample to assess the significance of the effects of each type of variant on the expression of engulfed genes (**Supplementary Figure 45**).

Our results (**Supplementary Figure 45**) illustrated a high level of congruence of the expression effect brought about by these gene-engulfing SVs across the samples. Particularly, the expression of IL-SV deletion-engulfed genes for all nine samples showed significant differences from the permuted genes. Similarly, all three trio daughters showed significant differences in expression for those genes completely overlapped by large IL-SV inversions. 7/9 individuals were found to be impacted in a similar manner for the sets of whole-gene duplications. When the same analysis was conducted using the PB-SV deletions for the three trio daughters, we found 21 deletions were found to completely engulf protein coding genes (**Supplementary Data 68**) and one of the three individuals showed significant differences in the expression of the affected genes (**Supplementary Figure 45**).

## Indel functional annotations

**(Contributed by: Wen, Shi)**

We annotated the integrated Illumina indel callset for three trios using the variant effect predictor (VEP)[72]. Of 1,743,129 autosomal small indels from the Illumina indel integrated set, 1,944 indels were located in coding sequence region. For indels with different predicted consequences, we only counted one entry for each indel corresponding to its impact of the longest transcript (**Supplementary Data 69**), where the transcript annotation file used was Gencode.v25.transcripts.fa. 44.96% (874/1,944) of these indels were frameshift variants (FS) and 52.78% (1,026/1,944) were non-frameshift variants (NFS). In addition, 44 indels were annotated as other types of variants including coding sequence variant, protein coding variant,

splice acceptor variant, splice donor variant, stop gained variant and nonsense mediated decay (NMD) transcript variant.

## Allele specific expression analysis

**(Contributed by: Wen, Nodzak, Shi)**

Allele specific expression (ASE) analyzes differences in expression by leveraging heterozygous sites in diploid organisms. Former studies found up to 30% of loci showing allelic-specific effect on the transcript at individual level[73]. One study showed that approximately 20% of human genes can be affected by ASE in European populations[74]. Another study reported that nearly 18% of variants that located in protein coding regions showed ASE in HapMap populations[75].

We conducted ASE analysis of SNPs and SVs using the strand-specific RNA-seq data on the trios (**Supplementary Figure 46**). We started with SNP ASE analysis, based on the strand-specific RNA-seq data and the Whatshap strand-seq 10X phased SNPs using a pipeline which conducted mapping bias correction using WASP[76] and tested for ASE by applying binomial tests with multi-correction test on FDR 5%[77].

The pipeline of SNP ASE analysis includes the following steps. First, the strand-specific RNA-seq fastq files were mapped using the STAR (v2.4.2a) with default option to the GRCh38 human reference to create bam alignment files[78]. Second, we adapted WASP[76] to correct mapping biases as follows. Specifically, the STAR bam files were remapped to all SNPs and we discarded the reads not mapped to the same location with reference allele after flipped to the alternative allele. Third, duplicate reads were then removed using Picard (http://broadinstitute.github.io/picard) where the reads with the best quality and least mismatch were kept. Fourth, we used the following criteria to keep uniquely mapped reads for quality filtering. These reads will have an i) NM <= 6, ii) a base quality >= 10, iii) a mapping quality score > 20, and iv) total read count >=8 at each both allele seen heterozygous SNP (het-SNP) site. If the four criteria above were satisfied, then the number of reference allele count and alternative allele count were extracted with perl script *samase.pl* provided by Kukurba et. al[79], and a subsequent binomial test was performed with FDR-based (5%) multi-test correction conducted afterwards.

To evaluate the effect of overdispersion in the Strand-Specific RNA-seq data, we selected YRI mother NA19238 to calculate the overdispersion to compare the binomial test and beta-binomial test[80] with the same input for both allele seen heterozygous SNPs. The low overdispersion (0.0152) indicates that our RNAseq data is not overdispersed, and the binomial test and beta-binomial test for ASE analysis would give similar results (**Supplementary Figure 47**). Hence, we chose to use binomial test for our WASP based ASE analysis pipeline in this study (The whole ASE analysis pipeline can be found in https://github.com/shilab/HGSVC_ASE_Analysis).

Using this pipeline, we identified a total of 4,292 SNPs that exhibit ASE in the three trios(http://ftp.1000genomes.ebi.ac.uk/vol1/ftp/data_collections/hgsv_sv_discovery/working/201707_ASE_RES_Trios/). We overlapped these ASE SNPs with Ensembl genes using annotations by Gencode v25 protein coding genes. We found that 3,609 of these ASE SNPs affect 1,947 Ensembl genes in these three trios (YRI trio: 1,647 SNPs showing ASE on 1,043 Ensembl genes; PUR trio: 1,293 SNPs showing ASE on 857 Ensembl genes; CHS trio: 1,138 SNPs showing ASE on 703 Ensembl genes). We overlapped all of the 4,292 ASE SNPs we identified with published ASE results, and found that 72.8% (3,124/4,292) of our ASE SNPs and 97.5% (1,899/1,947) of our ASE genes were previously reported as tagging ASE loci in LCLs[80–82]. Among these, 246 ASE SNPs identified were also previously reported as eQTLs[41,81,83–91]. We also overlapped these 4,292 ASE SNPs identified in this study with GWAS catalog[92], 35 of them were GWAS signals associated with 32 human traits including diabetes, obesity, and Alzheimer's disease. We performed a permutation-based test to assess if ASE SNPs are enriched for known eQTLs, and in

consistent with prior reports[89,93,94] we found that ASE SNPs are significantly enriched for eQTLs in LCLs (**Supplementary Figure 48**).

To functionally characterize the identified ASE genes, we conducted Gene Ontology (GO) enrichment analysis (GO annotation released 2017-11-28)[95,96]. The PANTHER overrepresentation test (Fisher's exact test with FDR-based multiple test correction at 5% FDR cutoff) was used for enrichment testing for three GO categories - molecular function, biological process and cellular component. REVIGO was used to reduce the redundant significant GO terms and group GO terms for visualization[97]. 20, 31, 18 unique GO terms were found for the molecular function, biological process and cellular component categories, respectively. These data are summarized in the Supplement including through the Supplemental **Supplementary Figures 49, 50, and 51.**

We next assessed the overlap between our ASE genes and genomic imprinting. Of 1,947 ASE Ensembl genes identified in the three trios, 30 were overlapped with experimentally validated and predicted imprinting genes in prior studies[98,99,100] (**Supplementary Data 70**). Two of these imprinted genes, *SNURF*/*SNRPN* and *GNAS*, were previously reported as imprinted ASE genes[93]. *SNURF*/*SNRPN* is a paternally expressed gene, and our ASE result showed a consistent inheritance pattern with the imprinted pattern (mRNA read counts of two alleles at 283 vs 1 for YRI father and 213 vs 1 for YRI child; 1 vs 287 for CHS father and 2 vs 230 for CHS child). Genomic imprinting is an epigenetic phenomenon that causes genes to be expressed in a parent-of-origin-specific manner, leading to ASE. But imprinting is just one out of many molecular mechanisms leading to ASE, and thus we certainly would not expect that a much larger portion of the ASE genes reported are undergoing imprinting. Previous studies have been consistent in reporting that relatively small fractions of ASE genes are due to imprinting[73,101], which is consistent with our data.

Once we characterized ASE SNPs, we sought to investigate if SVs brought about ASE effect on those genes whose expressions were shown to be allele specific from the SNP-ASE analysis above. In order to do so, we developed an SV-ASE analysis pipeline (**Supplementary Figure 46**) with the following three steps for PB-SVs and IL-SVs respectively. First, we established a set of candidate SVs-gene pairs by taking the intersection of heterozygous SVs (het-SVs) with SNP-ASE genes. Second, phased RNA-Seq reads were filtered following the same criteria established by our SNP-ASE analysis above and read counts of the genes were calculated for each sample's two haplotypes using BEDtools multicov. Third, the significance of SV-genes pairs was then obtained by applying a binomial test to the read counts of the two haplotypes with multi-test correction using FDR 5%.

Our results (**Supplementary Data 71**) showed that the majority of het-SVs tested significantly affected the target gene expression in allele specific manner. Specifically, in the PB-SV set, a total of 144 SVs (70 insertions and 73 deletions) showed ASE effect on 60 genes, out of the 199 het-SVs intersected with 78 SNP-ASE genes for NA19240; a total of 196 SVs (88 insertions and 108 deletions) showed ASE effect on 77 genes, out of the 220 het-SVs intersected with 85 SNP-ASE genes for HG00514; and a total of 219 SVs (141 insertions and 78 deletions) showed ASE effect on 89 genes, out of the 274 het-SVs intersected with 106 SNP-ASE genes for HG00733. In the IL-SV set, 58 SVs (7 insertions, 48 deletions and 3 inversions) demonstrated ASE effect on 59 genes, out of the 83 het-SVs intersected with 62 SNP-ASE genes for HG00514; 60 SVs (10 insertions, 45 deletions and 5 inversions) demonstrated ASE effect on 60 genes, out of the 108 het-SVs intersected with 78 SNP-ASE genes for HG00733; and 57 SVs (6 insertions, 48 deletions and 3 inversions) demonstrated ASE effect on 44 genes, out of the 79 het-SVs intersected with 55 SNP-ASE genes for NA19240.

Our SV-ASE results prompted us to address whether or not the observed allelic imbalance at SV-ASE genes was attributable to a local haplotype along the gene region. For this, we calculated the LD ($R^2$

values) between the SVs and SNPs with ASE effect on the same gene. We illustrated this analysis to assess the allelic effect resulting from a heterozygous deletion belonging to HG00514 within a transcription factor binding site on exon 5 of the *ZNF717* gene (**Supplementary Figure 52**). We further ruled against a haploblock effect driving the allelic imbalance between the haplotype from low $R^2$ values for the sample's variants and those from the 1000GP phase3 CHS population within a window ± 100 Kb of the gene, and showed that there were few variants with high R^2 within the exon as well.

### Variants overlapping GWAS sites

We searched for deletion SVs that overlapped GWAS sites, since common structural variation that has not been detected could affect interpretation of allele frequency. The results of SV overlapping with 11,737 GWAS sites is given in **Supplementary Data 75**. There were 16 deletions from HG00514, 9 HG00733, and 16 from NA19240 that were overlapping GWAS sites, with an average length of 286 Kb and median length of 16,302 bp.

## 6.3 MEI discovery and analysis with PacBio data

**(Contributed by: Gardner, E. Chuang, Nelson, and Devine, S.E.)**

Short-read Illumina-based approaches are not able to capture the interior sequences of potentially active 6 Kb FL-L1 retrotransposons. Thus, we sought to utilize our LA assembly data from the three children in our study (HG00514, HG00733, and NA19240) to assess reference (REF) FL-L1 sites (those found in the Hg38 reference genome assembly) and nonREF FL-L1 sites (those not found in the reference genome but found in one or more of the children's genomes). We developed two approaches, one each for REF and nonREF sites, to independently assess these two genomic compartments. Using these data, we then developed profiles of potentially active FL-L1 sites in the genome of each child. These profiles include annotation of the number of intact open reading frames (ORFs—either zero, one, or two), L1 subfamily analysis[102], and published information on whether the element is highly active (i.e., "hot"), active, or inactive[103,104] (**Supplementary Data 13**).

### Reference L1 assembly

For each proband, all FL-L1 (6 Kb) L1Hs and L1PA2 intervals in the human Hg38 assembly (Smit, AF) were reassembled using haplotype partitioned reads (Section 3.2 MsPAC). In short, given an L1 interval, (*i, j*), reads overlapping the coordinate interval (*i-10,000, j+10,000*) were passed as input to MsPac to yield a final assembly sequence containing the L1 interval. We determined with Sanger sequencing that Pacific Bioscience RSII (PacBio) sequencing consistently generated a reproducible error in discriminating the number of bases in four homopolymer tracts at FL-L1 positions 1117, 1699, 3135, and 3564. Thus, we systematically corrected deletions that were detected at these four positions. With this approach, 1042/1103 (94.5%) of the FL-L1 sequences that were examined in the three children were identical to their Hg38 counterparts. These results are consistent with previous reports indicating that FL-L1 sequences can vary across populations [105,106]. For each haplotype resolved sequence, assemblies were then queried for the presence/absence of an L1 to generate a genotype (0/0, 0/1, or 1/1) at that given locus.

L1 annotations were generated with a custom Python script that aligns each L1 of interest to a consensus L1. It then searches for the ORF1 and ORF2 regions, translates them, and calculates the respective protein sizes. Subfamilies are determined using a conservative interpretation of the classification scheme that was developed by Boissinot et al, where a complete match of each canonical base was required for an element to be assigned to a given subfamily. Subfamilies that had canonical Ta and PreTa bases, but did not have complete matches to the subfamily were flagged as Ambiguous-Ta (Ambig-Ta) or Ambiguous-PreTa (Ambig-PreTa), respectively. If there were no canonical base matches, then it was considered L1 Ambiguous (L1Ambig) .

## Non-reference L1 polishing

To determine the polymorphic nonREF L1 component of each proband's genome (i.e. for HG00514, HG00733, and NA19240) we utilized the repeat annotation provided within the unified PacBio call set VCF (Section 3) to extract all L1 insertion variants labelled as human specific (L1Hs; Section 1). Using Smith-Waterman alignment, we then aligned each extracted L1 to a known, active FL-L1[107] to determine subfamily, number of intact open reading frames (ORFs), and overall sequence composition. On initial assessment, we observed that a number of our L1 assemblies had more than the expected number of indels compared to our reference L1 element (**Supplementary Figure 53**). As PacBio sequencing can introduce an overabundance of indels in assembled sequences and these observed indels disrupted coding sequence internal to our assemblies, we sought to determine the validity of all indels in our PacBio assembled nonREF L1Hs elements. To accomplish this goal, we developed a novel approach utilizing 10X Genomics GemCode sequencing to polish all PacBio assembled L1Hs from each proband's VCF file.

Our 10X polishing approach has four steps. First, we sort the 10X bam file based on the "BX" tag which contains the GEM read cloud barcode generated during 10X sequencing (section 1.2 10X genomics). During this step, we also calculate the total number of reads attributable to each tag and filter tags which have less than 15 reads or more than five times the median number of reads per tag in a given sample (**Supplementary Figure 53A**). Next, we generate a file index containing the precise location of each read cloud within our sorted bam file. After indexing, we iterate over all nonREF L1 loci, recruiting read clouds which support the presence of an L1 insertion at that given locus. Evidence consists of either discordant pairs where one mate aligns to the reference and the other aligns to an L1, or split reads where part of one mate aligns to both the reference and an L1. All reads from each supporting read cloud are then aligned to the PacBio L1 assembly at a given locus. Finally, supporting reads are processed using a kmer approach [108] and queried using overlapping 21-mers for consistency to each PacBio L1 assembly at every base within that assembly. Bases in the assembly not supported by the kmer library are then iterated over to determine the highest likelihood base, deletion, or insertion at that position. Bases where we could not determine the correct residue/variant due to insufficient evidence were reported as "N" in the final output. Polished sequences are then reported in fasta format and annotated as outlined above for REF elements. Using this approach, we were able to generate polished (i.e. no "N" bases) sequences for between 76 and 93% of all PacBio assembled nonREF L1Hs elements per genome (**Supplementary Figure 53**).

Considering that the average 10X read cloud size is in the tens of Kb in magnitude, we also sought to ensure that we were not recruiting adjacent L1Hs reads when building kmer libraries which may lead to incorrect polishing. To test for this scenario, we examined the *n* bases (where *n* is the average read cloud size in a given individual) surrounding the genomic coordinate of each polished L1Hs element for both REF and nonREF L1Hs or L1PA2 sequences as annotated by RepeatMasker. We found that the majority of polished L1Hs elements did not have any other young, and thus potentially polymorphic, L1 elements within their read cloud that could potentially lead to errors in our polishing approach (**Supplementary Figure 53C**).

## Description of Supplementary Figures


Supplementary Figure 1. Examples of critical regions of microdeletion syndrome loci flanked by inversions.

Supplementary Figure 2. Intact FL-L1 source element profiles for the three children.

Supplementary Figure 3. The number of phased SNVs in 60 kbp windows across the genome.

Supplementary Figure 4. PacBio Coverage Permutation Test.

Supplementary Figure 5. Bionano Concordance.

Supplementary Figure 6. MsPAC and PacBio SV site comparisons.

Supplementary Figure 7. Closest distance between Phased-SV and MsPAC calls.

Supplementary Figure 8. SV callset size by merging parameter.

Supplementary Figure 9. Tandem repeat cluster sizes.

Supplementary Figure 10. Pipeline to integrate SVs from multiple Illumina callsets.

Supplementary Figure 11. Assessment of breakpoint accuracy of each Illumina algorithm.

Supplementary Figure 12. The deletion can be confirmed by raw PacBio read-support in an alignment-free approach using dot-plots.

Supplementary Figure 13. Alignment pattern of a 14Kb deletion that was uniquely discovered by Illumina algorithms.

Supplementary Figure 14. Illumina-genotyping, heterozygosity by depth of coverage.

Supplementary Figure 15. SGDP Sample Distribution Geographic distribution of the Simon's Genome Diversity Project samples

Supplementary Figure 16. Locating and genotyping inversions in Strand-seq data.

Supplementary Figure 17. Putative Inversions discovered in Strand-seq composite files.

Supplementary Figure 18. Haplotype structure of homozygous inversions discovered by Strand-seq.

Supplementary Figure 19. Summary of inversion callsets included in the integration analysis.

Supplementary Figure 20. Intersection test results for HG00733.

Supplementary Figure 21. Summary of reciprocal overlap of inversions by method.

Supplementary Figure 22. Inversion lists used to generate the final unified inversion callset.

Supplementary Figure 23. Parent-child trios characterized for structural variation.

Supplementary Figure 24. Insert size distribution of an example HGSVC liWGS library.

Supplementary Figure 25. SMRT sequence coverage.

Supplementary Figure 26. Data portal for IGSR samples.

Supplementary Figure 27. Map of meiotic recombination events for each family trio.

Supplementary Figure 28. Level of overlap between Strand-seq and PacBio derived meiotic breakpoints.

Supplementary Figure 29. Enrichment analysis of Alu and THE1-A,B elements around mapped meiotic breakpoints.

Supplementary Figure 30. Indel discovery summary.

Supplementary Figure 31. The number of insertions and deletions detected at each size from 0 to 49 bp were compared across all samples.

Supplementary Figure 32. BLASR and NGM-LR alignment comparisons.

Supplementary Figure 33. Dotplot comparison of alignments in tandem repeat regions.

Supplementary Figure 34. Excess SNV count by alignment method.

Supplementary Figure 35. Detailed view of excess SNV count by alignment method.

Supplementary Figure 36. Genomic distribution of SVs.

Supplementary Figure 37. Oxford Nanopore sequencing read length and coverage for HG00733.Supplementary Figure 38. Read support for SV calls for HG00733 from (red) ONT and (black) PB reads.

Supplementary Figure 39. Example of ONT SV detection with different base calling parameters.

Supplementary Figure 40. Overview of SVs discovered with all algorithms and a subset of algorithms on full coverage (75X) and down sampled (30X) datasets.

Supplementary Figure 41. The counts of SVs affecting genes by method and region of gene.

Supplementary Figure 42. Regions of SV that were called in the IL-SV callset and not PB-SV.

Supplementary Figure 43. Misassemblies in the GRCh38 reference validated by large insert clone based sequence and assembly.

Supplementary Figure 44. Enrichment for SVs intersecting functional elements in the IL-SV and PB-SV callsets.

Supplementary Figure 45. Significance of gene expression effect by PB-SVs and IL-SVs that engulf protein coding genes.

Supplementary Figure 46. A pipeline of the allele specific expression analysis for SNPs and SNVs.

Supplementary Figure 47. The distribution of reference allelic ratio using both allele seen het-SNPs.

Supplementary Figure 48. Enrichment analysis for eQTLs.

Supplementary Figure 49. Molecular functions enriched for ASE genes.

Supplementary Figure 50. Biological processes enriched for ASE genes.

Supplementary Figure 51. Biological processes enriched for ASE genes.

Supplementary Figure 52. Analysis of SNP and PB-SV ASE for indication against a haploblock effect.

Supplementary Figure 53. Correction of L1 insertions.

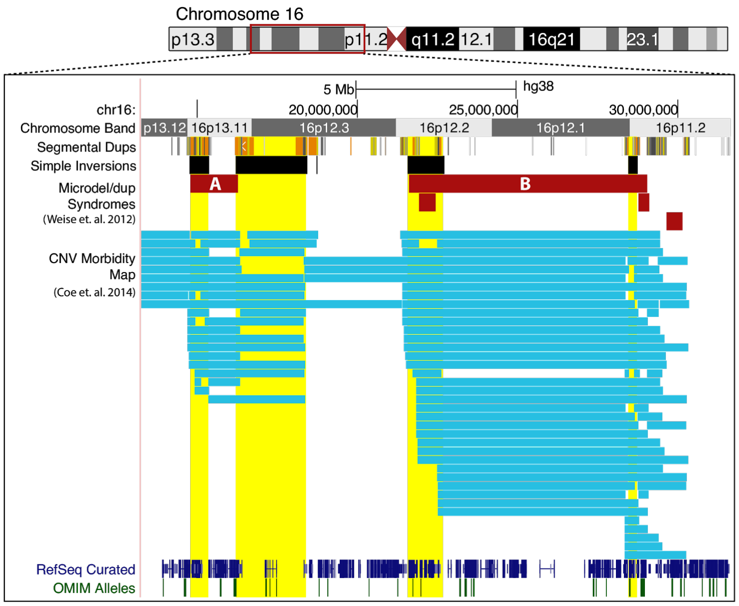


| A: | microdeletion 16p13.1 | |
|---|---|---|
| inversion | proximal | distal |
| coordinates | chr16:14.77-15.38 | chr16:16.21-18.44 |
| width | 608,587 | 2,225,858 |
| allele frac. | 0.67 | 0.11 |

| B: | microdeletion 16p11.2-p12.2 | |
|---|---|---|
| inversion | proximal | distal |
| coordinates | chr16:21.57-22.70 | chr16:28.46-28.74 |
| width | 1,134,231 | 282,778 |
| allele frac. | 0.94 | 0.22 |

Zoom in on 16p13.1 ('A') region

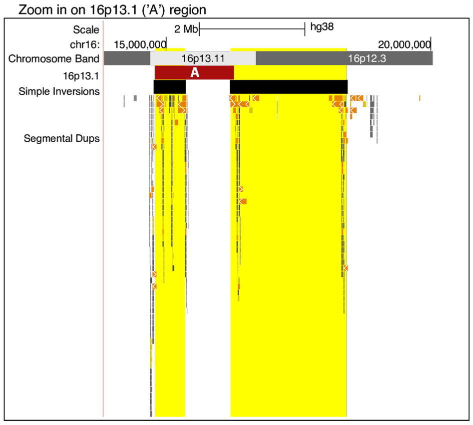


## Supplementary Figure 1.

**Examples of critical regions of microdeletion syndrome loci flanked by inversions.** Genome browser view of two microdeletions located on chr16p (red bars, labeled 'A' and 'B') flanked by inversions located in the present study (black bars with yellow highlight). Expanded view of the segmental duplications rearranged by the inversions flanking region A are shown in the right panel. The coordinates, length, and genotypes for each inversion are listed in the table. Blue bars are microdeletions found in donors from the CNV morbidity map.

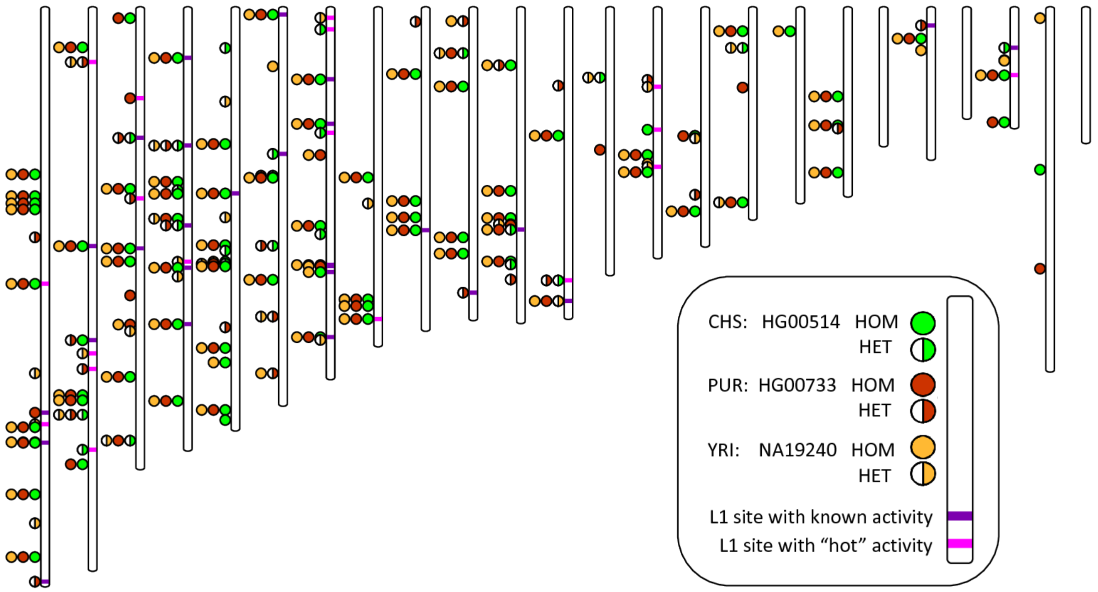


## Supplementary Figure 2.

**Intact FL-L1 source element profiles for the three children.** Chromosome 1 through 22, X, and Y are displayed from left to right. FL-L1s with two intact open reading frames are represented by a circle in the color corresponding to the individual. The circle can either be filled or half-filled depending on the genotype in the respective individual. L1 sites with activity documented in the literature are depicted by light purple (highly active or "hot") or dark purple (low to moderate activity) horizontal lines at the site.

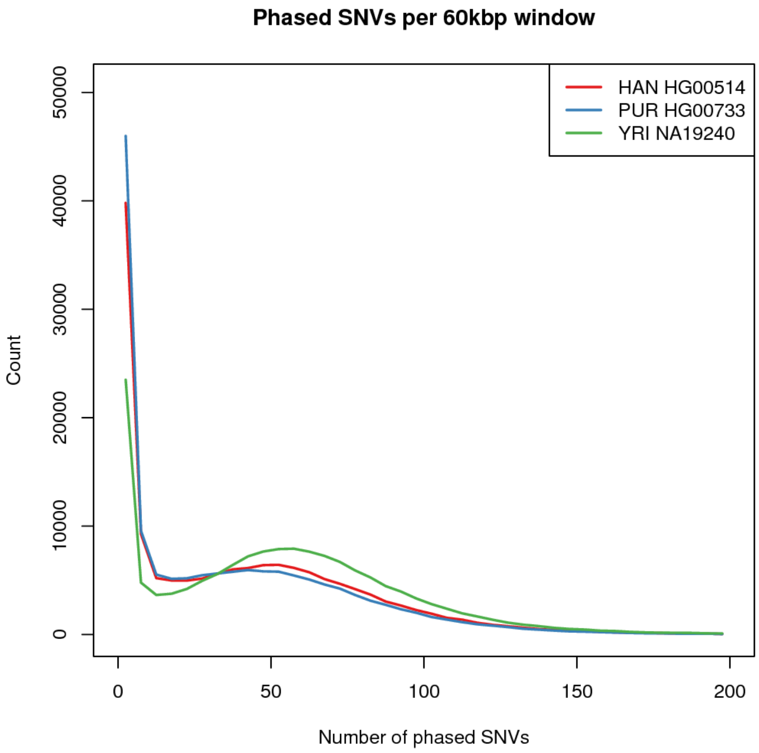


## Supplementary Figure 3.

**The number of phased SNVs in 60 kbp windows across the genome.**

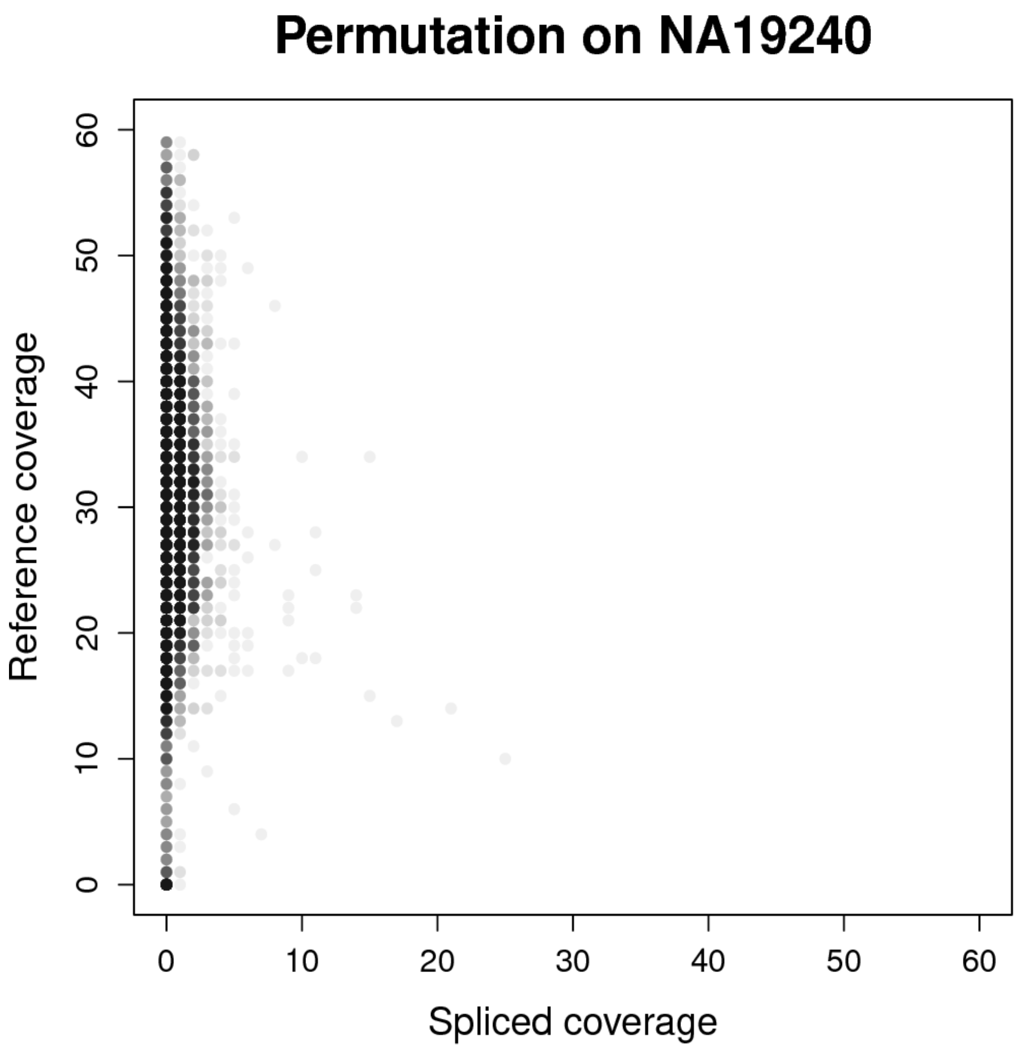


r

Supplementary Figure 4.

**PacBio Coverage Permutation Test.** The coverage of pacbio reads that map to a breakpoint when the breakpoints are randomly shuffled.

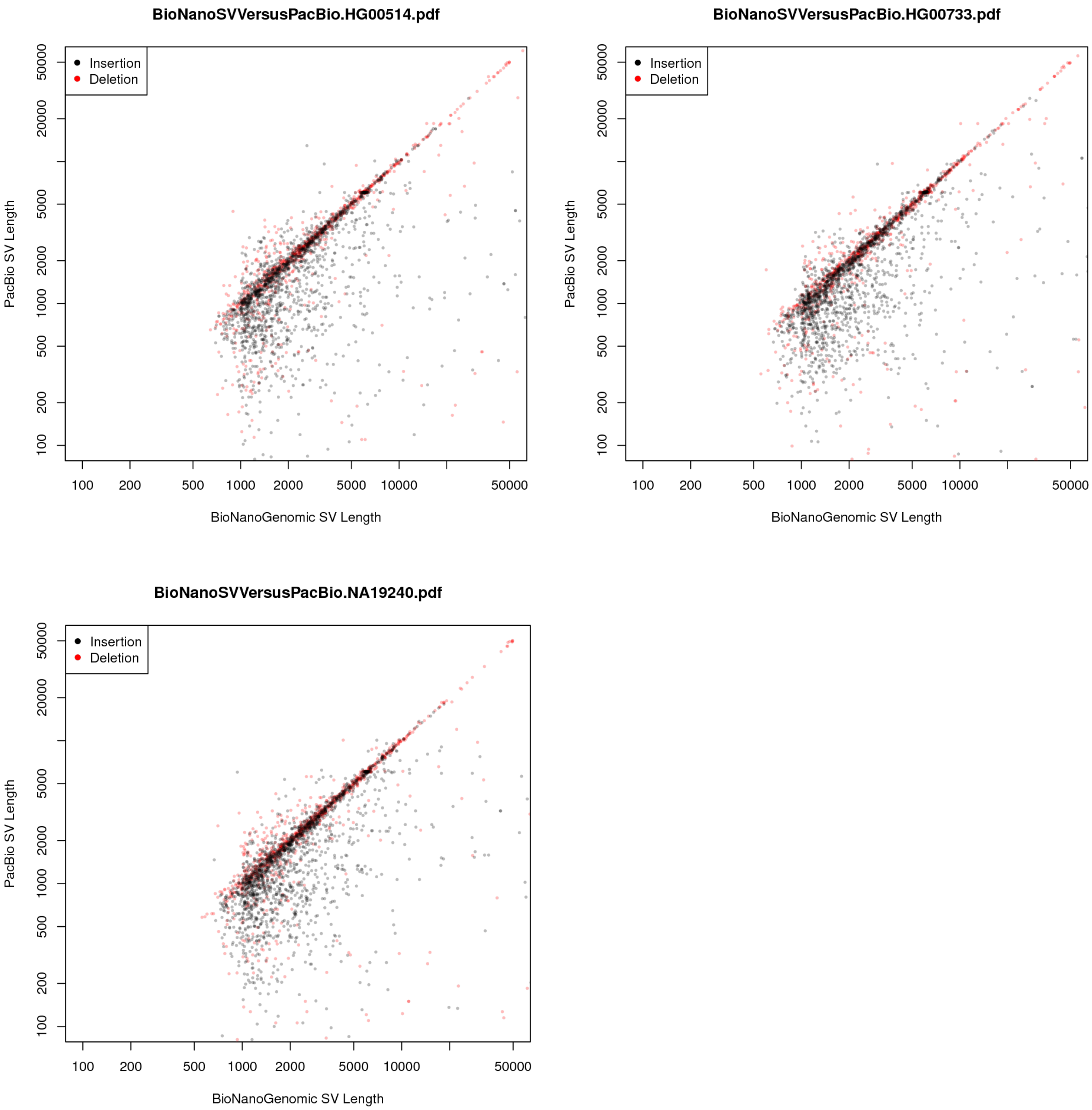


## Supplementary Figure 5.

**Bionano Concordance.** The concordance defined as the closest matching SV lengths across the variant boundaries of each Bionano variant call is shown for insertions (black) and deletions (red), for HG00514, HG0073, and NA19240.

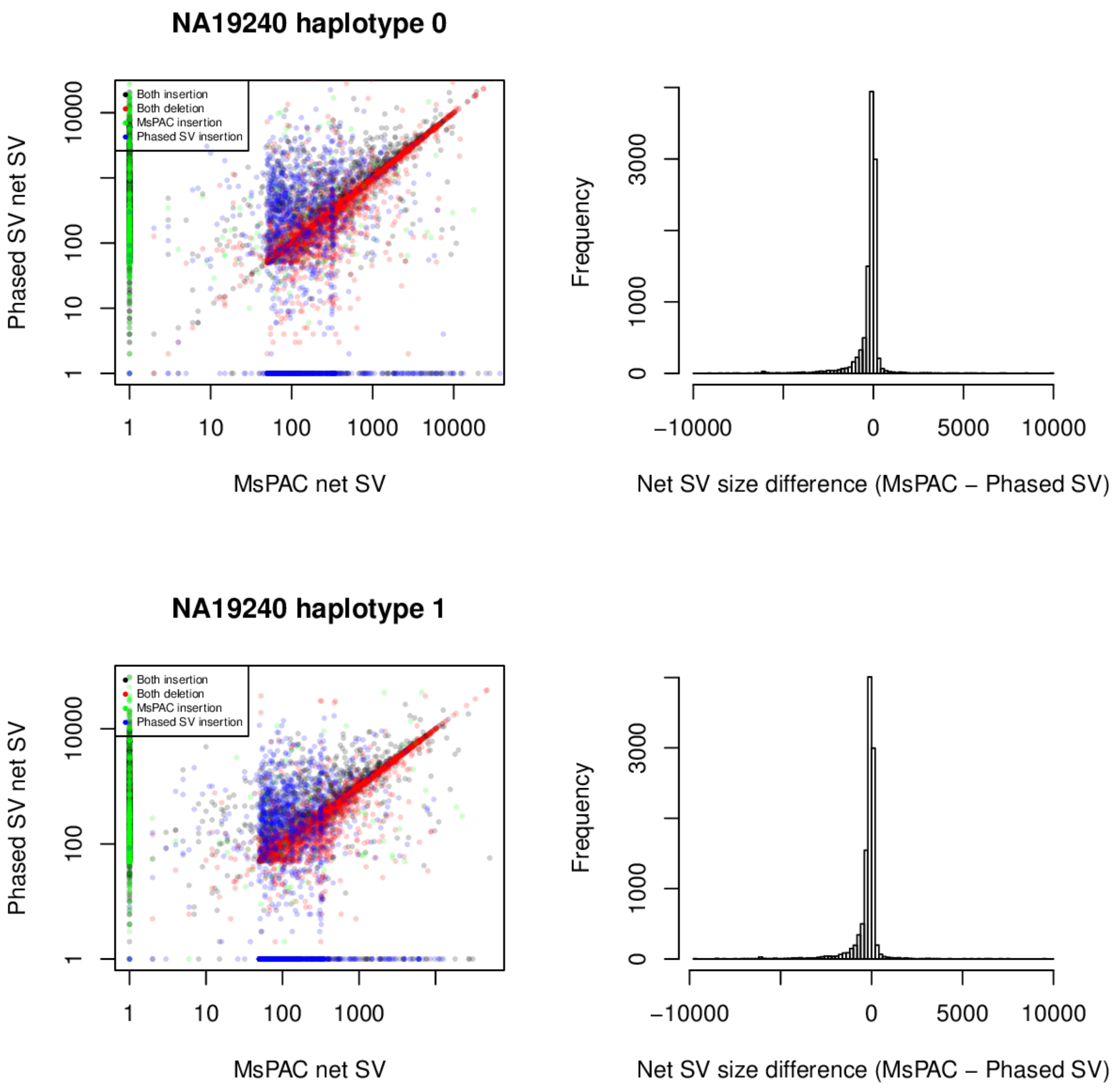


## Supplementary Figure 6.

**MsPAC and PacBio SV site comparisons.** (left) Net SV calls across 100 kbp bins in the euchromatic genome for both haplotypes of NA19240. (black) bins where both methods have a net insertion (red) bins where both methods have a net deletion (green) MS-PAC has a net insertion, and Phased-SV is less than or equal to zero (blue) Phased-SV has a net insertion, and MS-PAC is less than or equal to zero. (right) A histogram of the difference between net SV. A value of zero indicates the net SV insertion and deletion is equal between the two methods.

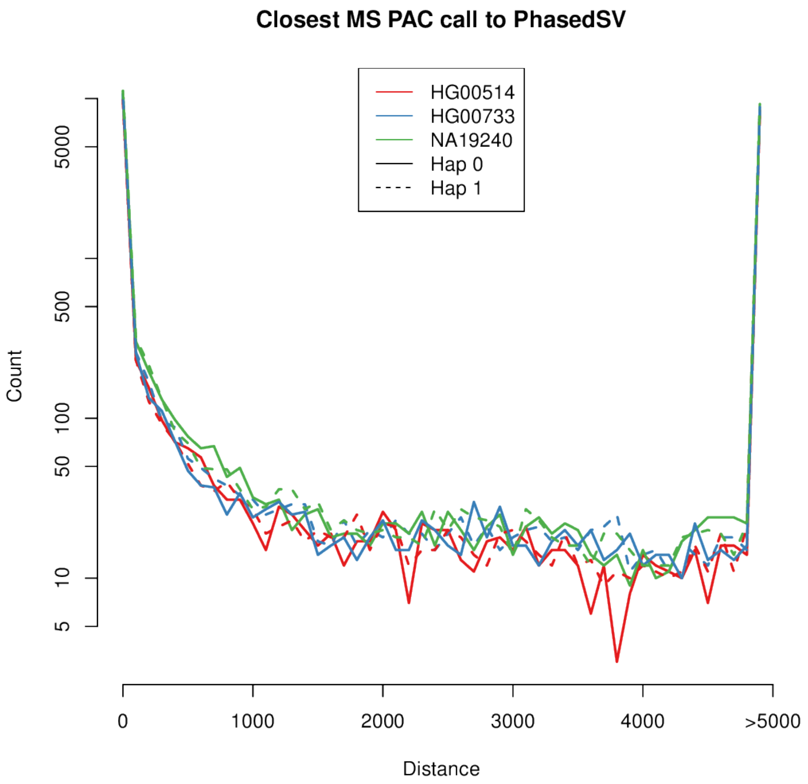


## Supplementary Figure 7.

**Closest distance between Phased-SV and MsPAC calls.** The closest distance to a Phased-SV call for all MS-PAC calls. All values > 5kbp are grouped at 5kbp.

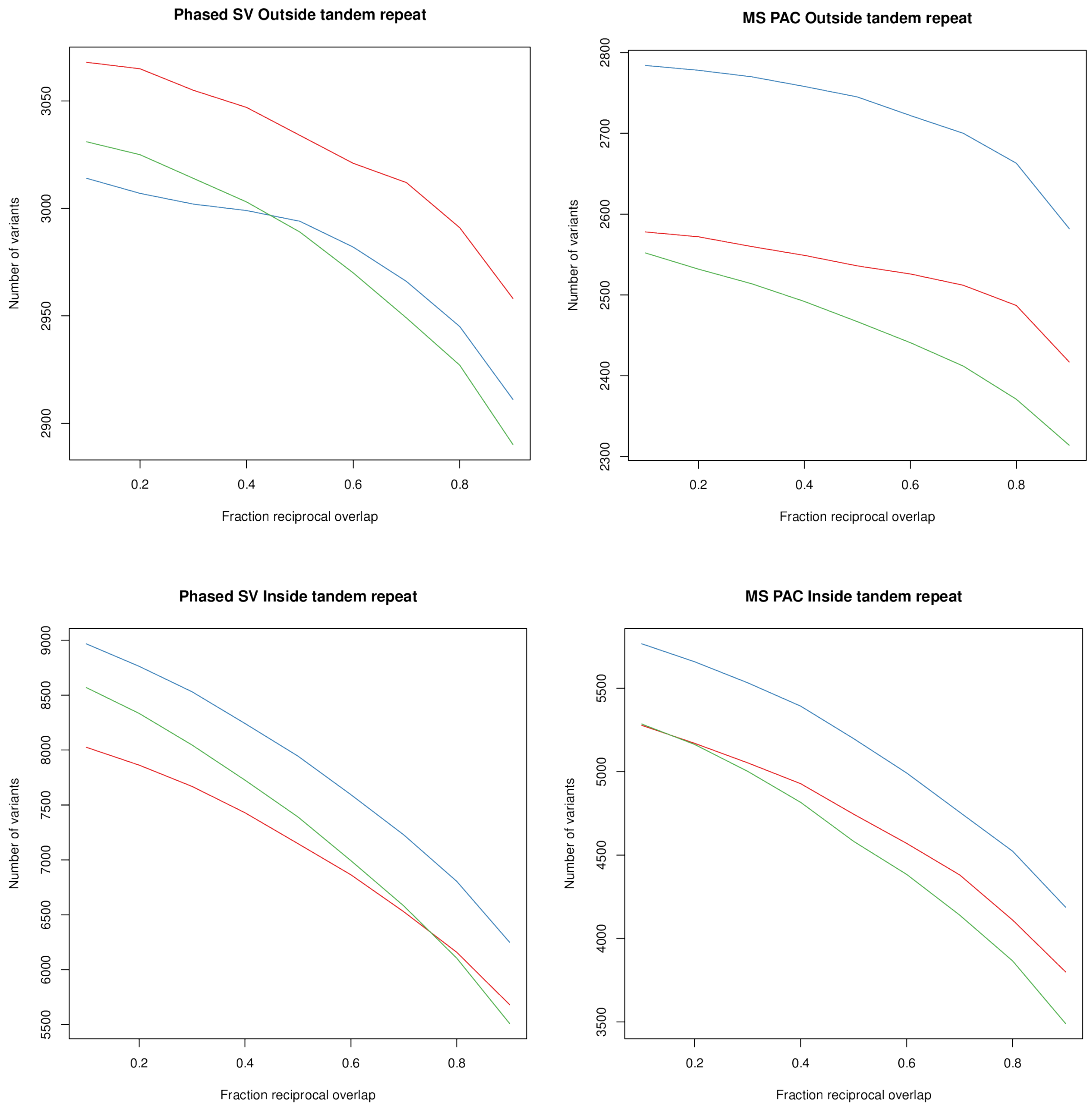


## Supplementary Figure 8.

**SV callset size by merging parameter.** Number of SVs after merging SVs between haplotypes for calls outside of tandem repeats (top), and inside tandem repeats (bottom), for Phased-SV (left) and MS-PAC (right) for HG00514 (blue), HG00733 (green), and NA19240 (red).

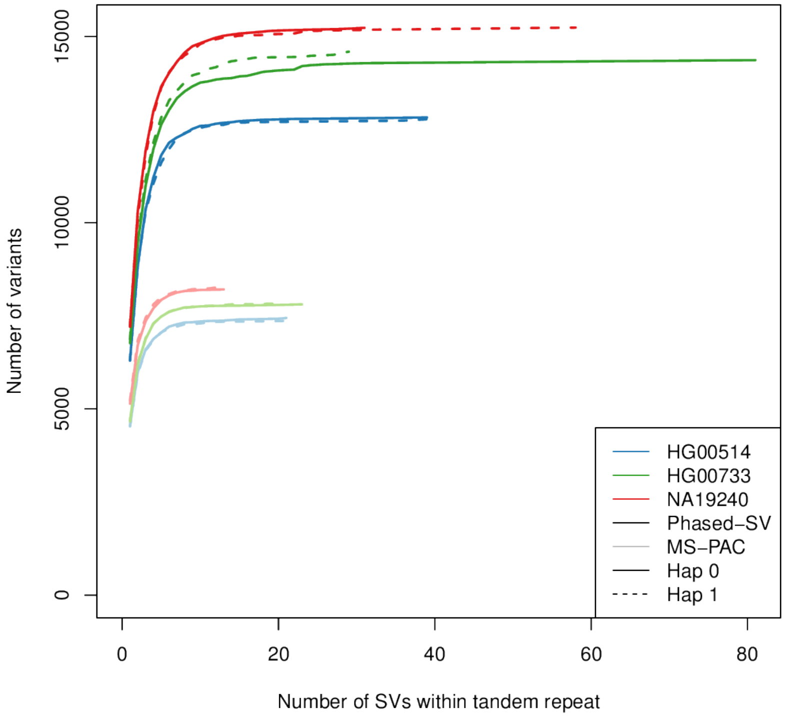


## Supplementary Figure 9.

**Tandem repeat cluster sizes.** The cumulative distribution of total number of SVs relative to the number of SVs in within tandem repeat loci.

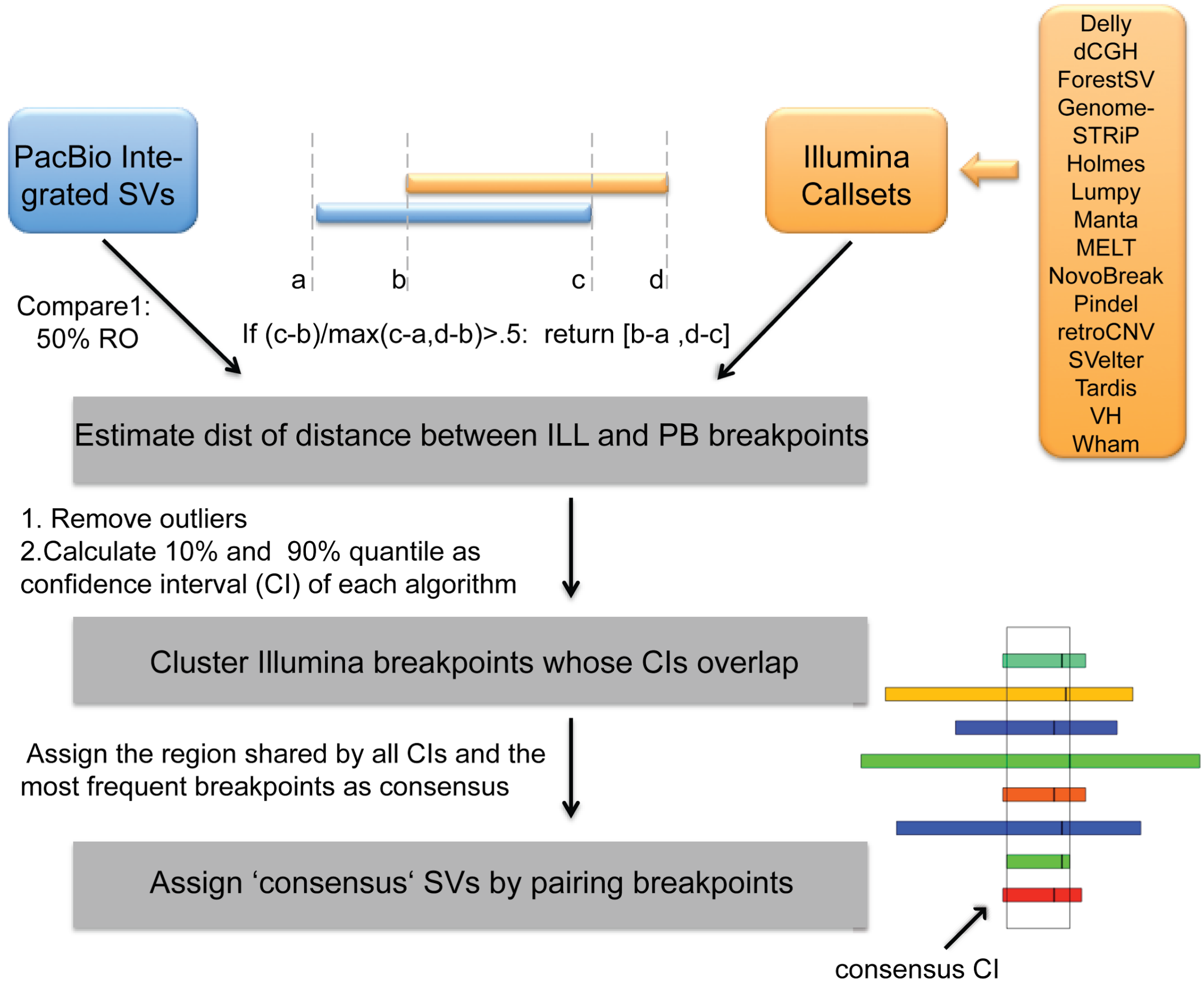


## Supplementary Figure 10.

**Pipeline to integrate SVs from multiple Illumina callsets.**

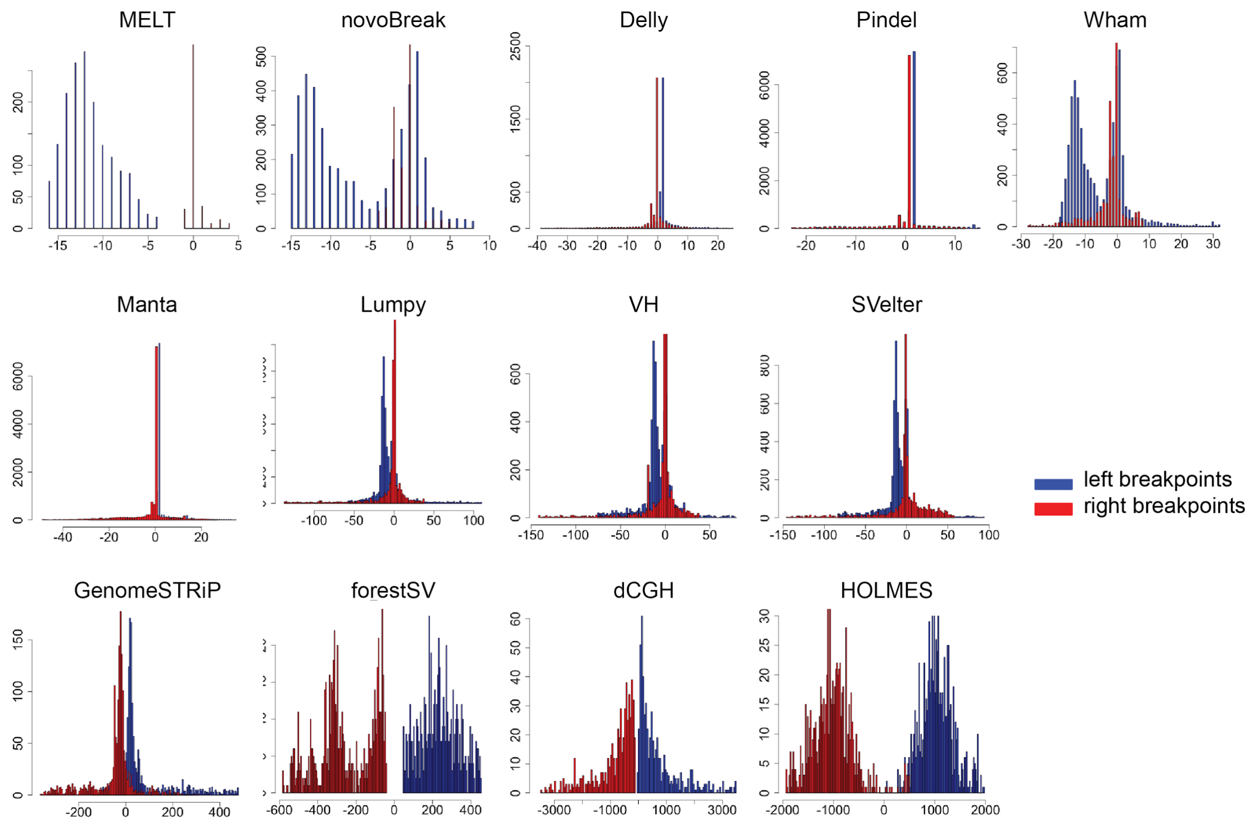


## Supplementary Figure 11.

**Assessment of breakpoint accuracy of each Illumina algorithm.**

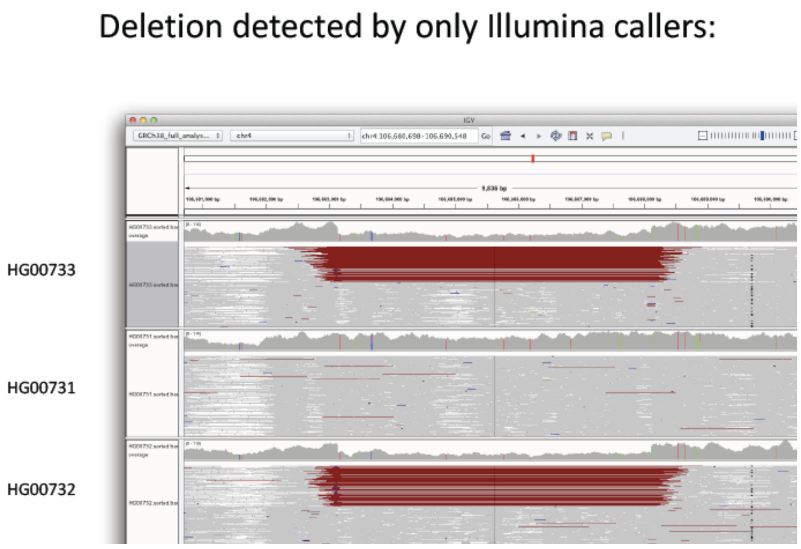


## Supplementary Figure 12.

**The deletion can be confirmed by raw PacBio read-support in an alignment-free approach using dot-plots.** A 6.5kbp PacBio read (m140817_221907_42175_c100689561270000001823145102281516_s1_p0/95354/0_6656) supporting the deletion is shown in the dotplot below.

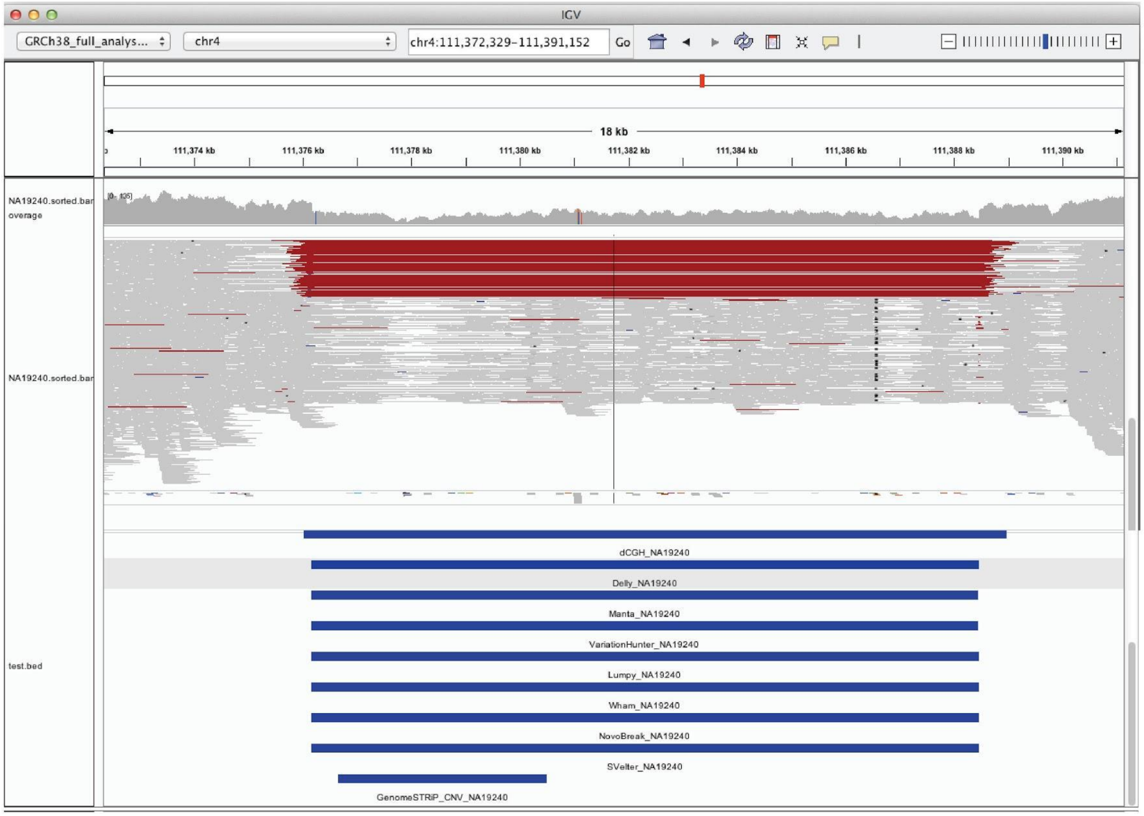


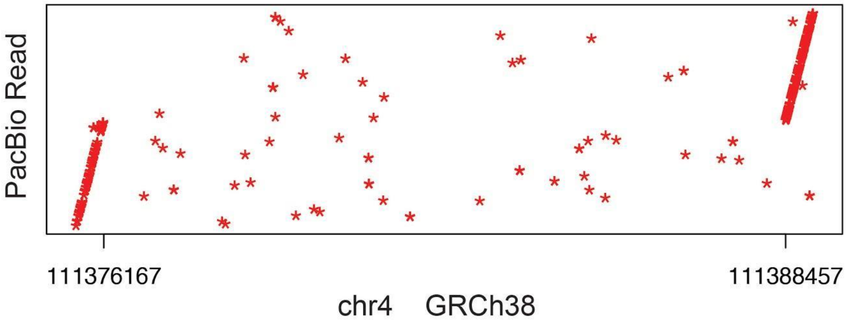


## Supplementary Figure 13.

**Alignment pattern of a 14Kb deletion that was uniquely discovered by Illumina algorithms.**

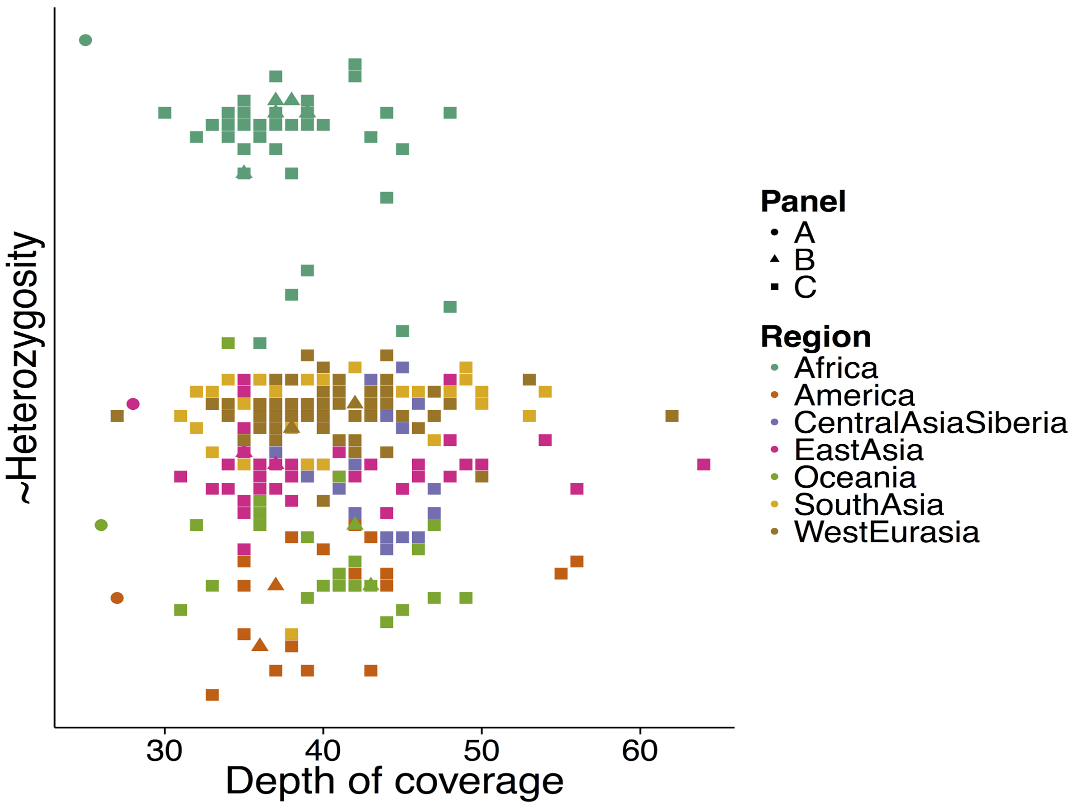


.

Supplementary Figure 14.

**Illumina-genotyping, heterozygosity by depth of coverage.** The depth and genetic diversity in each of the seven SGDP super populations.

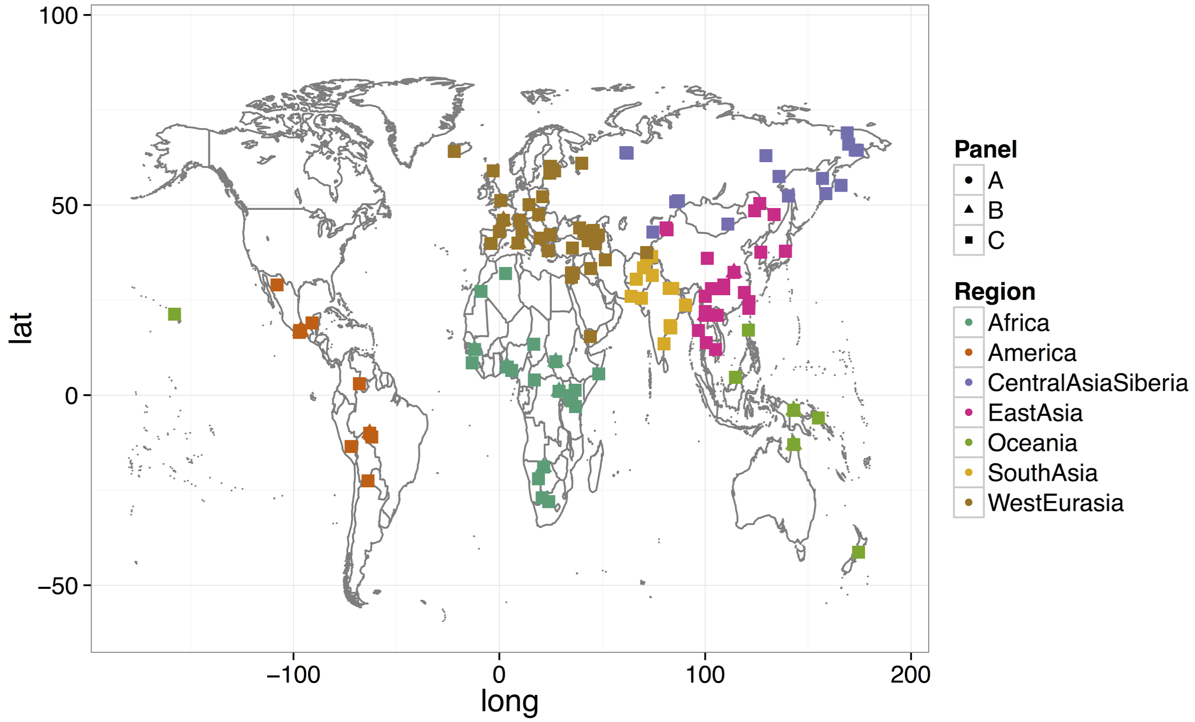


## Supplementary Figure 15.

**SGDP Sample Distribution Geographic distribution of the Simon's Genome Diversity Project samples.** Individuals without longitude/latitude data are excluded from this plot, e.g. Native north American.

A

| Trio | Line | Libraries | (%) Bases | (x) Depth |
|---|---|---|---|---|
| CHS father | HG00512 | 111 | 76.99 | 5.63 |
| CHS mother | HG00513 | 74 | 77.5 | 3.55 |
| CHS child | HG00514 | 76 | 80.29 | 4.19 |
| | | 261 | 78.26 | 4.46 |
| PUR father | HG00731 | 122 | 80.62 | 7.31 |
| PUR mother | HG00732 | 94 | 79.13 | 5.27 |
| PUR child | HG00733 | 115 | 80.3 | 5.95 |
| | | 331 | 80.02 | 6.18 |
| YRI father | HG00239 | 183 | 63.82 | 7.73 |
| YRI mother | HG00238 | 172 | 61.08 | 6.08 |
| YRI child | HG00240 | 117 | 80.48 | 7.11 |
| | | 472 | 68.46 | 6.97 |

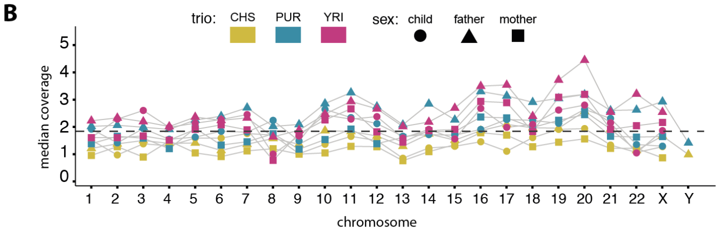


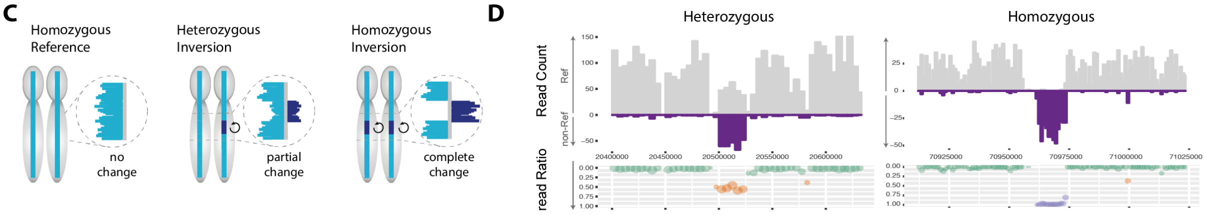


## Supplementary Figure 16.

**Locating and genotyping inversions in Strand-seq data.** A) Summary of the Strand-seq libraries used to generate the merged composite files for each chromosome B) the final median coverage of the composite per chromosome shown for each sample. C) strategy for locating and genotyping inversion in composite files. D) examples of a heterozygous and homozygous inversion identified by the proportion of reads mapping to the reference assembly in each orientation, which is used to calculate the read ratio.

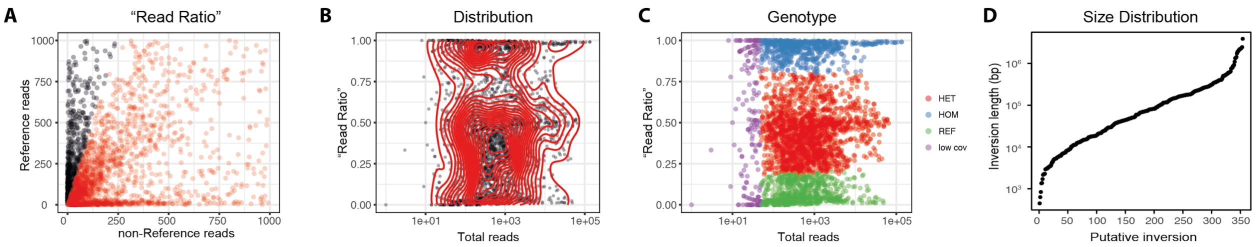


## Supplementary Figure 17.

**Putative Inversions discovered in Strand-seq composite files.** A) Dotplot of local read ratios calculated for the Strand-seq composite files. Each point represents a putative inversion, and its position reflects the proportion of reads in reference and non-reference orientation at that locus. Loci with a read ratio > 15% are shown in red. The B) distribution of read ratios, and C) genotype, as determined by Fisher Exact Test, were used to further classify these events. D) Size length distribution of predicted inversions.

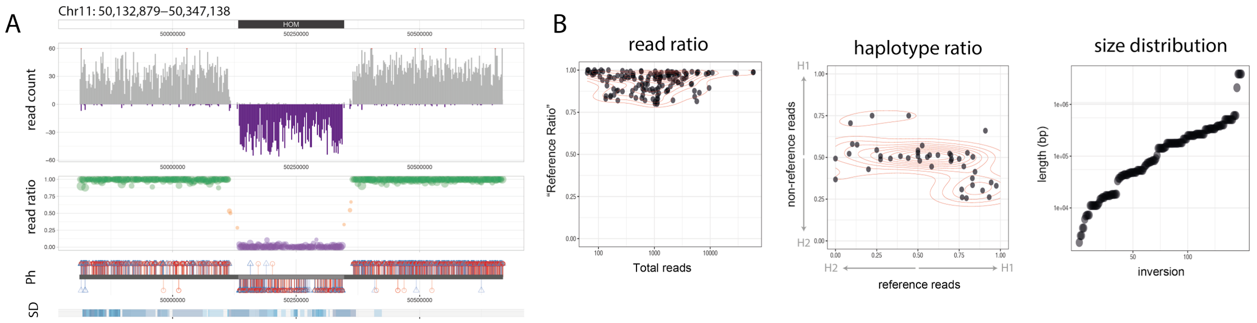


## Supplementary Figure 18.

**Haplotype structure of homozygous inversions discovered by Strand-seq.** A) Illustrative examples of a simple homozygous inversion from the Strand seq discovery set that was supported by orthogonal phase data. The upper panel (read count) displays the number of reads in the reference (grey) and 'non-reference' (purple) orientation around the highlighted locus, and the read ratio (shown below) supports a homozygous inversion. The middle panel (Ph) displays the phase of these reads, with H1 'haplotagged' reads in red, H2 reads in blue. In this panel, phased reads in the reference orientation are displayed above the ideogram, whereas phased reads in the 'non-reference' orientation are shown below (allowing for strand-aware analyses). The bottom panel (SD) highlights the location of annotated segmental duplications, with the intensity of blue indicative of the percent match. B) Scatter plots summarizing the high read ratios, mixed haplotype ratios, and size distribution of inversions classified as homozygous using this approach.

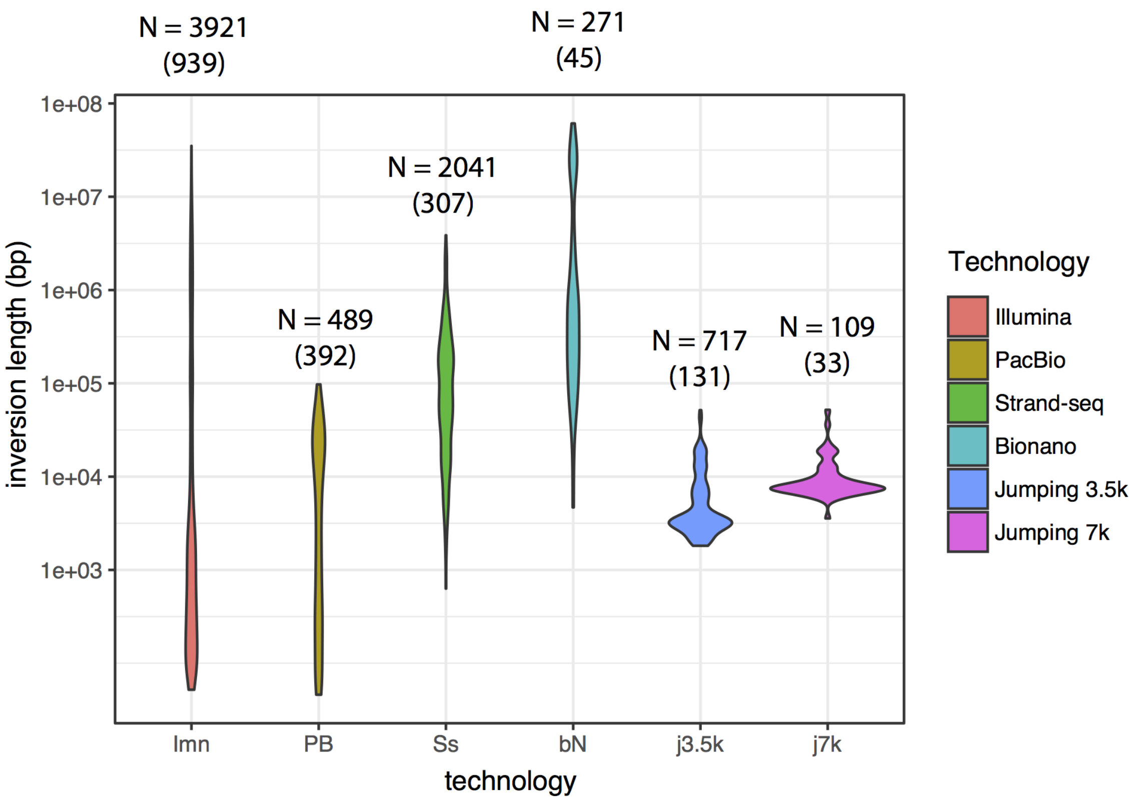


Supplementary Figure 19.

**Summary of inversion callsets included in the integration analysis.** Violin plots illustrating the size distribution of inversions predicted from various technologies, with the total number of inversion calls made for all individuals (N) listed above, and the unique number of calls in brackets.

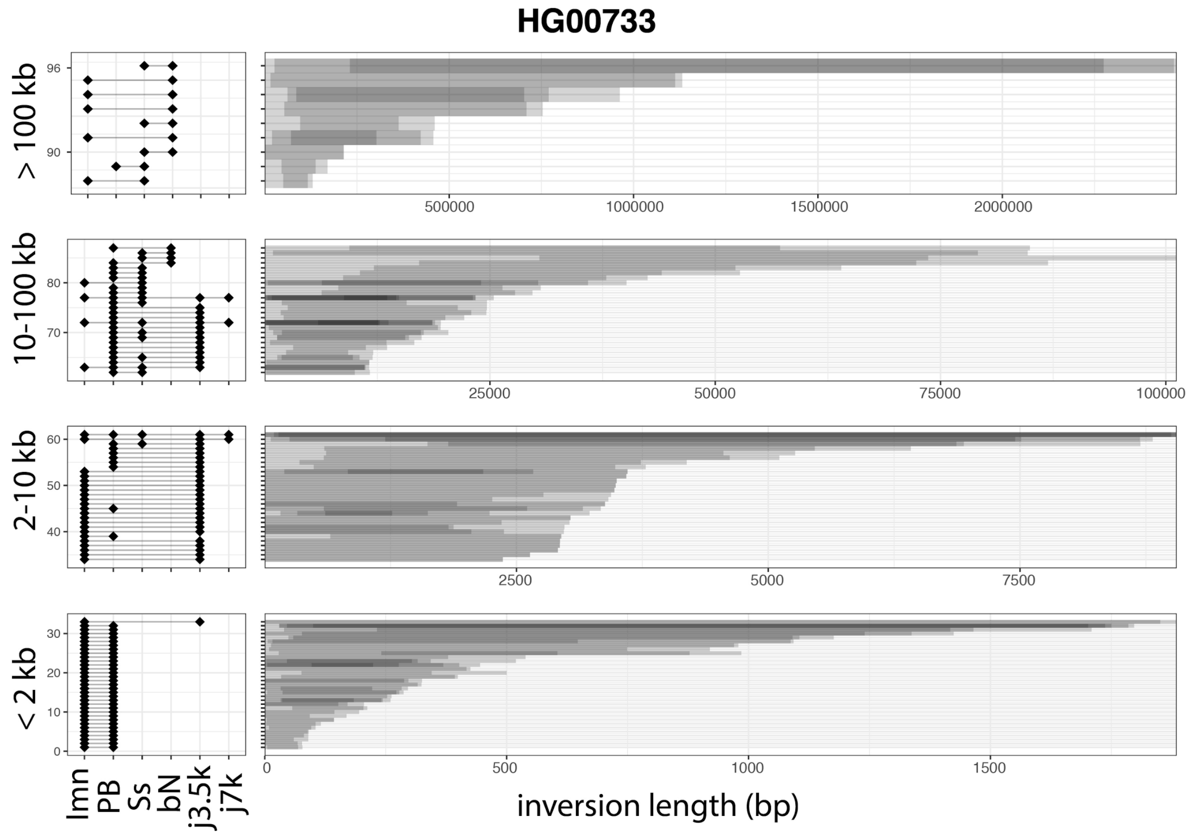


## Supplementary Figure 20.

**Intersection test results for HG00733.** Example results of inversions showing > 50% reciprocal overlap between two independent technologies, sorted by inversion size. Each horizontal bar in the right panel represents a passing inversion and depicts the level of agreement between the overlapping orthogonal platforms, with the technologies intersecting at the locus listed in the left panel. Illumina (Imn); Pacific Biosciences (PB); Strand-seq (Ss); BioNano (bN); Jumping libraries with 3.5kb (j3.5k) and 7kb (j7k) insert lengths.

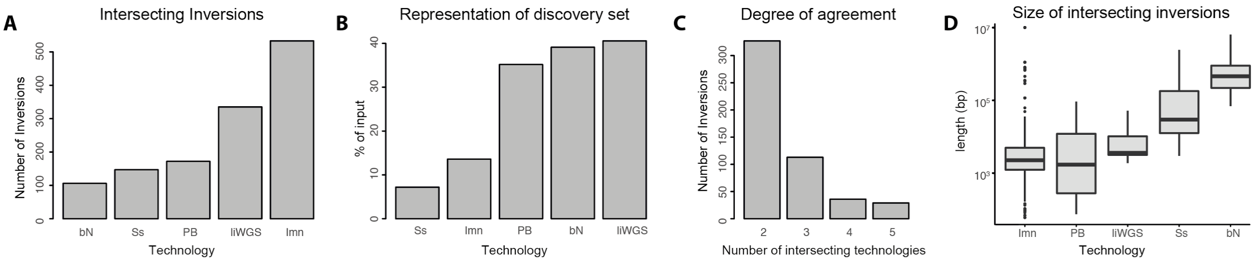


## Supplementary Figure 21.

**Summary of reciprocal overlap of inversions by method.** A) Total number of inversions showing > 50% reciprocal overlap between two independent methods, sorted by technology. B) Percent of inversions in the initial discovery callsets that passed the intersection test. C)Ssize lengths of passing inversions, sorted by technology. D) Number of methods intersecting at each inversion. Illumina (Imn); Pacific Biosciences (PB); Strand-seq (Ss); BioNano (bN); long-insert Whole Genome Sequence (a.k.a ‘jumping’) libraries (liWGS).

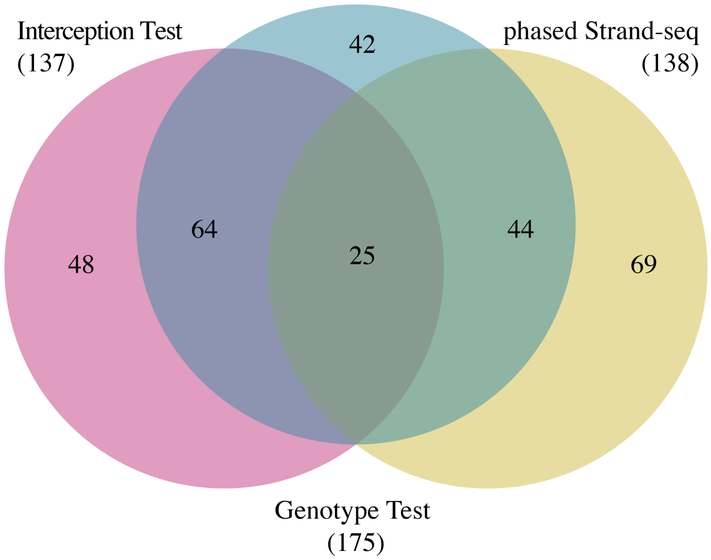


## Supplementary Figure 22.

**Inversion lists used to generate the final unified inversion callset.** Venn diagram illustrating the degree of overlap between the three support lists that were merged together to generate the unified inversion callset.

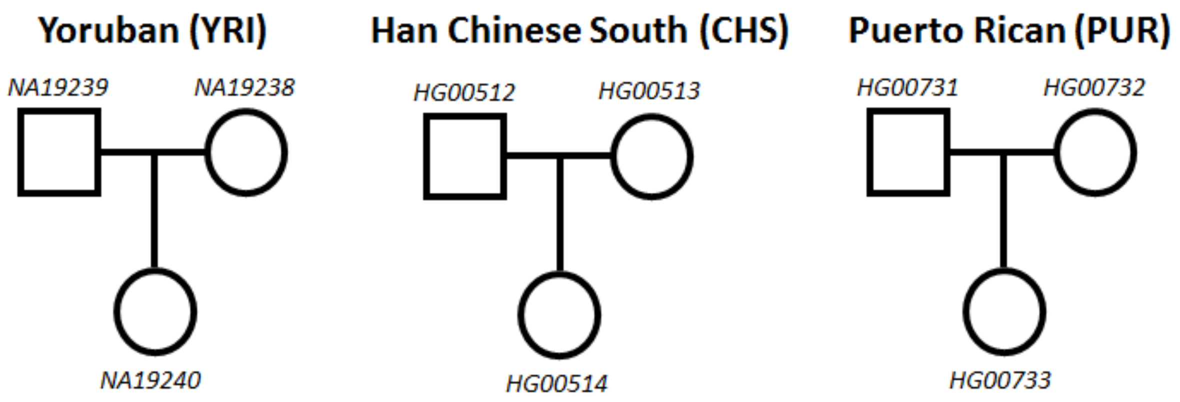


Supplementary Figure 23.

**Parent-child trios characterized for structural variation.** We refer to the children from each pedigree as YRI (Yoruban), CHS (Han Chinese South) and PUR(Puerto Rican).

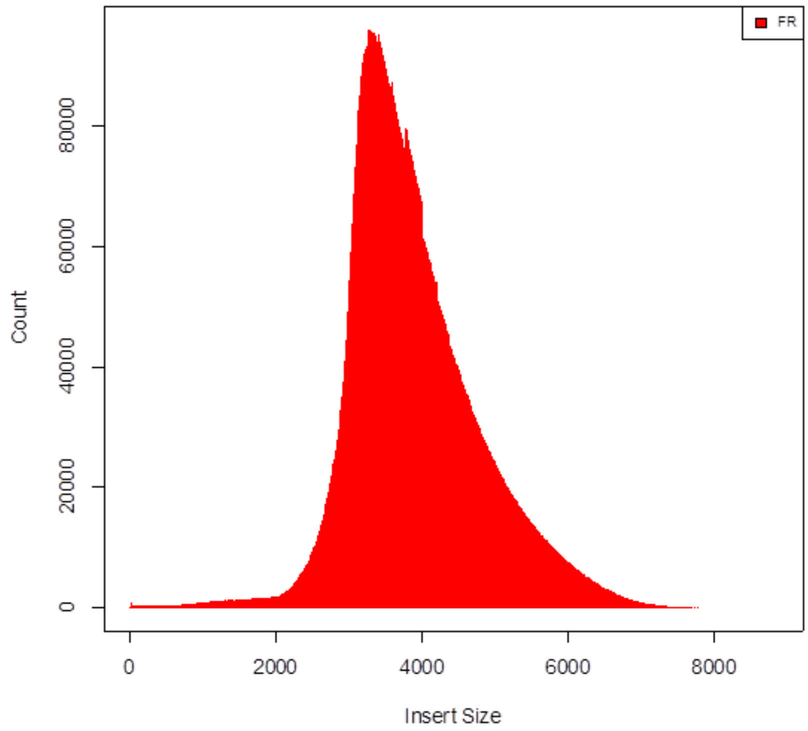


## Supplementary Figure 24.

**Insert size distribution of an example HGSVC liWGS library.** We generated liWGS libraries for all nine HGSVC individuals following previously described protocols targeting a mean insert size of 3.5kb. As an example, the distribution of insert sizes from the library generated for the father from the Puerto Rican trio is shown here. All nine libraries closely resembled this distribution. This distribution was generated automatically by Picard tools (http://broadinstitute.github.io/picard/).

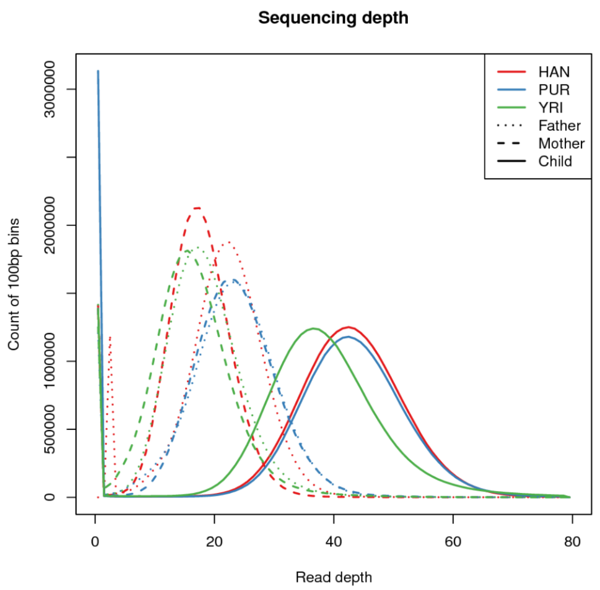


## Supplementary Figure 25.

SMRT sequence coverage.

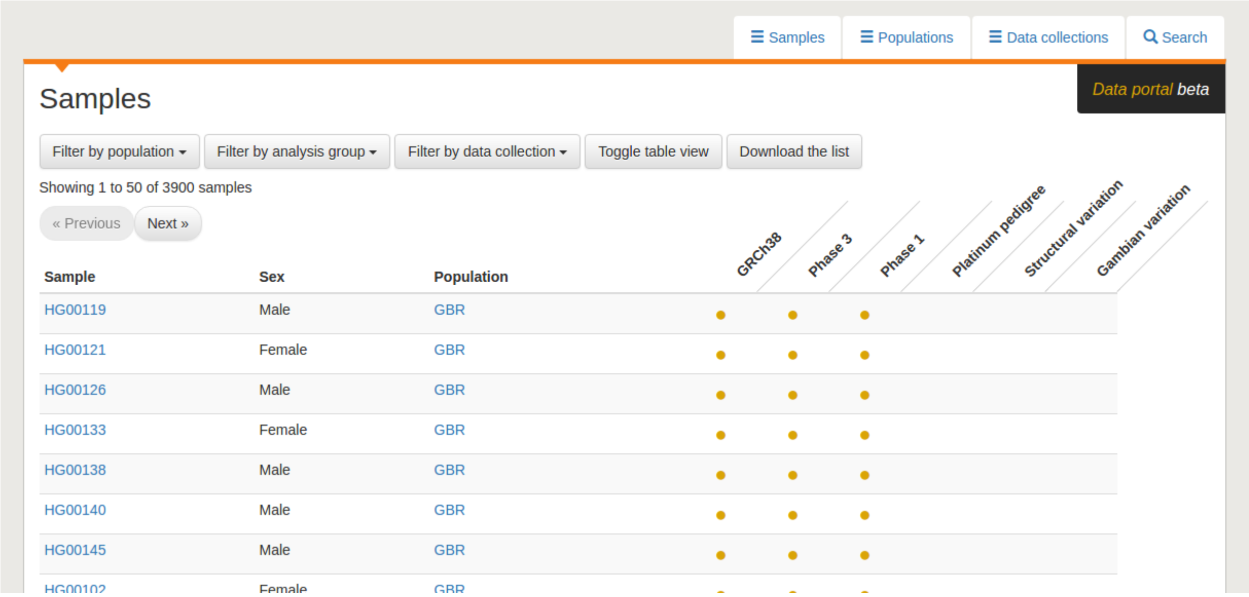


## Supplementary Figure 26.

**Data portal for IGSR samples.** To assist in locating data, IGSR has created a data portal, which includes the major data collections hosted by the project. A full description of IGSR, including the data portal is available in (Clarke et al. 2017). A page summarising data from the HGSVC can be found at http://www.internationalgenome.org/data-portal/data-collection/structural-variation.

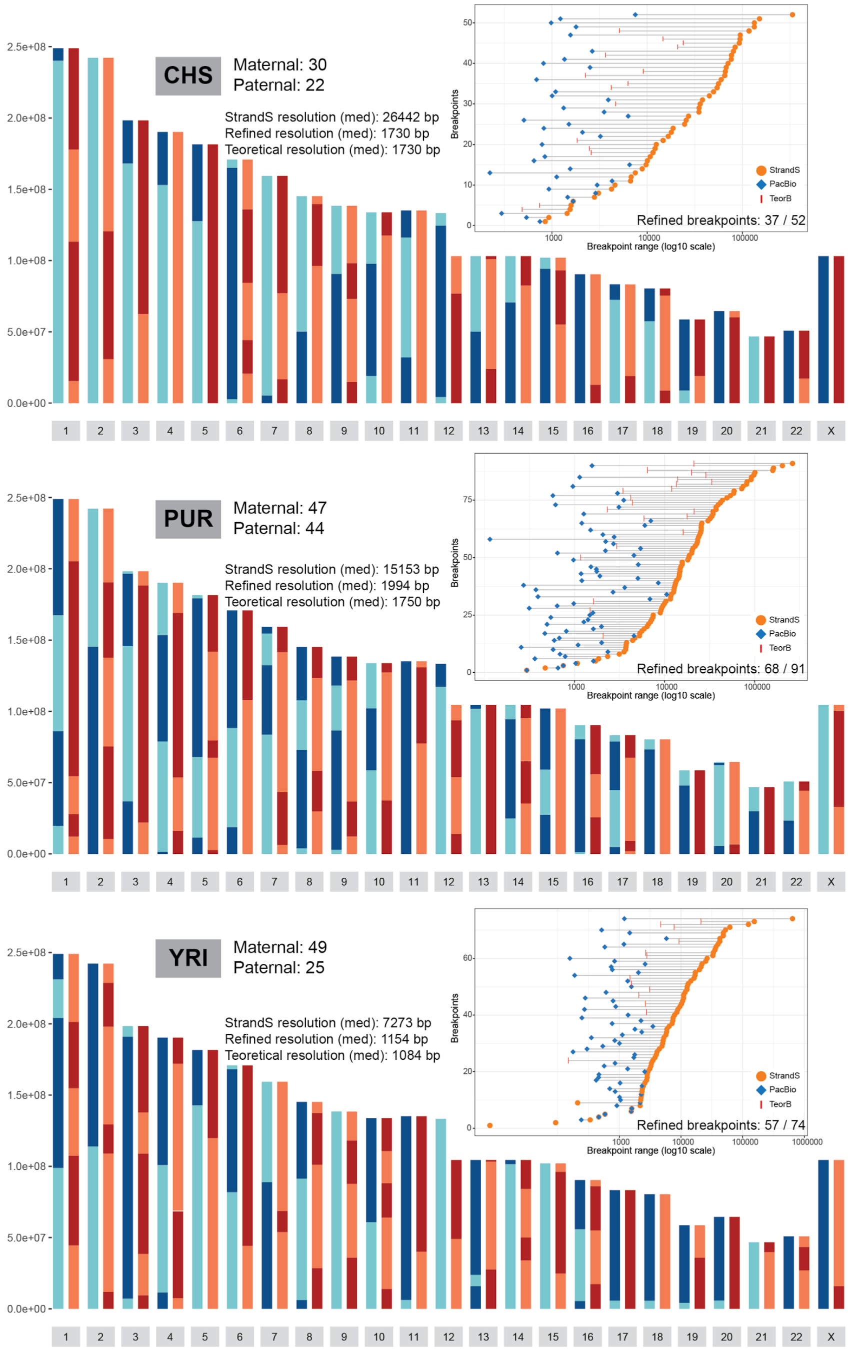


## Supplementary Figure 27.

**Map of meiotic recombination events for each family trio.** Ideograms show map of meiotic breakpoints for each chromosome with inherited parts of paternal (Paternal homologue H1 – light blue, Paternal homologue H2 – dark blue) and maternal (Maternal homologue H1 – light red, Maternal homologue H2 – dark red) homologues. Inset figure in the right upper corner of each ideogram shows the size distribution of mapped meiotic recombination breakpoints using Strand-seq (yellow dots) and corresponding refined breakpoints using PacBio reads (blue squares) connected by a line. Meiotic breakpoints that in theory could be further refined by residual HET SNVs within the breakpoint are shown reb bar (TeorB).

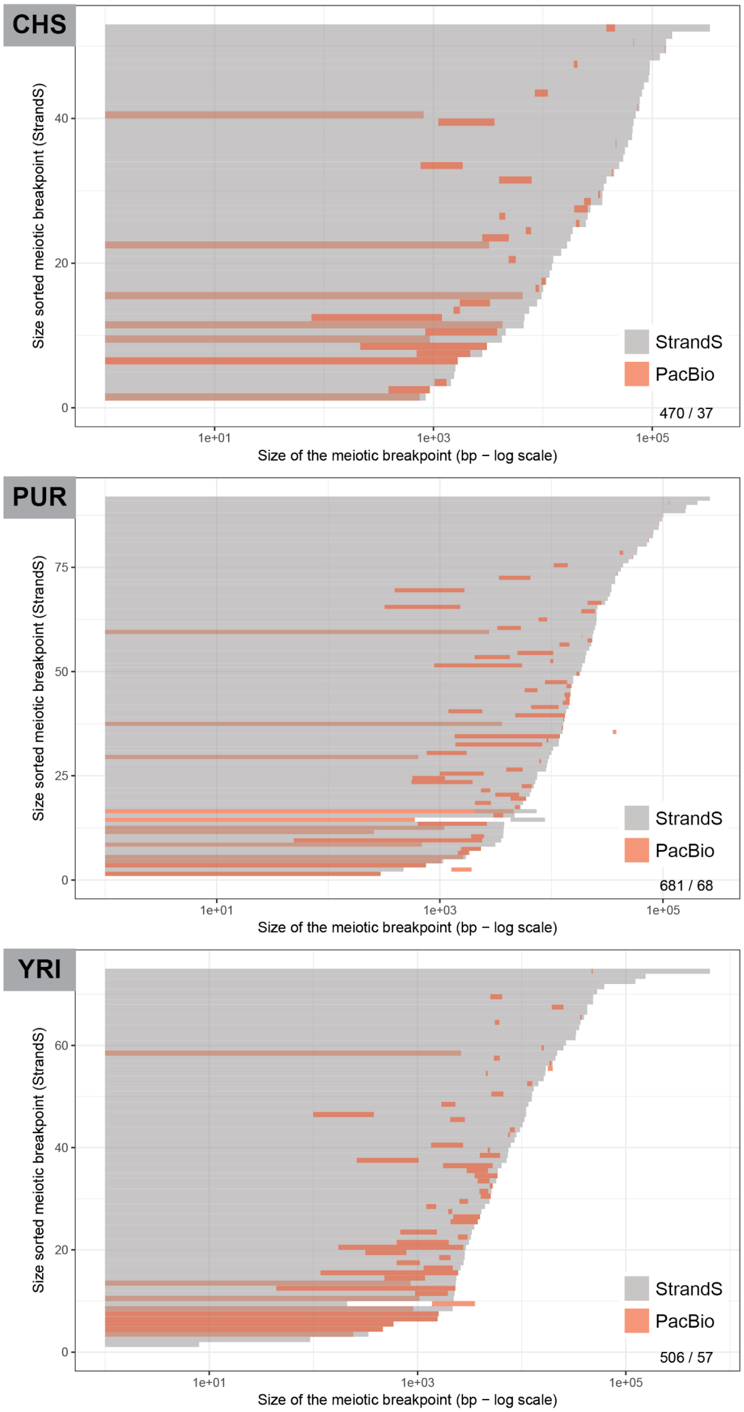


## Supplementary Figure 28.

**Level of overlap between Strand-seq and PacBio derived meiotic breakpoints.** Figure shows an overlap between meiotic breakpoints mapped by Strand-seq and breakpoints predicted from PacBio reads. Strand-seq meiotic breakpoints are shown in gray color and are sorted by size. Overlapping breakpoints predicted from PacBio reads are depicted in red color. Number of overlapping breakpoints from the total number of breakpoints predicted from PacBio reads is shown in the lower right corner of each plot.

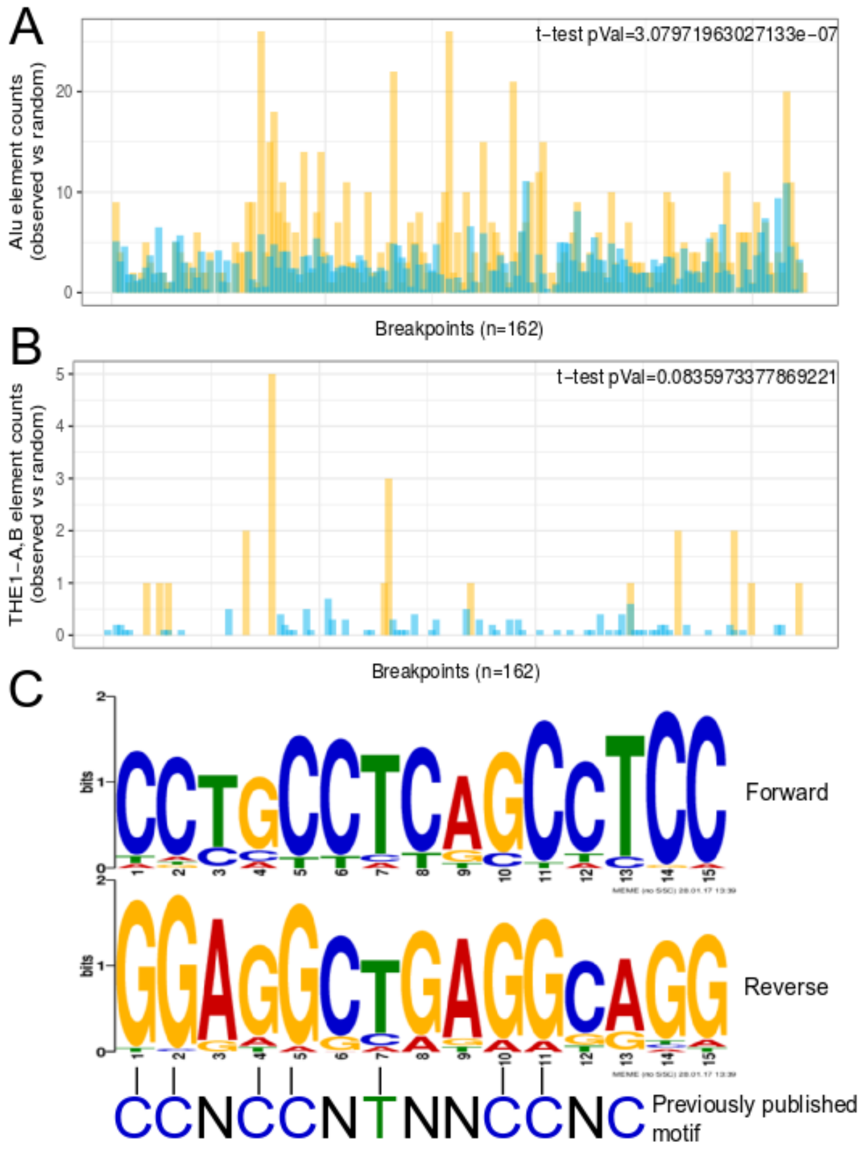


## Supplementary Figure 29.

**Enrichment analysis of Alu and THE1-A,B elements around mapped meiotic breakpoints.** Figure shows raw counts of Alu and THE1-A,B elements overlapping with our fine mapped meiotic breakpoints (n=162). Mapped breakpoint ranges were scaled to the largest mapped range (~10 kb). To test if the the above mentioned elements are enriched around meiotic breakpoints we have compared their counts for observed versus random meiotic breakpoints. To simulate random meiotic breakpoints in the genome we have randomly shuffled our mapped meiotic breakpoints around the chromosome they originate from. We have performed 10 independent trials and presented randomized counts represent mean values of all trials. We used t-test statistics to compare Alu and THE1-A,B elements counts between observed and random breakpoints.

a. Distribution of Alu elements around the breakpoints versus randomized breakpoints.

b. Distribution of THE1-A,B elements around the breakpoints versus randomized breakpoints.

c. Consensus motif found in fine mapped meiotic breakpoints (n=162) using MEME suite (zoops mode). Motif significance level: E-value = 3.1e-179. Motif is compared to the previously published motif by Myers et al. (2008).

Note: Genomic positions of Alu and THE-1A,B elements were taken from the 'GRCh38 RepeatMasker track' from UCSC genome browser.

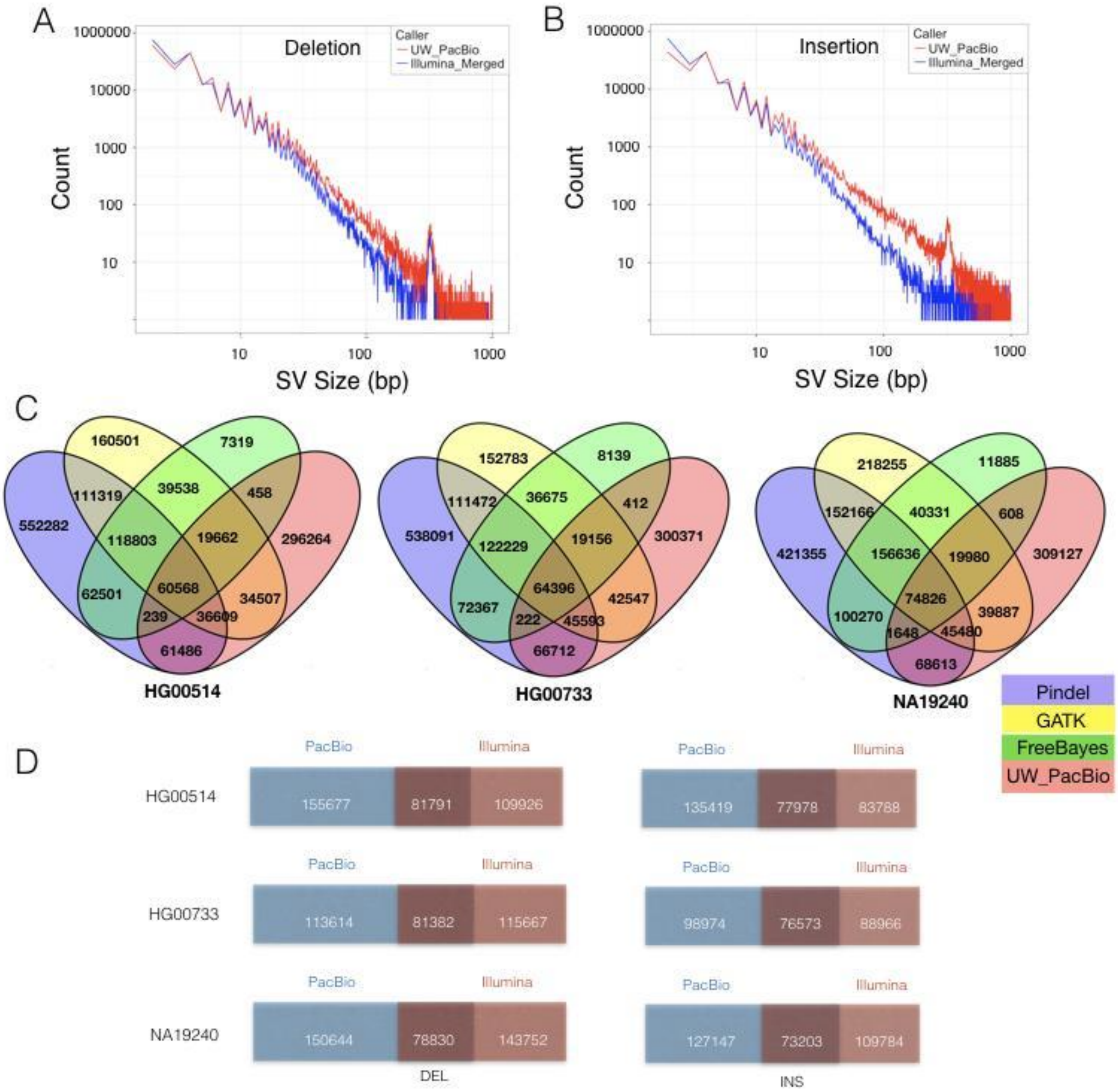


E

| Proband | Caller | DEL | INS |
|---|---|---|---|
| HG00514 | FreeBayes | 160381 | 148707 |
| | GATK | 305509 | 275998 |
| | Pindel | 551417 | 452390 |
| | UW_PacBio | 157413 | 141896 |
| HG00733 | FreeBayes | 169348 | 154248 |
| | GATK | 312359 | 282492 |
| | Pindel | 562056 | 459026 |
| | UW_PacBio | 133196 | 104170 |
| NA19240 | FreeBayes | 218569 | 187615 |
| | GATK | 393464 | 354097 |
| | Pindel | 564226 | 456768 |
| | UW_PacBio | 146723 | 132487 |

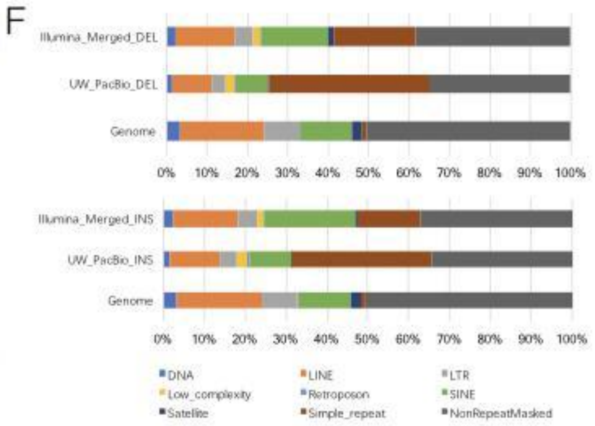


## Supplementary Figure 30.

**Indel discovery summary.** Deletions and insertions are merged from GATK, Pindel and FreeBayes calls to give Integrated Illumina deletions and insertions respectively, which is then compared to PacBio calls. A. Size frequency distribution of merged Illumina deletions alongside PS/MP (UW_PacBio) deletions from 1bp to 1kb. B. Size frequency distribution of merged Illumina insertions alongside UW_PacBio insertions from 1bp to 1kb. C. Four-way Venn diagrams of indels (1-

49bp) from GATK, Pindel, FreeBayes and UW_PacBio callsets for three children: HAN (HG00514), PUR (HG00733) and YRI (NA19240). D. Comparison of UW_PacBio and Illumina integrated indels (1-49bp). E. Table summarizing the number of deletions and insertions called by different methods for the three children. F. Stacked bar graph summarizing the proportion of deletions and insertions residing in various types of repeat regions and Non-repeat-masked region. “Genome” indicates the background proportion of repeat content in the human genome.

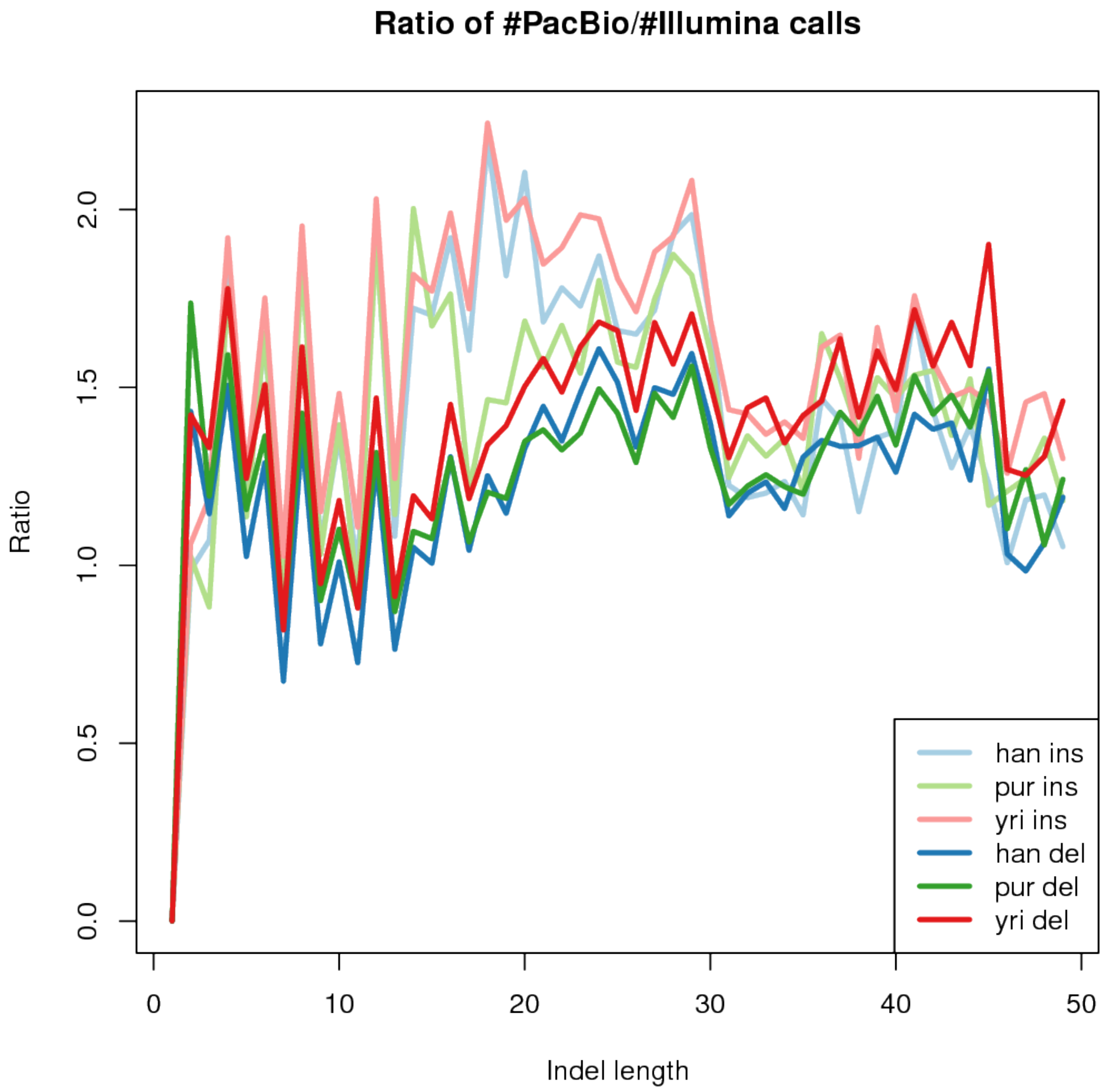


Supplementary Figure 31.

**The number of insertions and deletions detected at each size from 0 to 49 bp were compared across all samples.**

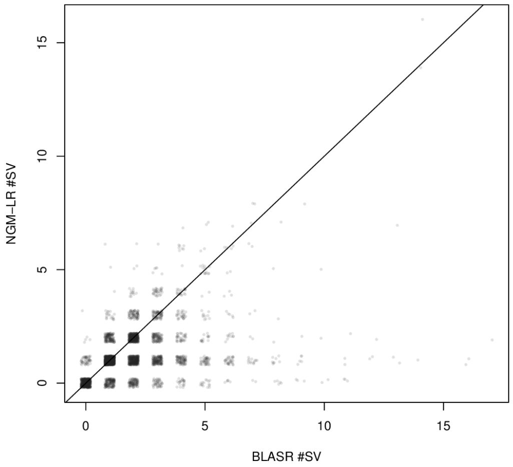


## Supplementary Figure 32.

**BLASR and NGM-LR alignment comparisons.** The SV counts from BLASR and NGM-LR, with jitter applied to distinguish different densities of points.

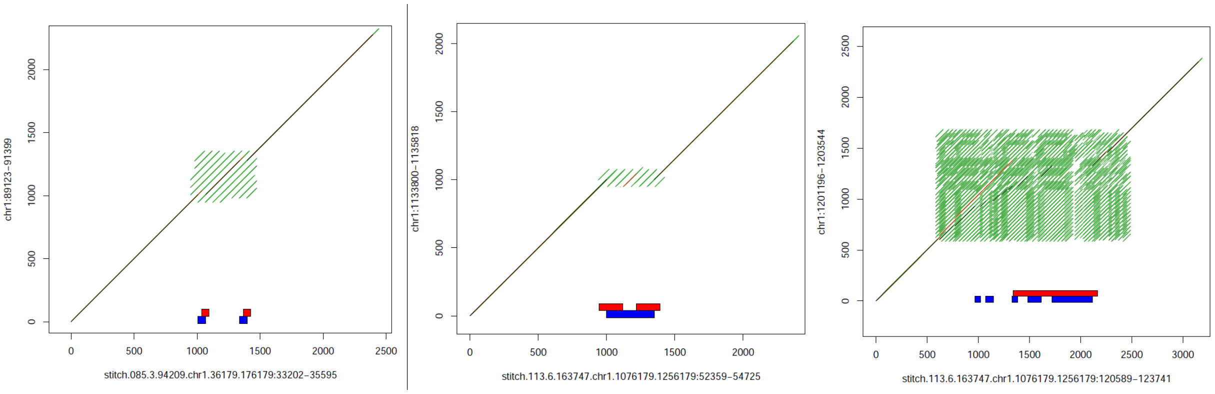


## Supplementary Figure 33.

**Dotplot comparison of alignments in tandem repeat regions.** Examples of tandem repeat loci where (top left) both alignment methods detect two SVs, (top right) NGM-LR detects more SVs than BLASR, and (bottom left), BLASR detects more SVs than NGM-LR.

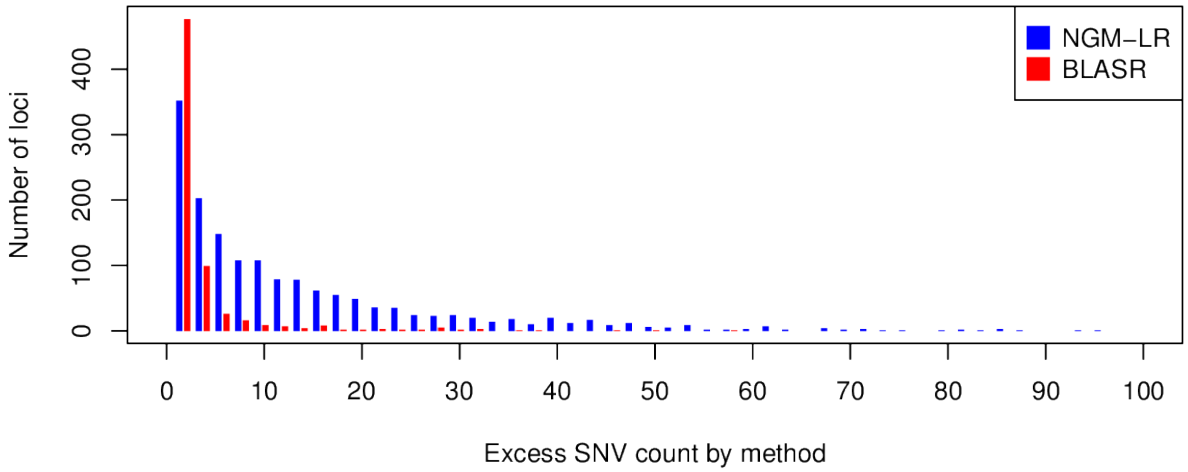


## Supplementary Figure 34.

**Excess SNV count by alignment method.** Excess SNV count by method. For each TR locus in HG00514, haplotype 0, the number of times, and by how many one method exceeds the other in SNV count.

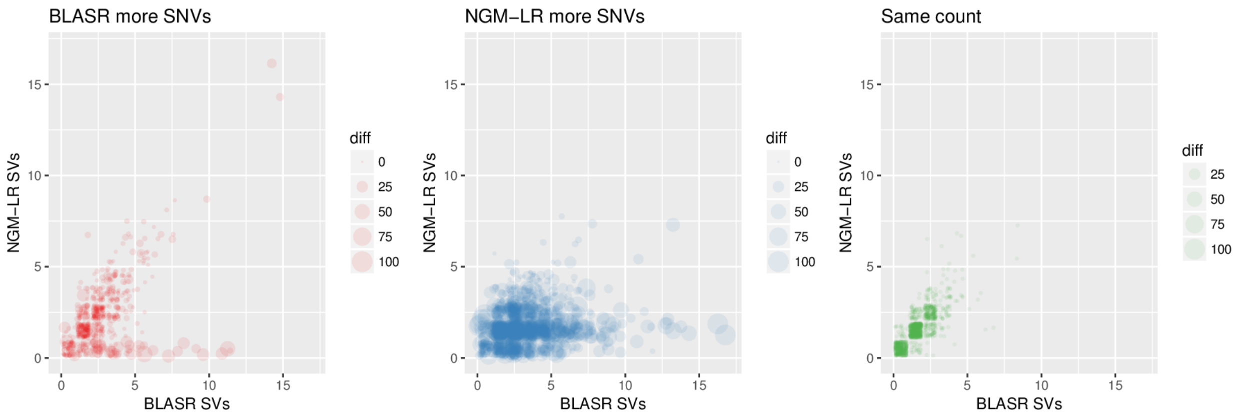


## Supplementary Figure 35.

**Detailed view of excess SNV count by alignment method.** A comparison of excess SNV count by method and number of SVs detected at each locus.

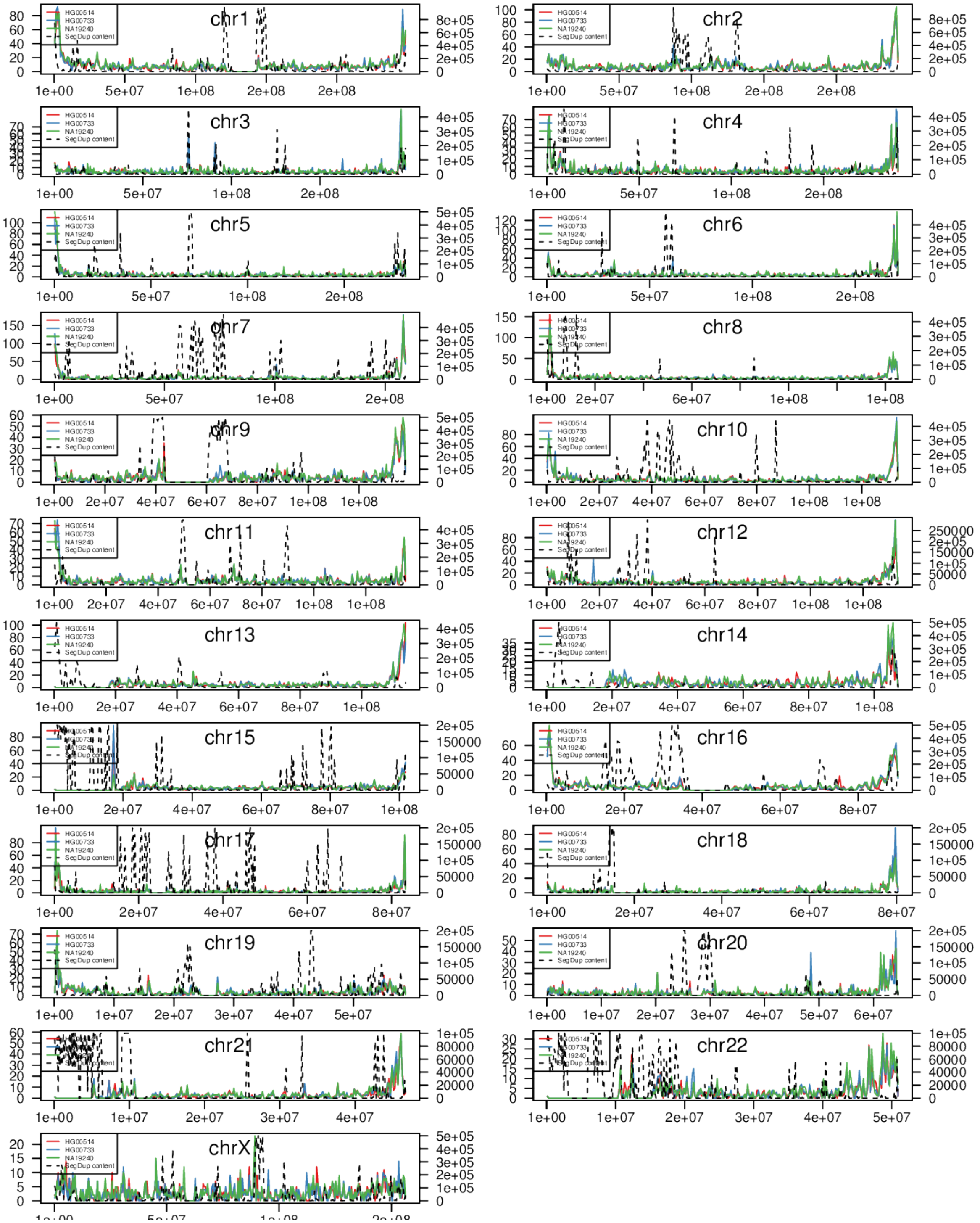

## Supplementary Figure 36.

**Genomic distribution of SVs.** The number of segmentally duplicated bases per 100 kbp is plotted along with the number of bases of segmental duplication per 100 kbp for the children of each trio. The largest association of SV is with telomeres, and not segmental duplication.

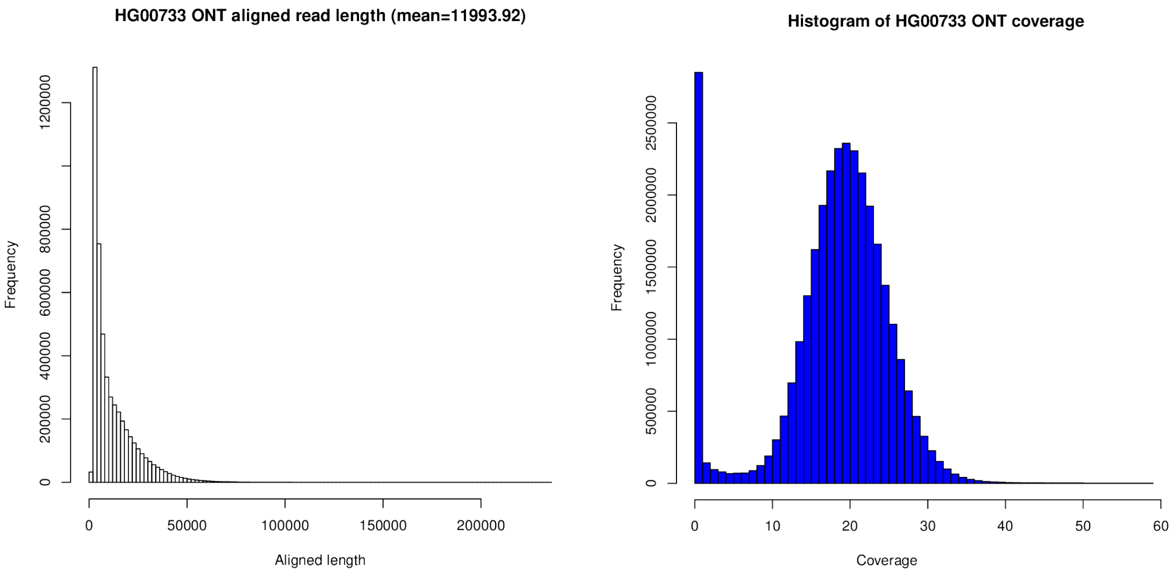


## Supplementary Figure 37.

**Oxford Nanopore sequencing read length and coverage for HG00733.**

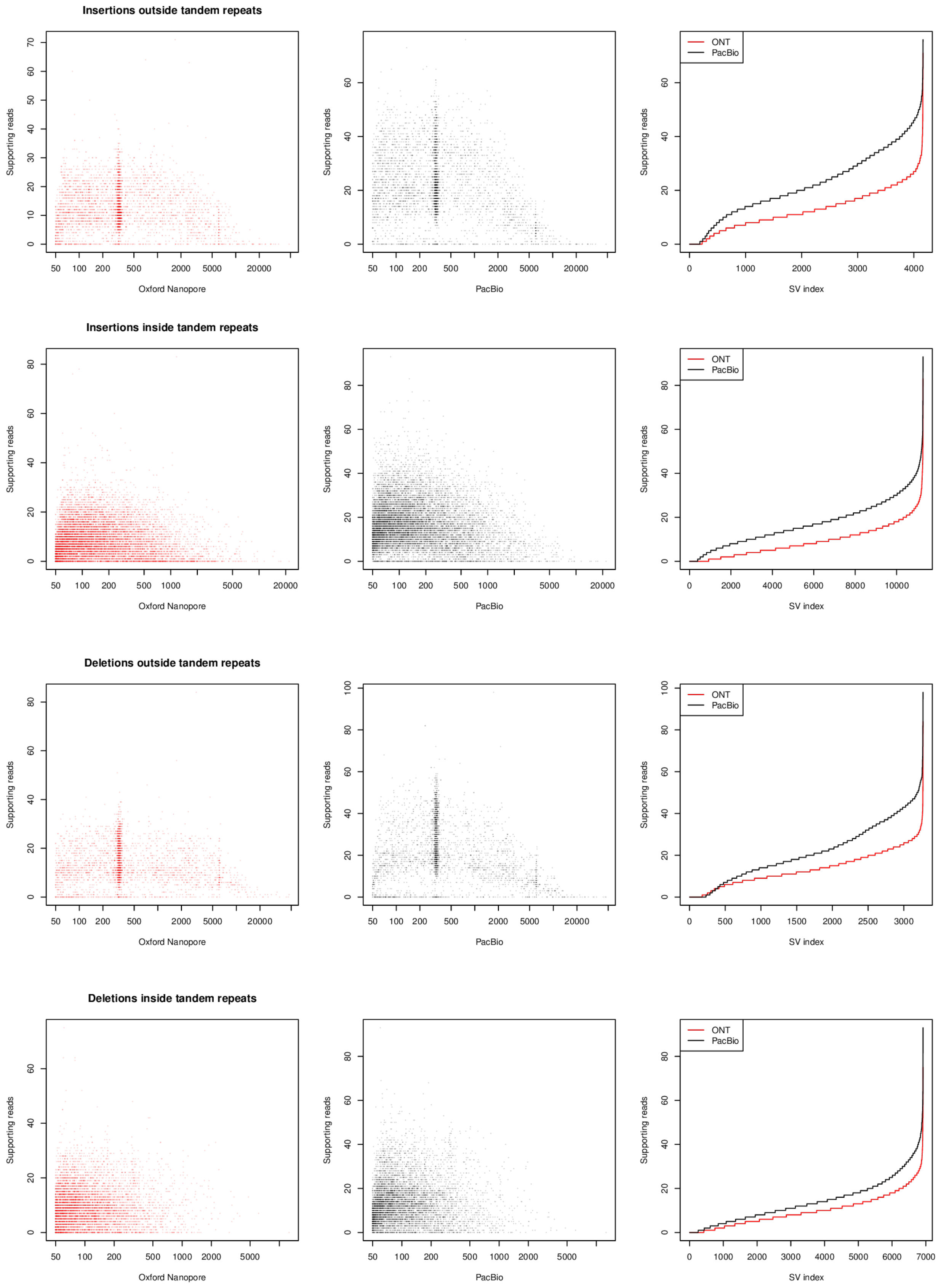


## Supplementary Figure 38.

**Read support for SV calls for HG00733 from (red) ONT and (black) PB reads.** Support for each type of SV (insertion and deletion) is shown inside and outside tandem repeat loci.

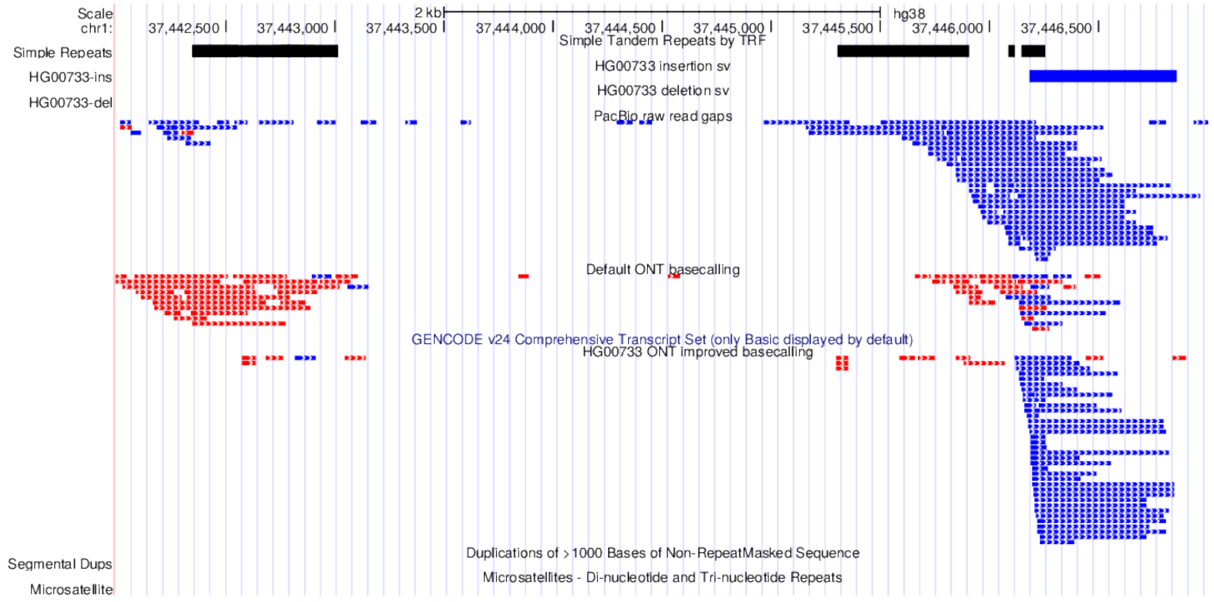


## Supplementary Figure 39.

**Example of ONT SV detection with different base calling parameters.** (top track) tandem repeat annotations, (HG00733 insertion SV, HG00733 deletion SV) insertion and deletion calls from the HGSVC Phased-SV+MSPAC calls, (PacBio raw read gaps track) SV calls detected in raw PacBio reads. Blue is insertion (start+length), and red is deletion. While the insertion calls are spread out, they are largely of consistent length, (Default ONT basecalling) SVs detected within reads from the default ONT base calling (Defau alt ONT basecalling track) where reads support the 3’ insertion, and many reads show disperse deletions over the 5’ tandem repeat, (HG00733 ONT improved basecalling) SVs detected within the same reads but recalled with updated basecalling parameters. The insertions are of more consistent size and count, and now support the insertion even more consistently than then PacBio reads.

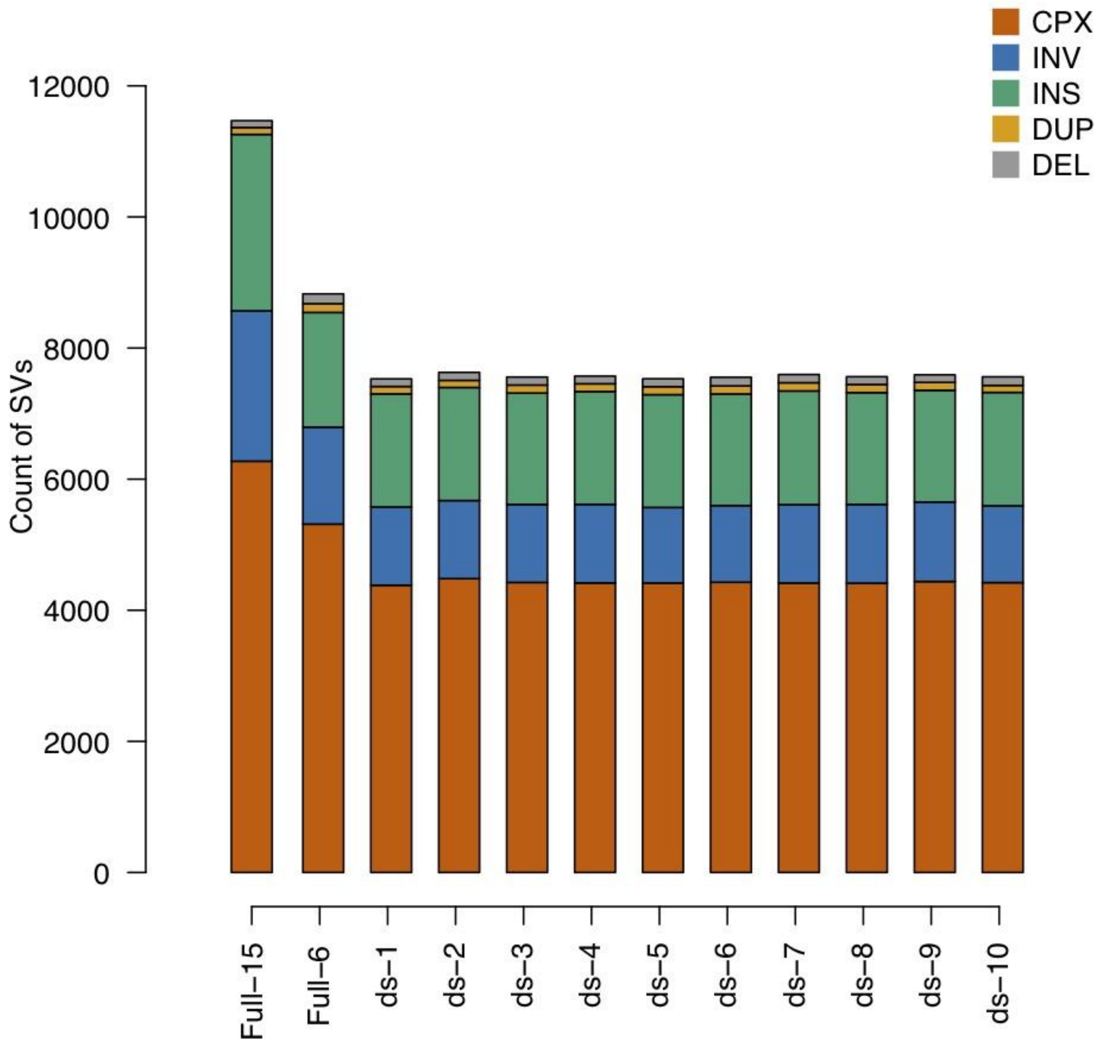


Supplementary Figure 40.

**Overview of SVs discovered with all algorithms and a subset of algorithms on full coverage (75X) and down sampled (30X) datasets.** Full-15 = Callset integrated from 15 SV discovery algorithms on 75X Illumina whole genome sequences (WGS); Full-6 = Callset integrated from 6 algorithms, i.e. Delly, Lumpy, Manta, MELT, Wham, SVelter, on full coverage Illumina WGS; ds-1 = callset integrated from 6 algorithms on 30X WGS down sampled with seed 1.

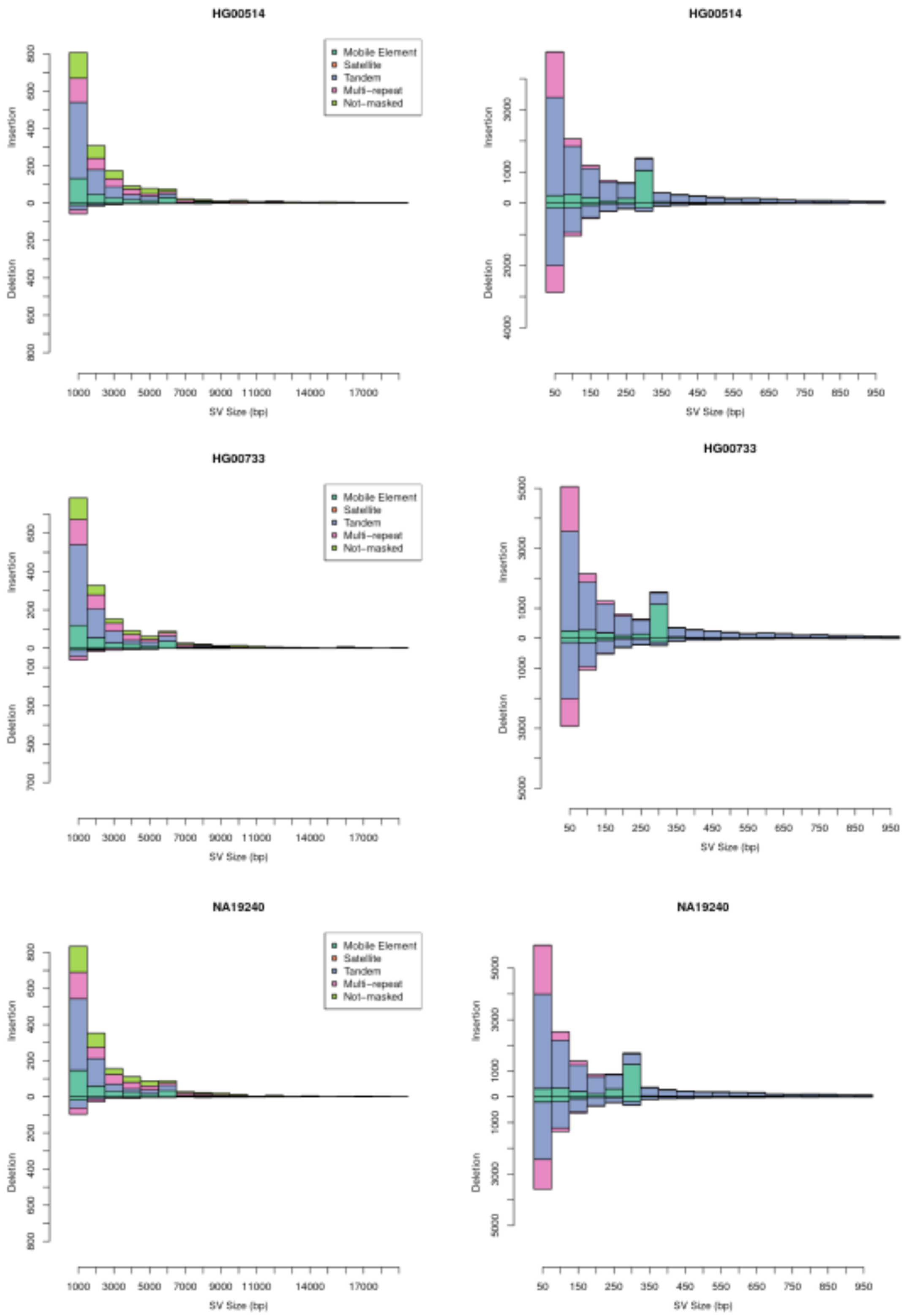


## Supplementary Figure 41.

**The counts of SVs affecting genes by method and region of gene.**

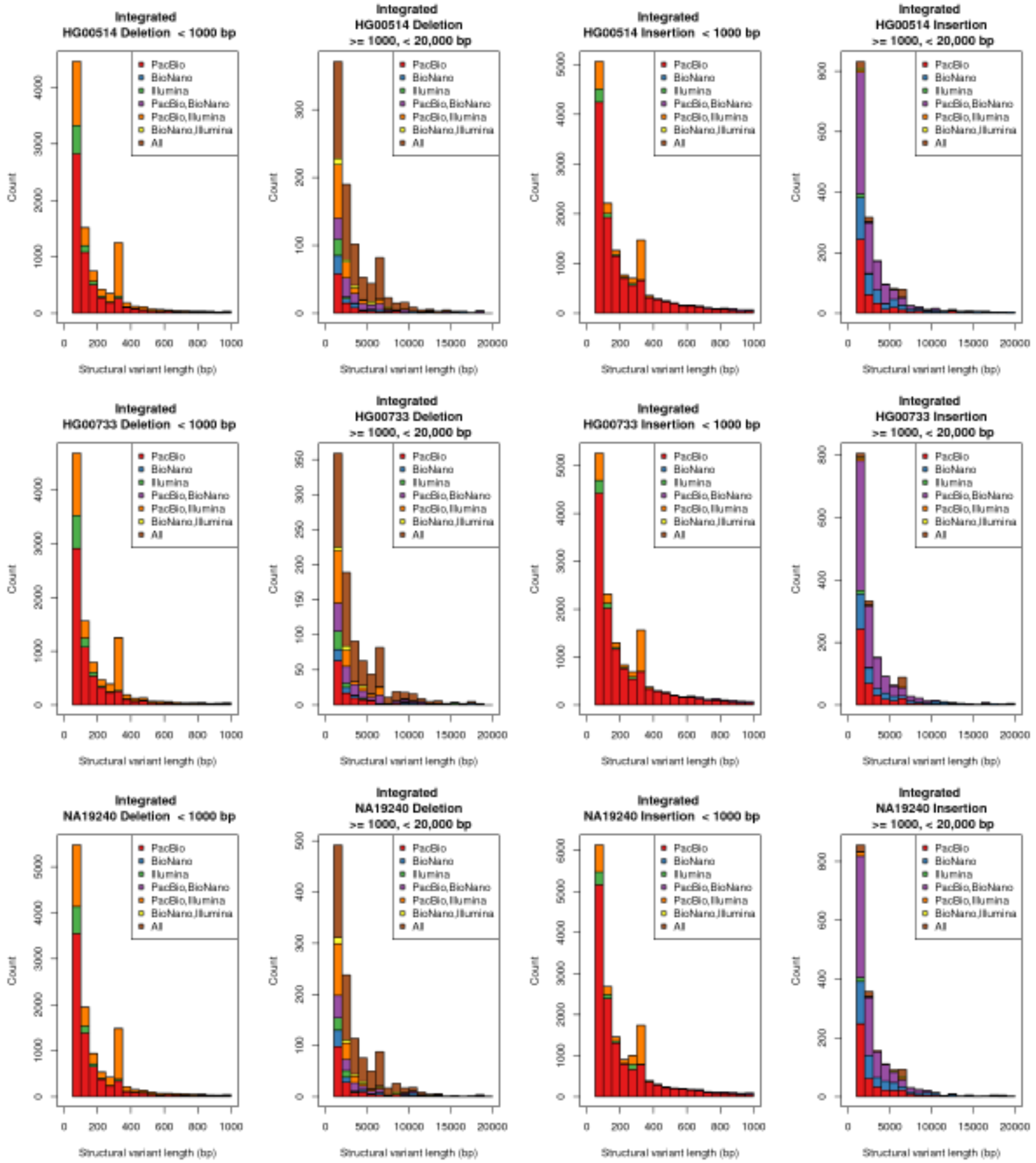


## Supplementary Figure 42.

**Regions of SV that were called in the IL-SV callset and not PB-SV.** PB-SV only calls are shown for the YRI child.

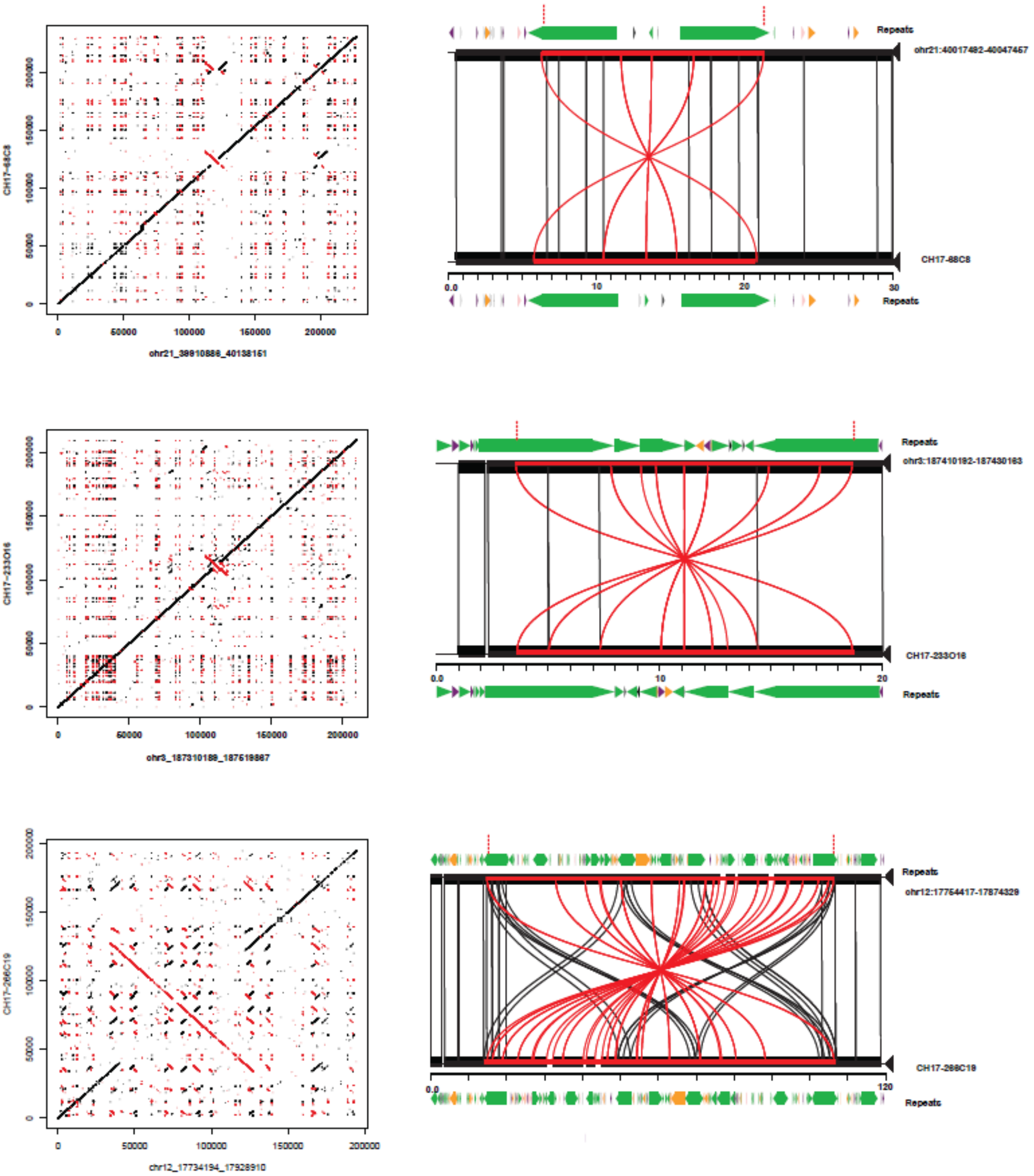

CH17-88C8
chr21_39910886_40138151
Repeats
chr21:40017492-40047457
CH17-88C8
Repeats
CH17-233O16
chr3_187310189_187519887
Repeats
chr3:187410192-187430163
CH17-233O16
Repeats
CH17-266C19
chr12_17734194_17928910
Repeats
chr12:17754417-17874329
CH17-266C19
Repeats

b)

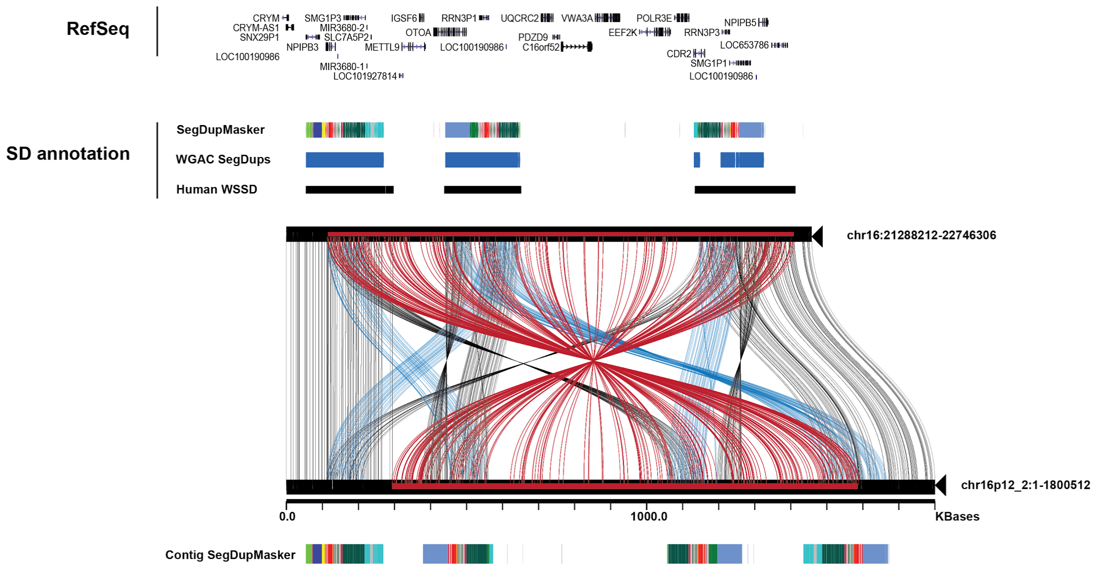


c)

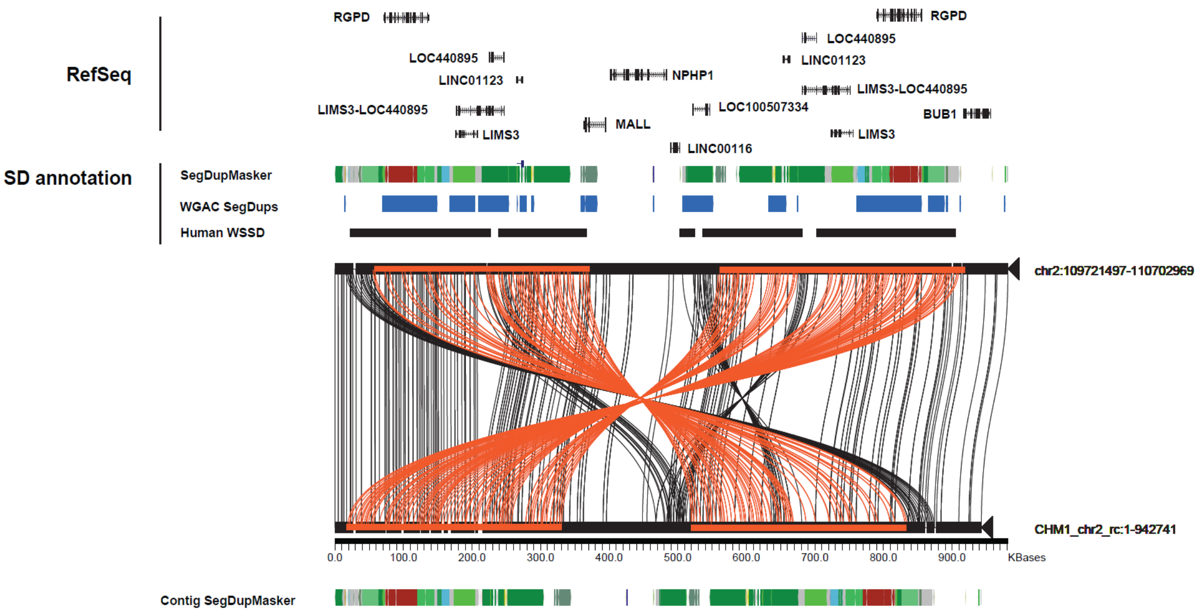


## Supplementary Figure 43.

**Misassemblies in the GRCh38 reference validated by large insert clone based sequence and assembly.** Inversions detected by strand seq followed by sequence resolution using CH17 BAC clones. Inversions are validated by by aligning the corresponding BAC sequences to GRCh38 and visualized using Miropeats (Parsons 1995) and dot plots. The miropeats figures depict black lines indicating homologous sequence between the two assemblies and red lines correspond to inversion events. RepeatMasker annotation demonstrates that inversion events are flanked by inverted LINE/L1 repeats (green). **b) An ~857 kbp sequence-resolved inversion on chromosome 16p12.1.** Sequence and assembly of 16 CHM1 BAC clones to generate a ~1.8 Mbp alternate reference haplotype corresponding to chr16p12. A miropeats comparison between the alternate reference and the GRCh38 reference depicts a large inversion (red lines) and additional expansions of segmental duplication blocks (blue). Annotations include whole-genome shotgun sequence detection (WSSD) (Bailey et al. 2002), DupMasker (Jiang et al. 2008) and Refseq annotations. **c) An 842 kbp chr2q13 alternate reference haplotype consistent with the GRCh38 reference assembly.** Sequence and assembly of 6 CHM1

BAC clones to generate a ~843 kbp alternate reference haplotype corresponding to chr2q13. A miropeats comparison depicts large highly identical segmental duplication blocks (~358 kbp) mapping in inverted orientation (orange lines) flanking ~120 kbp of unique sequence. The alternate reference haplotype confirms the order and orientation of the GRCh38 reference assembly indicating that CHM1 represents the minor reference haplotype at this locus. Annotations include whole-genome shotgun sequence detection (WSSD) (Bailey et al. 2002), DupMasker(Jiang et al. 2008) and Refseq annotations.

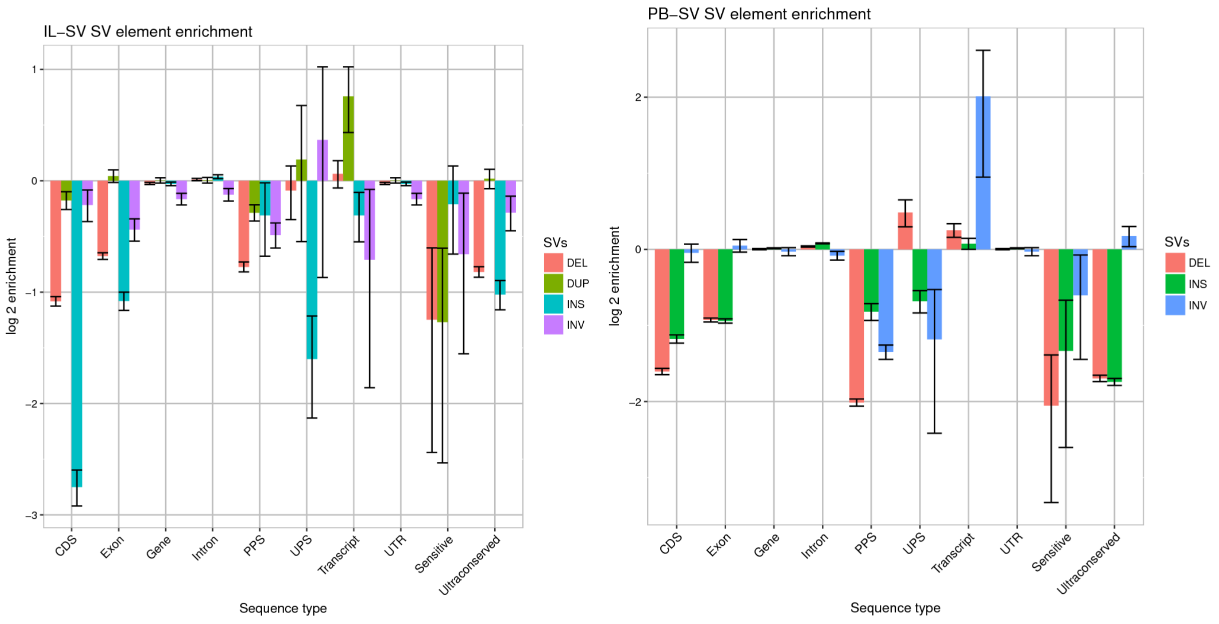


## Supplementary Figure 44.

**Enrichment for SVs intersecting functional elements in the IL-SV and PB-SV callsets.** CDS: coding sequences, PPS: processed pseudogenes, UPS: unprocessed pseudogenes.

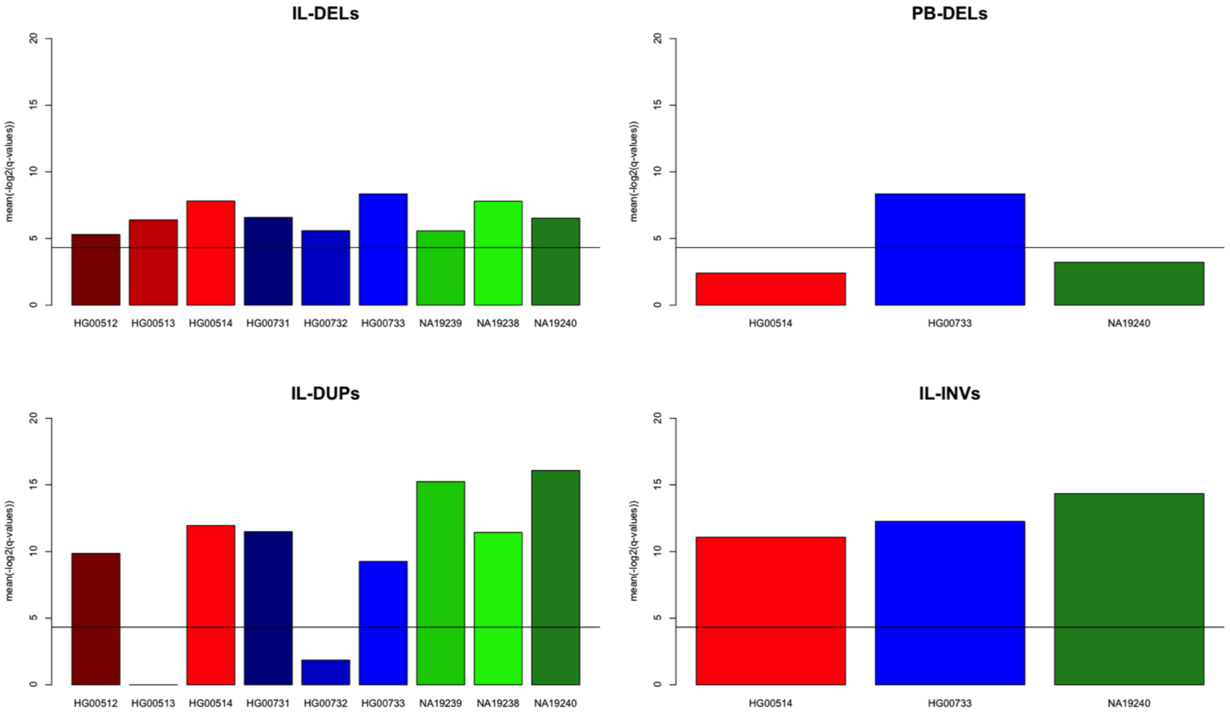


## Supplementary Figure 45.

**Significance of gene expression effect by PB-SVs and IL-SVs that engulf protein coding genes.** Samples are given along the x-axis of each plot while vertical bars depict the average –log2(q-values) calculated from the group t-tests between the RPKM normalized expression values of genes engulfed by structural variants and that of genes engulfed by permuted chromosomal regions. Panel A. shows results for the integrated Illumina deletions (IL-DELs) for all 9 individuals, while Panel B. gives the results for PacBio deletions (PB-DELs) in trio daughters. Panel C. illustrates the results from the analysis of the integrated Illumina duplications (IL-DUPs) for the 9 samples, and Panel D. shows results from the analysis of Illumina inversions (IL-INVs) engulfed genes for the trio daughters. The position of the horizontal line in each panel corresponds to the significance threshold (q = 0.05).

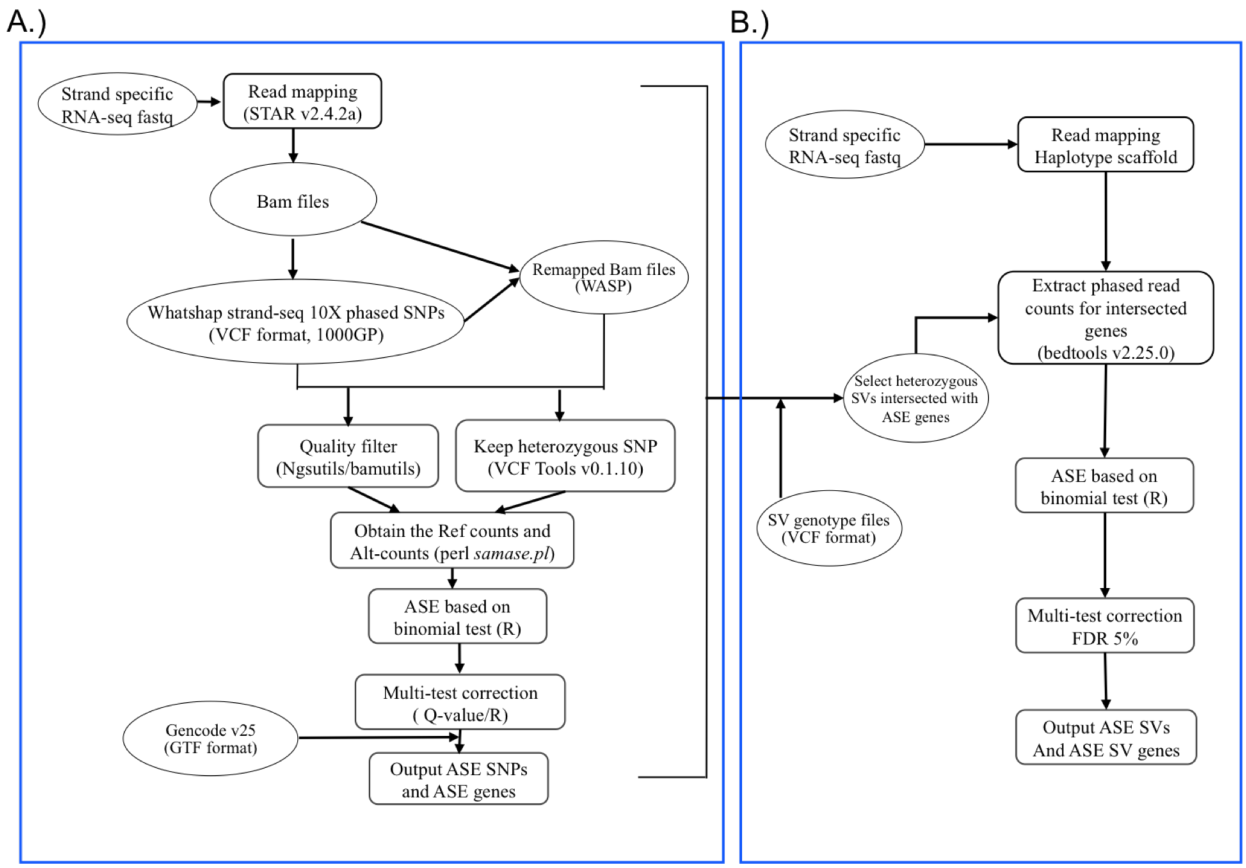


## Supplementary Figure 46.

**A pipeline of the allele specific expression analysis for SNPs and SNVs.**

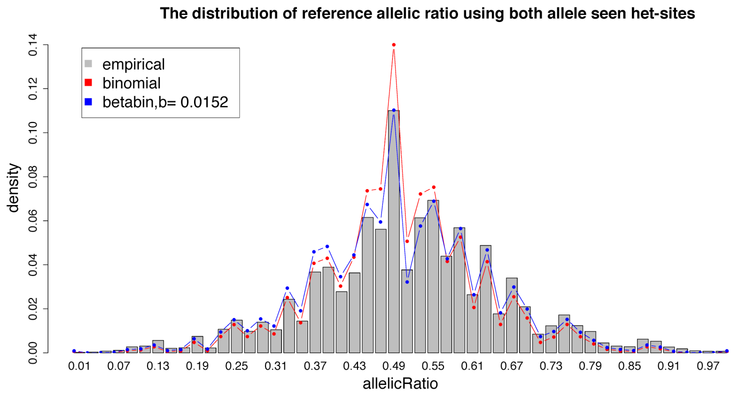


## Supplementary Figure 47.

**The distribution of reference allelic ratio using both allele seen het-SNPs.** Grey bars shows the empirical reference allelic ratio distribution. Red and blue lines show the null expected allelic ratio distributions associated with the binomial and beta-binomial test, respectively. The overdispersion b is as low as 0.0152 which would give similar results for binomial and beta-binomial tests.

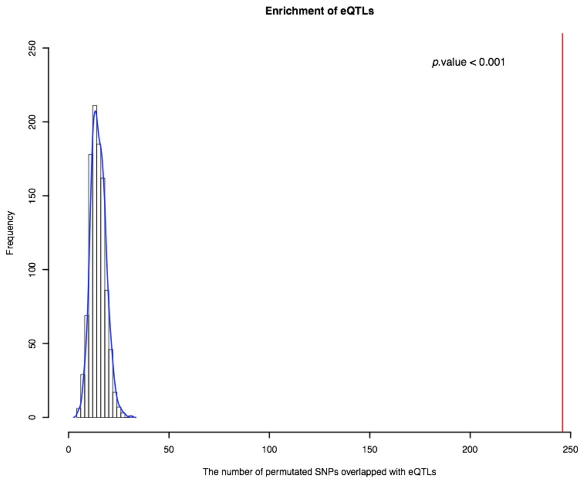


## Supplementary Figure 48.

**Enrichment analysis for eQTLs.** The red line denotes the 246 ASE SNPs identified to overlap with eQTLs; the bars denote the number of SNPs overlapping with eQTLs in each permutation test.

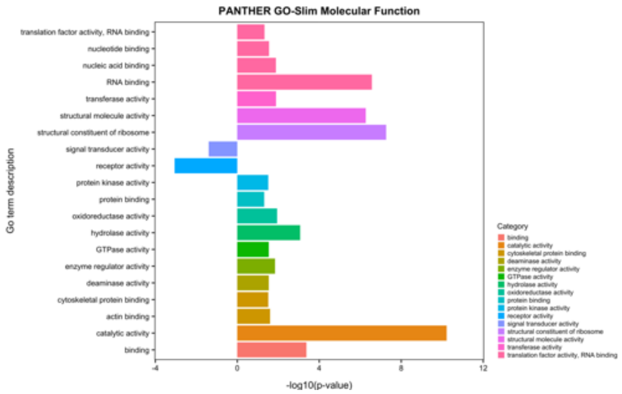


## Supplementary Figure 49.

**Molecular functions enriched for ASE genes.** A positive value denotes enrichment and a negative value denotes depletion.

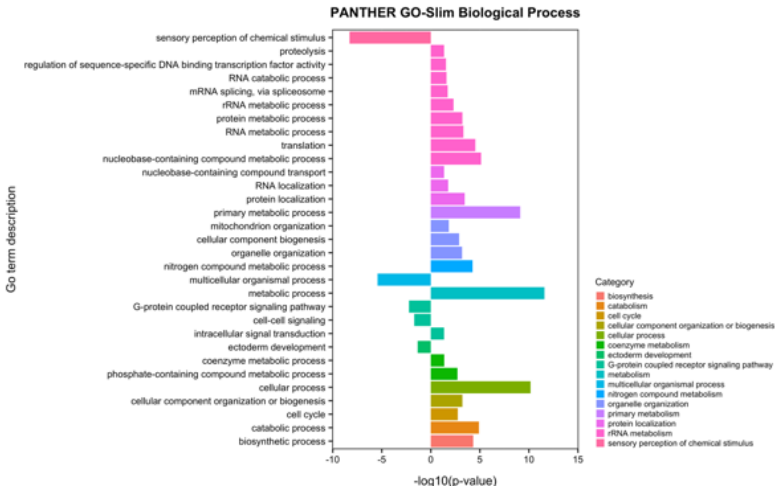


## Supplementary Figure 50.

**Biological processes enriched for ASE genes.** A positive value denotes enrichment and a negative value denotes depletion.

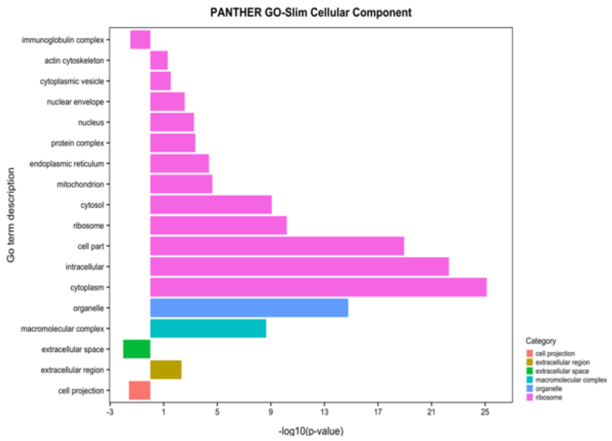


## Supplementary Figure 51.

**Biological processes enriched for ASE genes.** A positive value denotes enrichment and a negative value denotes depletion.

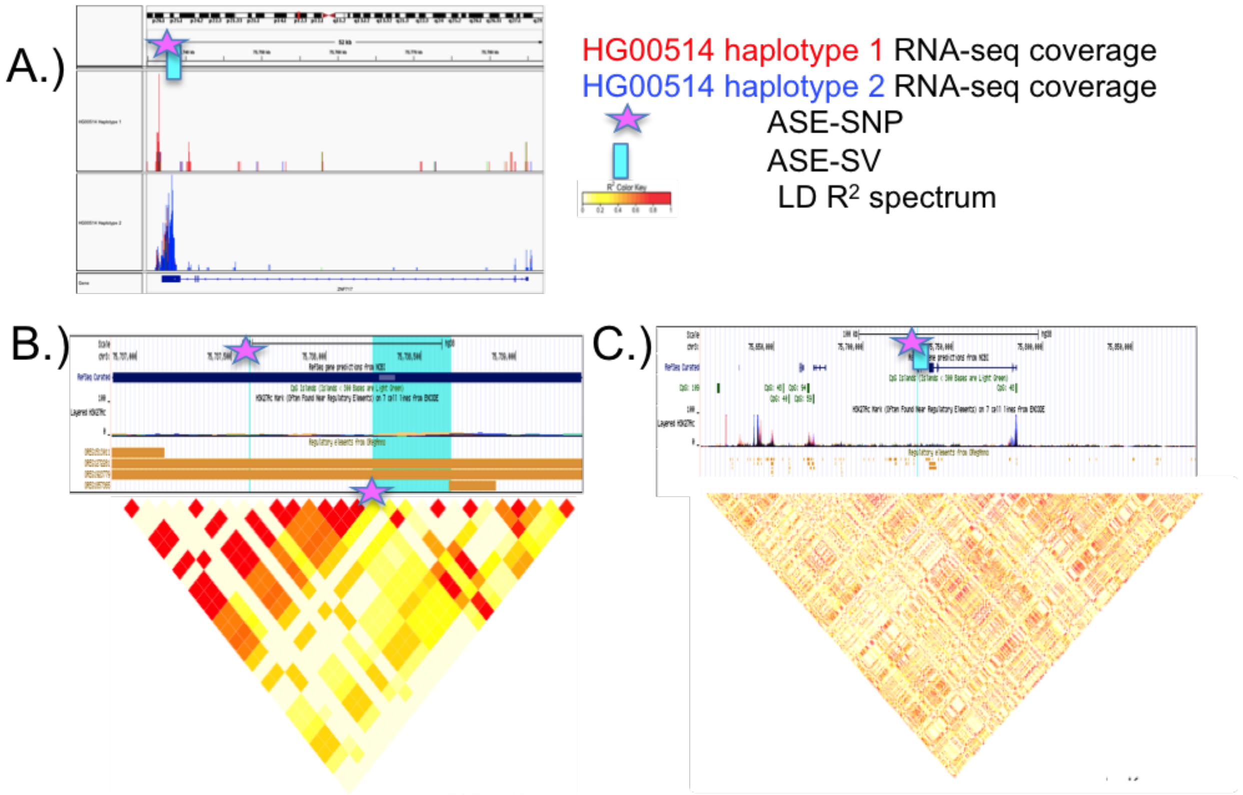


## Supplementary Figure 52.

**Analysis of SNP and PB-SV ASE for indication against a haploblock effect.** A.) IGV plot of phased RNAseq read counts on HG00514 haplotype 1 (upper track) vs. haplotype 2 (lower track) with SV-ASE (PB-DEL, with a genotype of 1|0 meaning deletion on haplotype 1 and no deletion on haplotype 2) on ZNF717 gene; A nearby ASE-SNP was found 644 bp away from the SV-ASE affecting the same gene. B.) $R^2$ calculations for CHS population variants within exon 5 of ZNF717 show local LD surrounding IL-DEL site. C.) LD map for CHS population variants ± 100kb of ZNF717 gene shows little evidence of a regional haploblock effect.

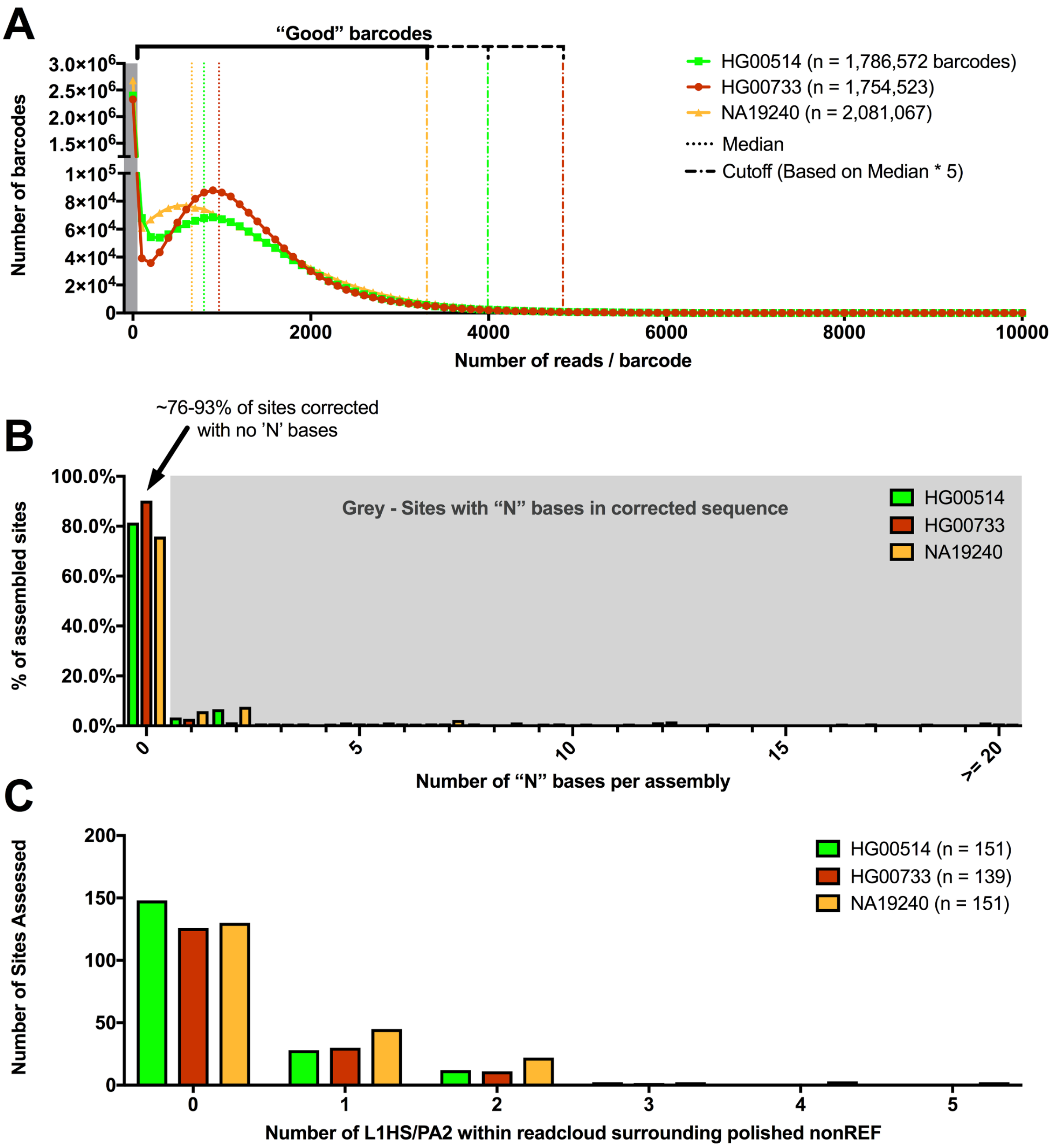


## Supplementary Figure 53.

**Correction of L1 insertions.**

## Supplementary References


1. Clarke, L. *et al.* The international Genome sample resource (IGSR): A worldwide collection of genome variation incorporating the 1000 Genomes Project data. *Nucleic Acids Res.* **45,** D854–D859 (2017).
2. Li, H. Aligning sequence reads, clone sequences and assembly contigs with BWA-MEM. *arXiv [q-bio.GN]* (2013).
3. Hanscom, C. & Talkowski, M. Design of large-insert jumping libraries for structural variant detection using Illumina sequencing. *Curr. Protoc. Hum. Genet.* **80,** 7.22.1–9 (2014).
4. Andrews, S. & Others. FastQC: a quality control tool for high throughput sequence data. (2010).
5. Li, H. & Durbin, R. Fast and accurate short read alignment with Burrows–Wheeler transform. *Bioinformatics* (2009).
6. Faust, G. G. & Hall, I. M. SAMBLASTER: fast duplicate marking and structural variant read extraction. *Bioinformatics* **30,** 2503–2505 (2014).
7. Tarasov, A., Vilella, A. J., Cuppen, E., Nijman, I. J. & Prins, P. Sambamba: fast processing of NGS alignment formats. *Bioinformatics* **31,** 2032–2034 (2015).
8. Li, H. *et al.* The Sequence Alignment/Map format and SAMtools. *Bioinformatics* **25,** 2078–2079 (2009).
9. Barnett, D. W., Garrison, E. K., Quinlan, A. R., Strömberg, M. P. & Marth, G. T. BamTools: a C++ API and toolkit for analyzing and managing BAM files. *Bioinformatics* **27,** 1691–1692 (2011).
10. Sanders, A. D., Falconer, E., Hills, M., Spierings, D. C. J. & Lansdorp, P. M. Single-cell template strand sequencing by Strand-seq enables the characterization of individual homologs. *Nat. Protoc.* **12,** 1151–1176 (2017).
11. Tischler, G. & Leonard, S. biobambam: tools for read pair collation based algorithms on BAM files. *Source Code Biol. Med.* **9,** 13 (2014).
12. Hills, M., O'Neill, K., Falconer, E., Brinkman, R. & Lansdorp, P. M. BAIT: Organizing genomes and mapping rearrangements in single cells. *Genome Med.* **5,** 82 (2013).
13. Porubský, D. *et al.* Direct chromosome-length haplotyping by single-cell sequencing. *Genome Res.* **26,** 1565–1574 (2016).
14. Bishara, A. *et al.* Read clouds uncover variation in complex regions of the human genome. *Genome Res.* **25,** 1570–1580 (2015).
15. Bansal, V., Halpern, A. L., Axelrod, N. & Bafna, V. An MCMC algorithm for haplotype assembly from whole-genome

sequence data. *Genome Res.* **18,** 1336–1346 (2008).

16. Dixon, J. R. *et al.* Topological domains in mammalian genomes identified by analysis of chromatin interactions. *Nature* **485,** 376–380 (2012).
17. Lieberman-Aiden, E. *et al.* Comprehensive mapping of long-range interactions reveals folding principles of the human genome. *Science* **326,** 289–293 (2009).
18. Selvaraj, S., R Dixon, J., Bansal, V. & Ren, B. Whole-genome haplotype reconstruction using proximity-ligation and shotgun sequencing. *Nat. Biotechnol.* **31,** 1111–1118 (2013).
19. McKenna, A. *et al.* The Genome Analysis Toolkit: a MapReduce framework for analyzing next-generation DNA sequencing data. *Genome Res.* **20,** 1297–1303 (2010).
20. Bansal, V. & Bafna, V. HapCUT: an efficient and accurate algorithm for the haplotype assembly problem. *Bioinformatics* **24,** i153–9 (2008).
21. Blumenthal, I. *et al.* Transcriptional consequences of 16p11.2 deletion and duplication in mouse cortex and multiplex autism families. *Am. J. Hum. Genet.* **94,** 870–883 (2014).
22. Sugathan, A. *et al.* CHD8 regulates neurodevelopmental pathways associated with autism spectrum disorder in neural progenitors. *Proc. Natl. Acad. Sci. U. S. A.* **111,** E4468–77 (2014).
23. Wu, T. D. & Nacu, S. Fast and SNP-tolerant detection of complex variants and splicing in short reads. *Bioinformatics* **26,** 873–881 (2010).
24. DeLuca, D. S. *et al.* RNA-SeQC: RNA-seq metrics for quality control and process optimization. *Bioinformatics* **28,** 1530–1532 (2012).
25. Wang, L., Wang, S. & Li, W. RSeQC: quality control of RNA-seq experiments. *Bioinformatics* **28,** 2184–2185 (2012).
26. Quinlan, A. R. & Hall, I. M. BEDTools: a flexible suite of utilities for comparing genomic features. *Bioinformatics* **26,** 841–842 (2010).
27. Aken, B. L. *et al.* The Ensembl gene annotation system. *Database* **2016,** (2016).
28. Broman, K. W., Murray, J. C., Sheffield, V. C., White, R. L. & Weber, J. L. Comprehensive human genetic maps: individual and sex-specific variation in recombination. *Am. J. Hum. Genet.* **63,** 861–869 (1998).
29. Kirkness, E. F. *et al.* Sequencing of isolated sperm cells for direct haplotyping of a human genome. *Genome Res.* **23,**

826–832 (2013).

30. Hou, Y. *et al.* Genome analyses of single human oocytes. *Cell* **155,** 1492–1506 (2013).

31. Lu, S. *et al.* Probing meiotic recombination and aneuploidy of single sperm cells by whole-genome sequencing. *Science* **338,** 1627–1630 (2012).

32. Porubsky, D., Garg, S., Sanders, A. D., Korbel, J. O. & Guryev, V. Dense And Accurate Whole-Chromosome Haplotyping Of Individual Genomes. *bioRxiv* (2017).

33. Klambauer, G. *et al.* cn.MOPS: mixture of Poissons for discovering copy number variations in next-generation sequencing data with a low false discovery rate. *Nucleic Acids Res.* **40,** e69 (2012).

34. Garg, S., Martin, M. & Marschall, T. Read-based phasing of related individuals. *Bioinformatics* **32,** i234–i242 (2016).

35. Huddleston, J. *et al.* Discovery and genotyping of structural variation from long-read haploid genome sequence data. *Genome Res.* (2016). doi:10.1101/gr.214007.116

36. 1000 Genomes Project Consortium *et al.* A global reference for human genetic variation. *Nature* **526,** 68–74 (2015).

37. Ye, K., Schulz, M. H., Long, Q., Apweiler, R. & Ning, Z. Pindel: a pattern growth approach to detect break points of large deletions and medium sized insertions from paired-end short reads. *Bioinformatics* **25,** 2865–2871 (2009).

38. DePristo, M. A. *et al.* A framework for variation discovery and genotyping using next-generation DNA sequencing data. *Nat. Genet.* **43,** 491–498 (2011).

39. Garrison, E. & Marth, G. Haplotype-based variant detection from short-read sequencing. *arXiv [q-bio.GN]* (2012).

40. Danecek, P. *et al.* The variant call format and VCFtools. *Bioinformatics* **27,** 2156–2158 (2011).

41. Sudmant, P. H. *et al.* An integrated map of structural variation in 2,504 human genomes. *Nature* **526,** 75–81 (2015).

42. Lee, C., Grasso, C. & Sharlow, M. F. Multiple sequence alignment using partial order graphs. *Bioinformatics* **18,** 452–464 (2002).

43. Kronenberg, Z. N. *et al.* Wham: Identifying Structural Variants of Biological Consequence. *PLoS Comput. Biol.* **11,** e1004572 (2015).

44. Chiang, C. *et al.* SpeedSeq: ultra-fast personal genome analysis and interpretation. *Nat. Methods* **12,** 966–968 (2015).

45. Layer, R. M., Chiang, C., Quinlan, A. R. & Hall, I. M. LUMPY: a probabilistic framework for structural variant

discovery. *Genome Biol.* **15,** R84 (2014).

46. Rausch, T. *et al.* DELLY: structural variant discovery by integrated paired-end and split-read analysis. *Bioinformatics* **28,** i333–i339 (2012).
47. Sudmant, P. H. *et al.* Diversity of human copy number variation and multicopy genes. *Science* **330,** 641–646 (2010).
48. Sudmant, P. H. *et al.* Global diversity, population stratification, and selection of human copy-number variation. *Science* **349,** aab3761 (2015).
49. Handsaker, R. E., Korn, J. M., Nemesh, J. & McCarroll, S. A. Discovery and genotyping of genome structural polymorphism by sequencing on a population scale. *Nat. Genet.* **43,** 269–276 (2011).
50. Brand, H. *et al.* Paired-Duplication Signatures Mark Cryptic Inversions and Other Complex Structural Variation. *Am. J. Hum. Genet.* **97,** 170–176 (2015).
51. Collins, R. L. *et al.* Defining the diverse spectrum of inversions, complex structural variation, and chromothripsis in the morbid human genome. *Genome Biol.* **18,** 36 (2017).
52. Kent, W. J. BLAT--the BLAST-like alignment tool. *Genome Res.* **12,** 656–664 (2002).
53. Hormozdiari, F., Alkan, C., Eichler, E. E. & Sahinalp, S. C. Combinatorial algorithms for structural variation detection in high-throughput sequenced genomes. *Genome Res.* **19,** 1270–1278 (2009).
54. Hormozdiari, F., Hajirasouliha, I., McPherson, A., Eichler, E. E. & Sahinalp, S. C. Simultaneous Structural Variation Discovery in Multiple Paired-End Sequenced Genomes. in *Research in Computational Molecular Biology* (eds. Bafna, V. & Sahinalp, S. C.) **6577,** 104–105 (Springer Berlin Heidelberg, 2011).
55. Michaelson, J. J. & Sebat, J. forestSV: structural variant discovery through statistical learning. *Nat. Methods* **9,** 819–821 (2012).
56. Chen, X. *et al.* Manta: rapid detection of structural variants and indels for germline and cancer sequencing applications. *Bioinformatics* **32,** 1220–1222 (2016).
57. Zhao, X., Emery, S. B., Myers, B., Kidd, J. M. & Mills, R. E. Resolving complex structural genomic rearrangements using a randomized approach. *Genome Biol.* **17,** 126 (2016).
58. Zhao, X., Weber, A. M. & Mills, R. E. A recurrence-based approach for validating structural variation using long-read sequencing technology. *Gigascience* **6,** 1–9 (2017).

59. Chong, Z. *et al.* novoBreak: local assembly for breakpoint detection in cancer genomes. *Nat. Methods* **14,** 65–67 (2016).

60. Schrider, D. R., Houle, D., Lynch, M. & Hahn, M. W. Rates and genomic consequences of spontaneous mutational events in Drosophila melanogaster. *Genetics* **194,** 937–954 (2013).

61. Gardner, E. J. *et al.* The Mobile Element Locator Tool (MELT): Population-scale mobile element discovery and biology. *Genome Res.* (2017). doi:10.1101/gr.218032.116

62. Hormozdiari, F. *et al.* Alu repeat discovery and characterization within human genomes. *Genome Res.* **21,** 840–849 (2011).

63. Bailey, J. A. & Eichler, E. E. Primate segmental duplications: crucibles of evolution, diversity and disease. *Nat. Rev. Genet.* **7,** 552–564 (2006).

64. Chaisson, M. J. P., Wilson, R. K. & Eichler, E. E. Genetic variation and the de novo assembly of human genomes. *Nat. Rev. Genet.* **16,** 627–640 (2015).

65. Koren, S. *et al.* Canu: scalable and accurate long-read assembly via adaptive k-mer weighting and repeat separation. *Genome Res.* **27,** 722–736 (2017).

66. Chin, C.-S. *et al.* Nonhybrid, finished microbial genome assemblies from long-read SMRT sequencing data. *Nat. Methods* **10,** 563–569 (2013).

67. Huddleston, J. *et al.* Reconstructing complex regions of genomes using long-read sequencing technology. *Genome Res.* **24,** 688–696 (2014).

68. Antonacci, F. *et al.* A large and complex structural polymorphism at 16p12.1 underlies microdeletion disease risk. *Nat. Genet.* **42,** 745–750 (2010).

69. Liao, Y., Smyth, G. K. & Shi, W. featureCounts: an efficient general purpose program for assigning sequence reads to genomic features. *Bioinformatics* **30,** 923–930 (2014).

70. Mortazavi, A., Williams, B. A., McCue, K., Schaeffer, L. & Wold, B. Mapping and quantifying mammalian transcriptomes by RNA-Seq. *Nat. Methods* **5,** 621–628 (2008).

71. Strimmer, K. A unified approach to false discovery rate estimation. *BMC Bioinformatics* **9,** 303 (2008).

72. McLaren, W. *et al.* The Ensembl Variant Effect Predictor. *Genome Biol.* **17,** 122 (2016).

73. Pastinen, T. Genome-wide allele-specific analysis: insights into regulatory variation. *Nature Reviews Genetics* **11,** 533–538 (2010).

74. Serre, D. *et al.* Differential allelic expression in the human genome: a robust approach to identify genetic and epigenetic cis-acting mechanisms regulating gene expression. *PLoS Genet.* **4,** e1000006 (2008).

75. A.S. Dimas, et al. Modifier effects between regulatory and protein-coding variation. *PLoS Genet* **4,** e1000244 (2008).

76. van de Geijn, B., McVicker, G., Gilad, Y. & Pritchard, J. K. WASP: allele-specific software for robust molecular quantitative trait locus discovery. *Nat. Methods* **12,** 1061–1063 (2015).

77. Storey, J. D., Taylor, J. E. & Siegmund, D. Strong control, conservative point estimation and simultaneous conservative consistency of false discovery rates: a unified approach. *J. R. Stat. Soc. Series B Stat. Methodol.* **66,** 187–205 (2004).

78. Dobin, A. *et al.* STAR: ultrafast universal RNA-seq aligner. *Bioinformatics* **29,** 15–21 (2013).

79. Kukurba, K. R. *et al.* Allelic expression of deleterious protein-coding variants across human tissues. *PLoS Genet.* **10,** e1004304 (2014).

80. Chen, J. *et al.* A uniform survey of allele-specific binding and expression over 1000-Genomes-Project individuals. *Nat. Commun.* **7,** 11101 (2016).

81. Lappalainen, T. *et al.* Transcriptome and genome sequencing uncovers functional variation in humans. *Nature* **501,** 506–511 (2013).

82. Pirinen, M. *et al.* Assessing allele-specific expression across multiple tissues from RNA-seq read data. *Bioinformatics* **31,** 2497–2504 (2015).

83. Stranger, B. E. *et al.* Patterns of cis regulatory variation in diverse human populations. *PLoS Genet.* **8,** e1002639 (2012).

84. Dixon, A. L. *et al.* A genome-wide association study of global gene expression. *Nat. Genet.* **39,** 1202–1207 (2007).

85. Pickrell, J. K. *et al.* Understanding mechanisms underlying human gene expression variation with RNA sequencing. *Nature* **464,** 768–772 (2010).

86. Battle, A. *et al.* Genomic variation. Impact of regulatory variation from RNA to protein. *Science* **347,** 664–667 (2015).

87. Gutierrez-Arcelus, M. *et al.* Passive and active DNA methylation and the interplay with genetic variation in gene regulation. *Elife* **2,** e00523 (2013).

88. Wen, X., Luca, F. & Pique-Regi, R. Cross-population joint analysis of eQTLs: fine mapping and functional annotation. *PLoS Genet.* **11,** e1005176 (2015).

89. Montgomery, S. B. *et al.* Transcriptome genetics using second generation sequencing in a Caucasian population. *Nature* **464,** 773–777 (2010).

90. Li, Y. I. *et al.* RNA splicing is a primary link between genetic variation and disease. *Science* **352,** 600–604 (2016).

91. Grubert, F. *et al.* Genetic Control of Chromatin States in Humans Involves Local and Distal Chromosomal Interactions. *Cell* **162,** 1051–1065 (2015).

92. MacArthur, J. *et al.* The new NHGRI-EBI Catalog of published genome-wide association studies (GWAS Catalog). *Nucleic Acids Res.* **45,** D896–D901 (2017).

93. Degner, J. F. *et al.* Effect of read-mapping biases on detecting allele-specific expression from RNA-sequencing data. *Bioinformatics* **25,** 3207–3212 (2009).

94. Battle, A. et al. Characterizing the genetic basis of transcriptome diversity through RNA-sequencing of 922 individuals. Genome research 24, 14-24 (2014).

95. Mi, H., et al. PANTHER version 11: expanded annotation data from Gene Ontology and Reactome pathways, and data analysis tool enhancements. Nucleic acids research 45(D1), D183-D189 (2017).

96. Gene Ontology Consortium. Expansion of the Gene Ontology knowledgebase and resources. Nucleic acids research 45(D1), D331-D338 (2017).

97. Supek, F., et al. REVIGO summarizes and visualizes long lists of gene ontology terms. PLoS One 6, e21800 (2011).

98. Catalogue of imprinted genes. URL, http://igc.otago.ac.nz/home.html.

99. Imprinted gene database. URL, http://www.geneimprint.com/site/genes-by-species.Homo+sapiens.

100. Baran, Y. et al. The landscape of genomic imprinting across diverse adult human tissues. Genome research 25(7), 927-936 (2015).

101. McDaniell, R., et al. Heritable individual-specific and allele-specific chromatin signatures in humans. Science 328(5975),235-239 (2010).

102. Boissinot, S., Chevret, P. & Furano, A. V. L1 (LINE-1) retrotransposon evolution and amplification in recent human history. *Mol. Biol. Evol.* **17,** 915–928 (2000).

103. Beck, C. R. *et al.* LINE-1 Retrotransposition Activity in Human Genomes. *Cell* **141,** 1159–1170 (2010).

104. Tubio, J. M. C. *et al.* Mobile DNA in cancer. Extensive transduction of nonrepetitive DNA mediated by L1 retrotransposition in cancer genomes. *Science* **345,** 1251343 (2014).

105. Lutz, S. M., Vincent, B. J., Kazazian, H. H., Jr, Batzer, M. A. & Moran, J. V. Allelic heterogeneity in LINE-1 retrotransposition activity. *Am. J. Hum. Genet.* **73,** 1431–1437 (2003).

106. Seleme, M. del C. *et al.* Extensive individual variation in L1 retrotransposition capability contributes to human genetic diversity. *Proc. Natl. Acad. Sci. U. S. A.* **103,** 6611–6616 (2006).

107. Dombroski, B., Mathias, S., Nanthakumar, E., Scott, A. & Kazazian, H. Isolation of an active human transposable element. *Science* **254,** 1805–1808 (1991).

108. Marçais, G. & Kingsford, C. A fast, lock-free approach for efficient parallel counting of occurrences of k-mers. *Bioinformatics* **27,** 764–770 (2011).
