# Supplementary material for: Multi-platform discovery of haplotype-resolved structural variation in human genomes
Source: Nat Commun. 2019 Apr 16;10:1784. doi: 10.1038/s41467-018-08148-z (PMC6467913; doi:10.1038/s41467-018-08148-z)
Supplement: Supplementary file 2 — Description of Additional Supplementary Files [file 41467_2018_8148_MOESM2_ESM.pdf]

Description of Additional Supplementary Files

Supplementary Data 1. Sequencing statistics.

Supplementary Data 2. Phasing summary.

Supplementary Data 3. Phase partitioned PacBio reads.

Supplementary Data 4. Summary of assemblies from Phased-SV and MsPAC.

Supplementary Data 5. N50 contig lengths of haplotype-phased assemblies.

Supplementary Data 6. Refinement of meiotic breakpoints using Strand Seq and PacBio reads.

Supplementary Data 7. Indel counts by size.

Supplementary Data 8. Ilumina SV detection algorithms.

Supplementary Data 9. Simple inversions.

Supplementary Data 10. Inverted duplications.

Supplementary Data 11. Large insert clone validated misassemblies in the human reference (inversions).

Supplementary Data 12. Critical regions overlapping with an inversion.

Supplementary Data 13. Summary statistics for FL-L1 elements with 2 intact ORFs.

Supplementary Data 14. Highly differentiated SVs within genes.

Supplementary Data 15. Population genetics of SV.

Supplementary Data 16. SNVs with high LD to eQTL sites.

Supplementary Data 17. Properties of unified callset intersection with exons and UTRs.

Supplementary Data 18. Categorization of gene affecting SV.

Supplementary Data 19. Indels in genes.

Supplementary Data 20. Genes within inversions.

Supplementary Data 21. Illumina concordance by size.

Supplementary Data 22. Summary of assemblies from Phased-SV and MsPAC.

Supplementary Data 23. The basepair quality value.

Supplementary Data 24. Filtering of SVs based on read support.

Supplementary Data 25. Overlap between PacBio assembly based callers and BioNanoGenomics calls.

Supplementary Data 26. simple reciprocal overlap merge of MsPAC and PhasedSV calls.

Supplementary Data 27. Correlation between MS-PAC and Phased-SV SV calls when grouped by 100 kbp bins across the euchromatic genome.

Supplementary Data 28. PhasedSV, MsPAC merging summary.

Supplementary Data 29. Number of tandem repeat cluster loci per haplotype.

Supplementary Data 30. Summary of SV classes from PB-INT callsets.

Supplementary Data 31. Name and corresponding publications of Illumina discovery methods adopted in the integration pipeline.

Supplementary Data 32. Number of SVs reported by individual Illumina algorithms.

Supplementary Data 33. Number of SVs resulted from the Illumina integration pipeline.

Supplementary Data 34. 1,090 (6.8%) of HySA SV calls missed by Pacbio consensus set.

Supplementary Data 35. gain in sensitivity for PB-SV only calls was largely in SVs less than 2kbp.
Supplementary Data 36. Concordance for individual Illumina SV detection algorithms.
Supplementary Data 37. Haplotype support of inversions.
Supplementary Data 38. Summary of genotype results for the Strand-seq inversion discovery set.
Supplementary Data 39. Possibly misoriented sequences in the reference.
Supplementary Data 40. Total number of Strand-seq reads phased by PacBio used in the composite files.
Supplementary Data 41. Number of inversions supported by the intersection test per individual.
Supplementary Data 42. Strand-seq genotype set, performed on unfiltered inversions in the discovery sets.
Supplementary Data 43. Strand-seq genotype set, performed on inversions supported by the interception test.
Supplementary Data 44. list of simple inversions.
Supplementary Data 45. list of genes intersect with simple inversions.
Supplementary Data 46. list of complex inversions.
Supplementary Data 47. list of genes intersect with complex inversions.
Supplementary Data 48. PCR-free high-coverage alignment summary.
Supplementary Data 49. liWGS alignment summary statistics.
Supplementary Data 50. PacBio Sequencing Depth.
Supplementary Data 51. Sequence and alignment summary of TrueSeq Synthetic Long Reads(TSLR) sequences.
Supplementary Data 52. Technology Summary.
Supplementary Data 53. Refinement of meiotic breakpoints using Strand Seq and PacBio reads.
Supplementary Data 54. The total clusters and clusters within tandem repeats, as well as the number of clusters of SVs within tandem repeats when randomly shuffling cluster locations.
Supplementary Data 55. A comparison of the number of calls made by different alignment methods, BLASR and NGM-LR in tandem rpeeat regions.
Supplementary Data 56. Number of SVs from assembly based approaches by genomic region and sequence type.
Supplementary Data 57. Number and type of SV called by WhamG or Lumpy.
Supplementary Data 58. Diversity panel used for dCGH.
Supplementary Data 59. Evaluation of potential Mendelian violations with calls in at least one child.
Supplementary Data 60. liWGS inheritance patterns.
Supplementary Data 61. Summary of SV discovered by liWGS in HGSVC trios.
Supplementary Data 62. Resolved segmental duplication bases in Phased-SV assemblies.
Supplementary Data 63. Copy number polymorphisms in duplications resolved by PacBio assemblies.
Supplementary Data 64. Exons with SVs detected in PB-SV and not IL-SV.
Supplementary Data 65. Number of deletion SVs overlapping conserved elements and transcription factor binding sites (TFBS).
Supplementary Data 66. Large insert clone validated misassemblies in the human reference (inversions).
Supplementary Data 67. Delly genotyping mendelian error rate.

Supplementary Data 68. High-coverage PCR free population samples used for genotyping sequence-resolved variants.

Supplementary Data 69. Number of variants genotyped for each sample.

Supplementary Data 70. Genotyper summary of call proportions by sample and type for non-tandem-repeat variants.

Supplementary Data 71. Mendelian inheritance for the three trios outside tandem repeat loci.

Supplementary Data 72. Disrupted genic content by SVs of different types.

Supplementary Data 73. Numbers of IL-indels in various functional types.

Supplementary Data 74. List of imprinted SNP-ASE genes.

Supplementary Data 75. Summary of SV ASE results.
