# Supplementary material for: Multi-platform discovery of haplotype-resolved structural variation in human genomes
Source: Nat Commun. 2019 Apr 16;10:1784. doi: 10.1038/s41467-018-08148-z (PMC6467913; doi:10.1038/s41467-018-08148-z)
Supplement: Supplementary file 4 — Reporting Summary [file 41467_2018_8148_MOESM4_ESM.pdf]

nature research

Corresponding author(s): Jan Korbel, Evan Eichler, and Charles Lee

Reporting Summary

Nature Research wishes to improve the reproducibility of the work that we publish. This form provides structure for consistency and transparency in reporting. For further information on Nature Research policies, see Authors & Referees and the Editorial Policy Checklist.

Statistical parameters

When statistical analyses are reported, confirm that the following items are present in the relevant location (e.g. figure legend, table legend, main text, or Methods section).

n/a	Confirmed	
☐	☒	The exact sample size (n) for each experimental group/condition, given as a discrete number and unit of measurement
☐	☒	An indication of whether measurements were taken from distinct samples or whether the same sample was measured repeatedly
☐	☒	The statistical test(s) used AND whether they are one- or two-sided<br>*Only common tests should be described solely by name; describe more complex techniques in the Methods section.*
☒	☐	A description of all covariates tested
☐	☒	A description of any assumptions or corrections, such as tests of normality and adjustment for multiple comparisons
☐	☒	A full description of the statistics including central tendency (e.g. means) or other basic estimates (e.g. regression coefficient) AND variation (e.g. standard deviation) or associated estimates of uncertainty (e.g. confidence intervals)
☒	☐	For null hypothesis testing, the test statistic (e.g. F, t, r) with confidence intervals, effect sizes, degrees of freedom and P value noted<br>*Give P values as exact values whenever suitable.*
☒	☐	For Bayesian analysis, information on the choice of priors and Markov chain Monte Carlo settings
☒	☐	For hierarchical and complex designs, identification of the appropriate level for tests and full reporting of outcomes
☒	☐	Estimates of effect sizes (e.g. Cohen's d, Pearson's r), indicating how they were calculated
☐	☒	Clearly defined error bars<br>*State explicitly what error bars represent (e.g. SD, SE, CI)*

Our web collection on statistics for biologists may be useful.

Software and code

Policy information about availability of computer code

Data collection	http://www.internationalgenome.org/data-portal/data-collection/structural-variation
Data analysis	https://github.com/mchaisso/phasedsv; https://bitbucket.org/oscarlr/mspac; https://github.com/xuefzhao/HGSV_SV_integration_pipe; https://github.com/drashley/InversionAnalysis_HGSVC

For manuscripts utilizing custom algorithms or software that are central to the research but not yet described in published literature, software must be made available to editors/reviewers upon request. We strongly encourage code deposition in a community repository (e.g. GitHub). See the Nature Research guidelines for submitting code & software for further information.

Data

Policy information about availability of data

All manuscripts must include a data availability statement. This statement should provide the following information, where applicable:

- Accession codes, unique identifiers, or web links for publicly available datasets
- A list of figures that have associated raw data
- A description of any restrictions on data availability

Underling sequencing read data from the various platforms can be accessed via the International Genome Sample Resource (IGSR) (Clarke et al. 2017) at http://

www.internationalgenome.org/data-portal/data-collection/structural-variation. Indel variant calls will be made available with dbSNP build B151. SV calls are made available under dbVar accession nstd152. All other relevant data is available upon request.

Field-specific reporting

Please select the best fit for your research. If you are not sure, read the appropriate sections before making your selection.

☒ Life sciences ☐ Behavioural & social sciences ☐ Ecological, evolutionary & environmental sciences

For a reference copy of the document with all sections, see nature.com/authors/policies/ReportingSummary-flat.pdf

Life sciences study design

All studies must disclose on these points even when the disclosure is negative.

Sample size	Nine. We chose three parent–child trios (mother, father and child): a Han Chinese (CHS) trio (HG00513, HG00512, HG00514), a Puerto Rican (PUR) trio (HG00732, HG00731, HG00733) and a Yoruban (YRI) Nigerian trio (NA19238, NA19239, NA19240).
Data exclusions	No data excluded.
Replication	All DNAs are accessible through Coriell and genomic data was obtained using the procedures indicated in the manuscript. The sequence analyses can be replicated using the analysis flow that has been posted on github.
Randomization	Each trio is selected randomly from the population.
Blinding	All the samples were sequenced by the same protocols and analyzed by the same pipeline. There is no bias applied.

Reporting for specific materials, systems and methods

Materials & experimental systems

n/a	Involved in the study
☒	☐ Unique biological materials
☒	☐ Antibodies
☐	☒ Eukaryotic cell lines
☒	☐ Palaeontology
☒	☐ Animals and other organisms
☐	☒ Human research participants

Methods

n/a	Involved in the study
☒	☐ ChIP-seq
☒	☐ Flow cytometry
☒	☐ MRI-based neuroimaging

Eukaryotic cell lines

Policy information about cell lines

Cell line source(s)	All cell lines used in this study (HG00732, HG00731, HG00733, NA19238, NA19239, NA19240, HG00513, HG00512, HG00514) can be obtained from the Coriell Biorepository.
Authentication	SNP variants obtained from our sequencing data was compared with previously published data and used to authenticate each cell line.
Mycoplasma contamination	All cell lines were tested for mycoplasm and shown to be negative for mycoplasm presence.
Commonly misidentified lines (See ICLAC register)	N/A

Human research participants

Policy information about studies involving human research participants

Population characteristics	We chose three parent–child trios (mother, father and child): a Han Chinese (CHS) trio (HG00513, HG00512, HG00514), a Puerto Rican (PUR) trio (HG00732, HG00731, HG00733) and a Yoruban (YRI) Nigerian trio (NA19238, NA19239, NA19240). The Han Chinese and Yoruban Nigerian families were representative of low and high genetic diversity genomes, respectively, while the Puerto Rican family was chosen to represent an example of population admixture.

Recruitment

Transformed lymphoblast cell lines from three parent-child trios belonging to the 1000 Genomes Project were obtained from the Coriell Cell Repository as part of the NHGRI catalog.
